# Four single-basepair mutations in the *ptx* promoter of *Bordetella bronchiseptica* are sufficient to activate the expression of pertussis toxin

**DOI:** 10.1038/s41598-021-88852-x

**Published:** 2021-04-30

**Authors:** Qing Chen, Mary C. Gray, Erik Hewlett, Scott Stibitz

**Affiliations:** 1 Division of Bacterial, Parasitic, and Allergenic Products, Center for Biologics Evaluation and Research, Food and Drug Administration, Silver Spring, MD 20993 USA; 2 Division of Infectious Diseases, Department of Medicine, University of Virginia, Charlottesville, VA 22908 USA

**Keywords:** Evolution, Microbiology, Pathogenesis

## Abstract

Secretion of pertussis toxin (PT) is the preeminent virulence trait of the human pathogen *Bordetella pertussis*, causing whooping cough. *Bordetella bronchiseptica*, although it harbors an intact 12-kb *ptx–ptl* operon, does not express PT due to an inactive *ptx* promoter (P*ptx*), which contains 18 SNPs (single nucleotide polymorphisms) relative to *B. pertussis* P*ptx*. A systematic analysis of these SNPs was undertaken to define the degree of mutational divergence necessary to activate *B. bronchiseptica* P*ptx*. A single change (C^−13^T), which created a better − 10 element, was capable of activating *B. bronchiseptica* P*ptx* sufficiently to allow secretion of low but measureable levels of active PT. Three additional changes in the BvgA-binding region, only in the context of C^−13^T mutant, raised the expression of PT to *B. pertussis* levels. These results illuminate a logical evolutionary pathway for acquisition of this key virulence trait in the evolution of *B. pertussis* from a *B. bronchiseptica*-like common ancestor.

## Introduction

*Bordetella pertussis* is a strictly human pathogen and the etiological agent of the disease known as whooping cough or pertussis, a highly contagious acute respiratory infection, often perceived as a primarily childhood disease. While pertussis was clearly much more prevalent before the implementation of widespread childhood vaccination in the 1950s, and while infants and young children clearly have borne, and continue to bear, the brunt of the morbidity and mortality associated with pertussis, adolescent and adult infections are more common than previously believed^1^ and resurgence of pertussis, even in highly vaccinated populations, is a growing public health concern^2,3^. *B. pertussis* is closely related to two other species which together make up the ‘classical’ *Bordetellae*. *B. bronchiseptica* is primarily a veterinary pathogen and infects a wide range of mammals, resulting in milder or asymptomatic chronic respiratory infections^4^. *B. parapertussis* causes pertussis-like disease in humans, with a separate branch causing infections in sheep^5,6^. Independent phylogenetic studies indicate that these three pathogens evolved from a common ancestor that was more “bronchiseptica-like”^7,8^.

The majority of known or suspected *Bordetellae* virulence factors are produced by all of the ‘classical’ *Bordetellae* and include adhesins such as filamentous hemagglutinin (FHA), fimbriae (Fim), and pertactin (Prn) as well as toxins such as adenylate cyclase toxin (Cya), dermonecrotic toxin (Dnt), and tracheal cytotoxin (TCT) [^9^ and references therein]. However, pertussis toxin (PT), which is also a major antigen and vaccine component^10^, is produced exclusively by *B. pertussis*. PT is an A–B type toxin composed of the five subunits S1–S5, in a ratio of 1:1:1:2:1, which are encoded by the *ptxABDEC* genes^11^. The holotoxin contains one A subunit (S1), possessing ADP-ribosylase transferase enzymatic activity, with the B-oligomer formed by the remaining subunits^12^. Secretion of PT requires the type IV secretion system made up of nine Ptl proteins encoded by the *ptlABCDIEFGH* genes, located immediately downstream of the *ptx* genes. The *ptx* and *ptl* genes are co-transcribed under the control of the *B. pertussis ptx* promoter (P*ptx*^*per*^)^13,14^. Like other virulence gene promoters in the *Bordetellae*, P*ptx*^*per*^ is positively regulated by the two-component system BvgAS, composed of the histidine-kinase sensor protein BvgS, and the response regulator protein BvgA. As a result, under modulating conditions, such as in the presence of MgSO_4_ or nicotinic acid, or at low temperatures, P*ptx*^*per*^ is not activated, the *ptx–ptl* operon is not transcribed, and PT is not secreted^15–17^.

**Figure 1:**
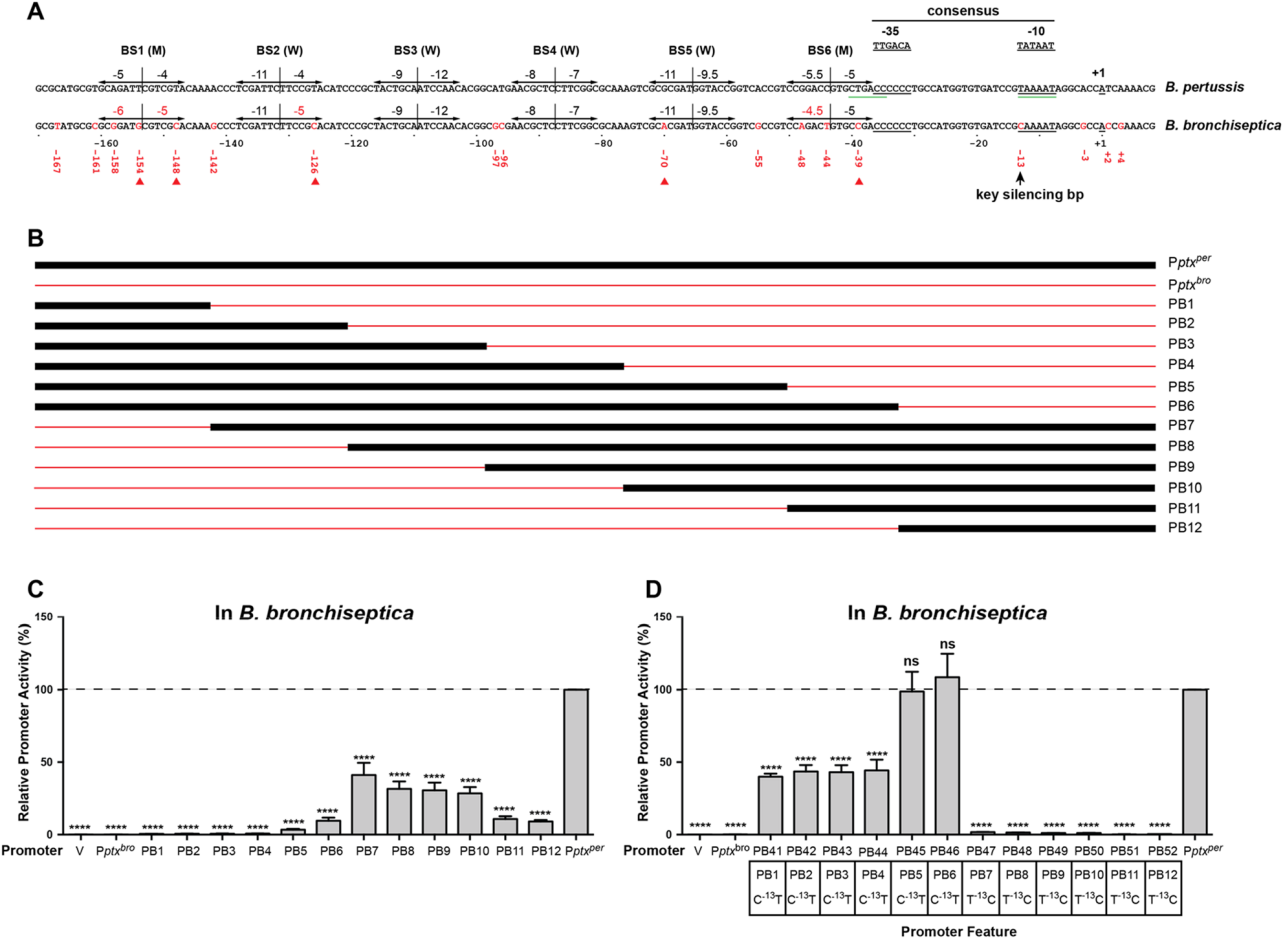
SNPs in both the core promoter and the BvgA-binding region dictate differences in activity between P*ptx*^*per*^ and P*ptx*^*bro*^. (**A**) The DNA sequences of the *ptx* promoter regions containing 18 SNPs are shown for *B. pertussis* Tohama I and *B. bronchiseptica* RB50*.* The transcription start site^51^, − 10 region and − 35 region^22^ in P*ptx*^*per*^ are underlined in black. The previously inferred − 10 and − 35 regions of P*ptx*^*per*^ are underlined in green^51^. The consensus − 10 and − 35 regions for *E. coli* sigma 70 promoter core regions are displayed above. The six BvgA ~ P dimer binding sites (BS), revealed by our recent study on P*ptx*^*per*^^22^, are indicated by head-to-head arrows and scored for predicted binding strength as reported therein. Based on those predictions, predicted intermediate binding strength sites are labeled with an “M” and weak ones with a “W”. Within the nucleotide sequence 18 SNPs are colored red in P*ptx*^*bro*^ and their locations relative to the transcription start are given in red underneath. The key basepair C^−13^ silencing P*ptx*^*bro*^ is indicated. Five SNPs in P*ptx*^*bro*^, C^−39^, A^−70^, C^−126^, C^−148^ and G^−154^, whose replacement with their P*ptx*^*per*^, counterparts was shown to increase P*ptx*^*bro*^ activity in the context of C^−13^T in Fig. 5A, are indicated by red triangles. (**B**) Differing extents of *B. pertussis* and *B. bronchiseptica* sequences in hybrid promoters are indicated in black for P*ptx*^*per*^ and red for P*ptx*^*bro*^. DNA sequences of hybrid promoters are provided in Supplemental Fig. S1. (**C**,**D**) Plasmids containing hybrid promoters PB1–PB12 and PB41–PB52, fused to *luxCDABE* in vector pSS3967, were integrated into *B. bronchiseptica* RB50 to generate ectopic *lux* fusions, with their resulting luminescence shown here. Plasmids pQC1284 and pQC1114 were used as wild type controls for P*ptx*^*bro*^ and P*ptx*^*per*^, respectively, and pSS3967 was used as an empty vector control (V). For the PB41 to PB52 hybrid promoters shown in (**D**) the additional promoter feature of C^−13^T is indicated in the boxed region. Integrants were grown on BG agar without 50 mM MgSO_4_ for 1 day at 37 °C and light output was measured and analyzed as described in “Materials and methods” section. Relative *lux* expression is reported as a percentage of the maximally expressed promoter P*ptx*^*per*^. Each value represents the average of at least four independent assays. Using P*ptx*^*per*^ as a control group, results of each group were analyzed for significance by ordinary one-way ANOVA Dunnett’s multiple comparison tests as described in “Materials and methods” section. *ns* not significant; ****P ≤ 0.0001.

As a late gene promoter, the concentration of BvgA ~ P required for activation of P*ptx*^*per*^, is higher than that for early gene promoters, such as P*fha*^17,18^, suggesting that BvgA ~ P binding to the *ptx* promoter is of lower affinity than that to P*fha*. In addition, in vitro and in vivo studies have demonstrated that BvgA ~ P is both necessary and sufficient for P*ptx*^*per*^ transcriptional activation^19–21^. Due to BvgA ~ P’s weaker interaction with the *ptx* promoter, details of its binding have been difficult to establish and methods that were used to investigate these aspects of higher affinity promoters have not been successful. Only recently has a detailed picture of BvgA ~ P binding to the *ptx* promoter emerged^22^. This study utilized a mutant BvgA protein that displayed increased affinity to P*ptx* and which was labelled with FeBABE^22^. Site specific cleavage patterns at P*ptx* revealed binding of six dimers of BvgA ~ P, as shown in Fig. 1A. The dimers are each formed of two monomers in a head-to-head configuration, and these dimers bind to DNA with a spacing of 22 bp on-center. This geometry of binding is similar to that observed at other BvgA-activated promoters such as P*fha*^23^ and P*fim3*^24^, which bind three, and two, dimers of BvgA ~ P, respectively, with the most upstream binding site being of higher affinity. As at the *fha* promoter, the binding site of the most promoter-proximal BvgA molecule in P*ptx* abuts the − 35 sequence of the core *ptx* promoter which, in the context of this more precise picture of BvgA binding, can be seen to be the hexamer CCCCCC. This unusual − 35 sequence is spaced at the optimal 17 bp from the unmistakable − 10 sequence (TAAAAT) rather than the 21 bp spacing invoked in previous analyses to accommodate a putative − 35 hexamer displaying a better match to the consensus. However, the suboptimal nature of this actual − 35 sequence is necessary for regulation of P*ptx* by BvgA^22^. Apart from the number of binding sites, the other most salient difference of P*ptx* when compared to P*fha* is the apparent affinity of those binding sites, which ranges from very poor to moderate, with only three of the binding sites actually contributing to BvgA-activation^22^.

Although they are incapable of expressing pertussis toxin, the other classical *Bordetella* species, *B. bronchiseptica* and *B. parapertussis*, may carry an entire, intact *ptx*–*ptl* operon^8,25^. For one typical *B. bronchiseptica* strain, this operon was demonstrated to be fully functional, leading to the secretion of active PT when an active promoter was supplied upstream^26^. The toxin produced was further shown to be neutralized by pertussis vaccine-induced antibody^27^. Lack of expression pertussis toxin is instead due to a number of single nucleotide polymorphisms (SNPs), relative to P*ptx*^*per*^*,* in the *ptx* promoters of these non-toxigenic species, *B. bronchiseptica* P*ptx* (P*ptx*^*bro*^) and *B. parapertussis* P*ptx* (P*ptx*^*para*^). A total of 18 SNPs are present between the *ptx* promoters of *B. pertussis* Tohama I, and *B. bronchiseptica* RB50, as shown in Fig. 1A. Based on our current knowledge of the promoter architecture of *B. pertussis* P*ptx*^*per*^, these SNPs are found in both the BvgA ~ P binding region and the core promoter region. In order to have a more complete understanding of how the active, BvgA-regulated P*ptx*^*per*^ could have evolved from the non-BvgA-activated P*ptx*^*bro*^, leading to the acquisition of in vivo pertussis toxin expression by an evolving human pathogen, we sought to determine how many, and which, of the 18 SNPs present in P*ptx*^*per*^ actually contribute to this key phenotypic difference.

## Results

### The ‘silent’ *ptx* promoter of *B. bronchiseptica* directs minimal but detectable levels of transcription

**Figure 2:**
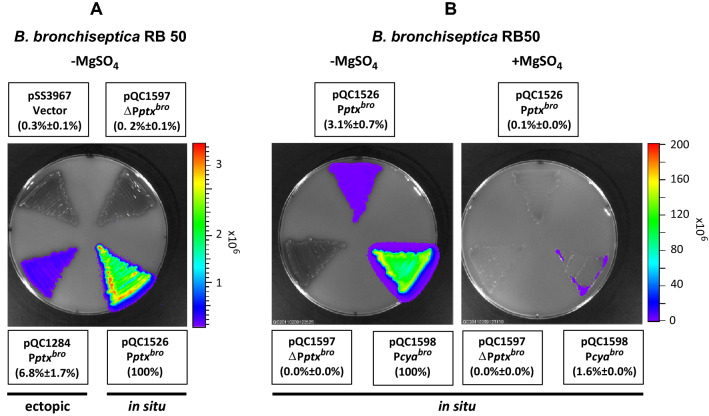
Low but detectable P*ptx*^*bro*^ transcriptional activity in *B. bronchiseptica.* (**A**) Plasmid pQC1284 containing P*ptx*^*bro*^ (− 290 to + 28), fused to *luxCDABE* in vector pSS3967, was integrated into *B. bronchiseptica* RB50 to generate an ectopic *lux* fusion (bottom left), and the plasmid pQC1526 containing a larger region of P*ptx*^*bro*^ (− 1170 to + 28), fused to *luxCDABE* in the vector pSS4162, was integrated into *B. bronchiseptica* RB50 to generate an in situ* lux* fusion in its native context (bottom right). The empty vector pSS3967 was integrated into *B. bronchiseptica* RB50 to serve as a negative control for the ectopic fusion (Vector, top left), and plasmid pQC1597 containing a deletion of P*ptx*^*bro*^ (− 167 to + 4) from pSS1526 was integrated into *B. bronchiseptica* RB50 to serve as negative control for the in situ fusion (ΔP*ptx*^*bro*^, top right). (**B**) Plasmid pQC1598 containing P*cya*^*bro*^ and upstream sequences (− 930 ~ + 37), fused to *luxCDABE* in vector pSS4162, was integrated into *B. bronchiseptica* RB50 to generate an in situ P*cya*^*bro*^*-lux* fusion. This strain, together with those from (**A**), harboring ectopic and in situ P*ptx*^*bro*^*-lux* fusions, were grown on BG agar without (left) or with (right) 50 mM MgSO_4_ for 1 day at 37 °C. Light production was measured and analyzed as for (**A**) and as described in “Materials and methods” section. Light output was expressed as a percentage relative to RB50::pQC1526 (in situ P*ptx*^*bro*^*-lux*) in (**A**) and RB50::pQC1598 (in situ P*cya*^*bro*^*-lux*) in (**B**) without MgSO_4_. Reported values represent the average of at least four independent assays for each strain.

Previous studies found that expression of *ptx* genes in *B. bronchiseptica* was not detectable either at the RNA level by mRNA dot blot hybridization, at the protein level by ELISA, or by biological activity in a CHO cell assay^25,26^. This functional silence of the *ptx* operon is apparently the result of an inefficient promoter^25^. At the outset of the current study, in order to establish a baseline of expression, we evaluated the activity of the *ptx* promoter from *B. bronchiseptica* (P*ptx*^*bro*^) through the use of transcriptional gene fusions to the luciferase operon (*luxCDABE*) of *Photorhabdus luminescens*. We have previously employed such fusions in gene expression studies and found them to be especially useful for detecting low levels of activity, due to their inherently low background. To introduce a P*ptx-lux* fusion into the *B. bronchiseptica* strain RB50, we cloned a P*ptx*^*bro*^-containing DNA fragment, which extended from nucleotides − 1170 to + 28 (relative to the transcriptional start site), into the previously described suicide plasmid vector pSS4162^28^, upstream of the promoter-less *lux* operon (*luxCDABE*), thus creating pQC1526. Insertion of this plasmid into a *Bordetella* genome, via homologous recombination, places the *lux* operon downstream of the native *ptx–ptl* promoter in the correct orientation, with all upstream sequences intact. We designate this type of fusion, introduced at the native genomic location of a given promoter, as “in situ” to distinguish it from the *“*ectopic” type of fusion described next. An ectopic fusion was introduced by cloning a smaller, “stand-alone” P*ptx*^*bro*^ fragment (− 290 to + 28) upstream of the promoter-less *lux* operon in the suicide plasmid vector pSS3967 described previously^24,28^ thus creating pQC1284. Insertion of pSS3967 derivatives also occurs by homologous recombination, but between a plasmid-borne segment of the *Bordetella* chromosome, and its counterpart at an arbitrary but fixed location in the bacterial genome. As shown in Fig. 2A, in both in situ (RB50::pQC1526) and ectopic (RB50::pQC1284) contexts, P*ptx*^*bro*^ demonstrated low but detectable transcriptional activity. The promoter-less negative controls in both cases indicated clearly that this activity is due to P*ptx*^*bro*^ and does not represent background activity of the fusion vector. Of note is the observation that the level of expression from the in situ fusion was approximately 15 times higher than that of the ectopic fusion. We have previously observed such differences in maximal activity between ectopic and in situ fusions^22^. The basis of these differences is not currently understood. As shown in Fig. 2B, in the in situ contexts, P*ptx*^*bro*^ (pQC1526) was observed to have about 3% of the activity of P*cya*^*bro*^ (pQC1598) and could be modulated by the addition of MgSO_4,_ indicating its regulation by *bvgAS*. P*cya* is another Bvg-regulated late promoter that directs the synthesis of adenylate cyclase toxin. Although P*ptx*^*bro*^ directs a detectable and reproducible amount of transcription, that amount is apparently not enough to lead to the synthesis and secretion of active PT (see below).

**Figure 3:**
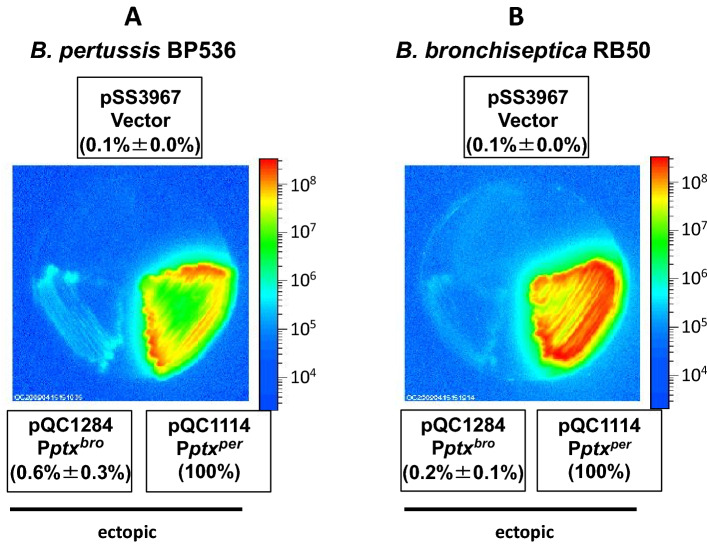
Comparison of the inherent transcriptional activity of P*ptx*^*bro*^ and P*ptx*^*per*^ A**.** Plasmids pQC1284 and pQC1114 containing P*ptx*^*bro*^ and P*ptx*^*per*^, respectively, as *lux* fusions in vector pSS3967 were integrated into *B. pertussis* BP536 (**A**) or *B. bronchiseptica* RB50 (**B**) to generate ectopic fusions. The resulting *B. pertussis* BP536 and *B. bronchiseptica* RB50 strains were grown on BG agar for 2 days or 1 day, respectively, at 37 °C. Light output was observed, analyzed and presented as described for Fig. 2.

We also compared P*ptx*^*bro*^ transcriptional activity to that of P*ptx*^*per*^, which is capable of directing the expression and secretion of large amounts of active PT. Because of the differences in genetic background, including SNPs in their *bvgAS* loci, we performed this comparison in both parental species. As shown in Fig. 3A,B, in either *B. pertussis* BP536 or *B. bronchiseptica* RB50, in an ectopic context, the *B. pertussis ptx* promoter (P*ptx*^*per*^ in pQC1114) had more than 100-fold higher activity than its *B. bronchiseptica* counterpart (P*ptx*^*bro*^ in pQC1284). Interestingly, the fold-difference between the two promoters was greater in *B. bronchiseptica* than in *B. pertussis* (588-fold vs. 178-fold). The very low levels of P*ptx*^*bro*^-directed transcription detected in both strains suggest that its functional silence is an intrinsic feature of the promoter and not dependent on other genetic factors in each host species. Transcriptional activity of P*ptx*^*bro*^ is negligible compared to the fully functional P*ptx*^*per*^ regardless of the *Bordetella* species.

### Functional differences between P*ptx*^*per*^ and P*ptx*^*bro*^ reside in both the core promoter region and the BvgA-binding region

As shown in Fig. 1A, in the region from − 167 to + 4, there are 18 SNPs between the active P*ptx*^*per*^ and the much less active P*ptx*^*bro*^^8,25^. Until recently, lack of information on *ptx* promoter architecture has made ascribing functional significance to these SNPs difficult. However, a recent study using FeBABE-labelled mutant BvgA revealed the geometry of BvgA-binding to this low affinity promoter^22^. The sites to which 6 dimers of BvgA ~ P bind are shown in Fig. 1A. We used this information to inform segment-swapping experiments to create hybrid promoters having differing extents of sequences of either P*ptx*^*per*^ or P*ptx*^*bro*^, with the junction points lying between binding sites. These hybrid promoters, named PB1 to PB12, are diagrammed in Fig. 1B and their activities relative to P*ptx*^*per*^ are shown in Fig. 1C. Examining PB1 to PB6, it can be seen that substituting P*ptx*^*bro*^, from the upstream boundary, with increasing extents of P*ptx*^*per*^, had little effect on activity unless all, as in PB6, or nearly all, as in PB5, of the BvgA binding region was substituted with that of P*ptx*^*per*^. PB6 achieved approximately 10% of the activity of P*ptx*^*per*^. Conversely, similarly substituting P*ptx*^*per*^ with sequences from P*ptx*^*bro*^ resulted in hybrid promoters that maintained significant activity, dropping to about 10% of P*ptx*^*per*^ in the hybrids that juxtaposed the P*ptx*^*per*^ core promoter region to the entire (PB12), or nearly entire, (PB11), P*ptx*^*bro*^ BvgA binding region. These results indicate that the core promoter region, that harbors 4 SNPs, and the entire upstream BvgA-binding region, containing 14 SNPs, contributed to a similar degree to P*ptx*^*per*^ activity. We therefore proceeded to dissect each of these regions separately.

### A single nucleotide change in the core promoter region can significantly activate P*ptx*^*bro*^

**Figure 4:**
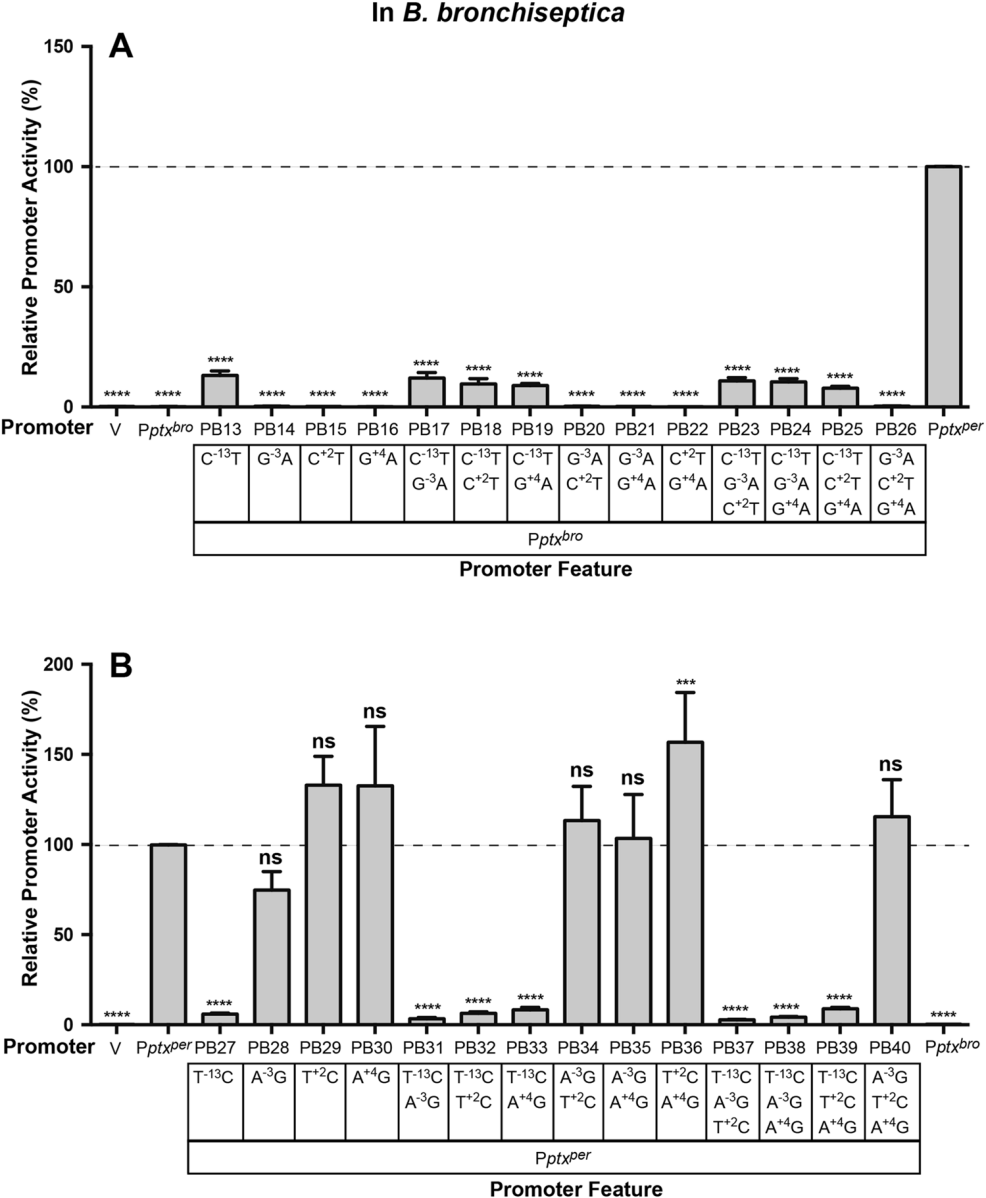
Identification of a crucial SNP in the P*ptx* core promoter Plasmids containing hybrid promoters PB13–PB26, which encompass all possible combinations of the P*ptx*^*per*^ versions of the four SNPs found in the core promoter, introduced into the context of P*ptx*^*bro*^ (**A**) and PB27–PB41 which encompass a similar set in which the P*ptx*^*bro*^ SNPs have been introduced into P*ptx*^*per*^ (**B**), were integrated as ectopic *lux* fusions in pSS3967 into *B. bronchiseptica* RB50. SNPs for each promoter are indicated in the boxed region. The wild type P*ptx*^*bro*^, P*ptx*^*per*^, and vector (V) controls, as well as methods for data analysis using the values from P*ptx*^*per*^ as a control group, are the same as for Fig. 1.

The core region contains four SNPs, at positions − 13, − 3, + 2, and + 4, relative to the start of transcription. To determine which of these contributed to differences in promoter activity, we constructed variants of P*ptx*^*bro*^ in which all possible combinations of the P*ptx*^*per*^ versions of these SNPs (C^−13^T, G^−3^A, C^+2^T, and G^+4^A), were introduced. As shown in Fig. 4A for PB13–PB26, only those variants in which the − 13 SNP (C^−13^T) was incorporated showed measurable activity. That activity was comparable in all, indicating that the − 13 SNP alone was responsible for the increase. In a converse fashion, introducing all possible combinations of the P*ptx*^*bro*^ versions of these nucleotides into P*ptx*^*per*^ (T^−13^C, A^−3^G, T^+2^C, and A^+4^G) resulted in PB27–PB40. The activities of these hybrid promoters, shown in Fig. 4B, indicated that all of the variants that included the − 13 change (T^−13^C), and only those variants, were reduced to less than 10% of the activity of wild type P*ptx*^*per*^. As shown in Fig. 1A, the effect of the − 13 SNP is to create a better fit to the σ^70^-dependent consensus − 10 sequence (TATAAT) in P*ptx*^*per*^ (TAAAAT) and a poorer one in P*ptx*^*bro*^ (CAAAAT). In light of both the theoretical, and demonstrable functional, importance of the − 13 SNP, we reconstructed the hybrid promoters PB1–PB12 to contain a T at position − 13 in PB1–PB6, to create PB41–PB46, and a C at position − 13 in PB7–PB12 to create PB47–PB52. As shown for PB46 in Fig. 1D, with reference to PB6 in Fig. 1C, changing its C at position − 13 to a T allows the P*ptx*^*bro*^ core region to function with the upstream sequences from P*ptx*^*per*^ as effectively as in the P*ptx*^*per*^ wild type promoter. Conversely changing the T at position − 13 in the P*ptx*^*per*^ core region of PB12 to a C, as in PB52, destroys its ability to provide up to approximately 10% of wild type P*ptx*^*per*^ activity in conjunction with the P*ptx*^*bro*^ BvgA-binding region, reducing it to a level comparable to P*ptx*^*bro*^. Taken together, these data demonstrate that the − 13 SNP is the only SNP in the core region that makes a significant impact and furthermore that it is a key basepair in determining P*ptx* activity, as its substitution alone can increase P*ptx*^*bro*^ activity to 10% that of P*ptx*^*per*^.

### Several SNPs in the P*ptx* BvgA binding region contribute to the greater activity of P*ptx*^*per*^

**Figure 5:**
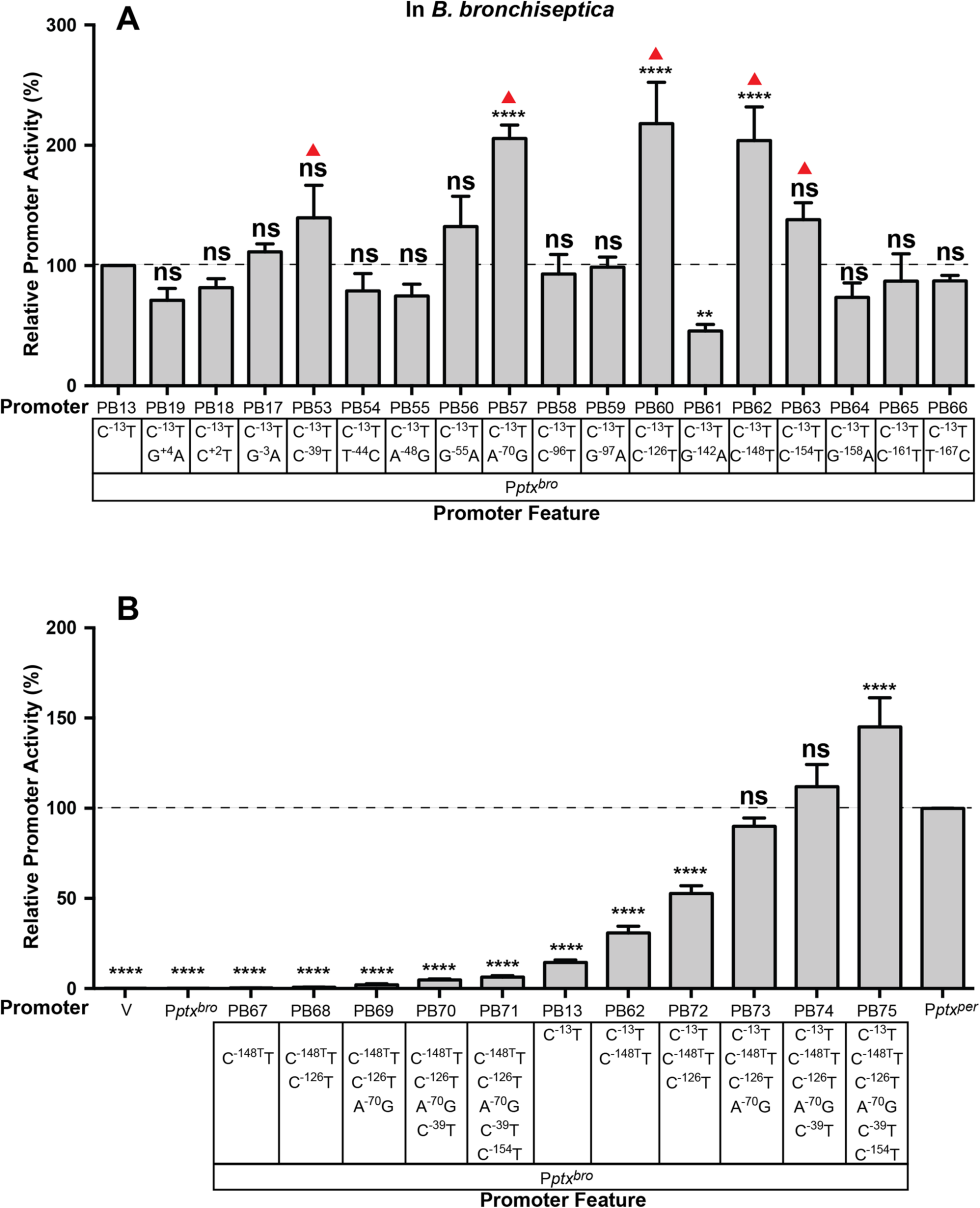
Assessment of the impact of individual SNPs in the BvgA-binding region on the function of P*ptx*^*bro*^ and P*ptx*^*bro*^ C^−13^T. Plasmids containing various hybrid promoters fused to *luxCDABE* in vector pSS3967 were integrated into *B. bronchiseptica* RB50 to generate ectopic fusions, and the resulting light output was measured as described in Fig. 4. SNPs present in each promoter are shown in the boxes below each graph. (**A**) The P*ptx*^*per*^ versions of each SNP in the BvgA-binding region were introduced singly into P*ptx*^*bro*^ C^−13^T. Five of these, which had the greatest effect and are marked in red triangle, were chosen for further analysis. (**B**) The five SNPs identified in (**A**) were introduced stepwise, in an additive fashion, into P*ptx*^*bro*^, as in hybrid promoters PB67–PB71 or into P*ptx*^*bro*^ C^−13^T, as in hybrid promoters PB62 and PB72–PB75. The wild type P*ptx*^*bro*^, P*ptx*^*per*^, and vector (V) controls, as well as methods for data analysis using the values from PB13 (**A**) or P*ptx*^*per*^ (**B**) as a control group, are as in Fig. 1.

To determine which of the 14 SNPs in the BvgA binding region dictate differences in activity between P*ptx*^*bro*^ and P*ptx*^*per*^, we individually introduced each of the P*ptx*^*per*^ versions of these SNPs into the P*ptx*^*bro*^ C^−13^T promoter. As shown in Fig. 5A for PB53–PB66, seven of these resulted in observable increases in promoter activity, although only three reached statistical significance in this experiment. We selected five of the most active, i.e. those at − 39, − 70, − 126, − 148, − 154, in PB53, PB57, PB60, PB62, and PB63, respectively, for further study. When these five changes were added consecutively to P*ptx*^*bro*^ in the order − 148, − 126, − 70, − 39, − 154 (Fig. 5B, PB67–PB71), activity increased only slightly. The maximal level that was obtained, in the quintuply substituted promoter PB71, was still less than 5% of P*ptx*^*per*^ wild type levels. However, when these same five substitutions were added consecutively to P*ptx*^*bro*^ in the context of the C^−13^T substitution (Fig. 5B, PB62, PB72–PB75), a different picture emerged. With only three additional substitutions (PB73), promoter activity was nearly that of wild type P*ptx*^*per*^. A fourth (PB74) and a fifth substitution (PB75) raised activity to levels higher than wild type P*ptx*^*per*^. These consecutive mutations demonstrate a theoretically possible mutational path whereby, with a limited number of mutations, P*ptx*^*bro*^ could be altered to increase expression to a level comparable to that in *B. pertussis*.

### A small number of mutational changes activate expression of PT in *B. bronchiseptica*

**Figure 6:**
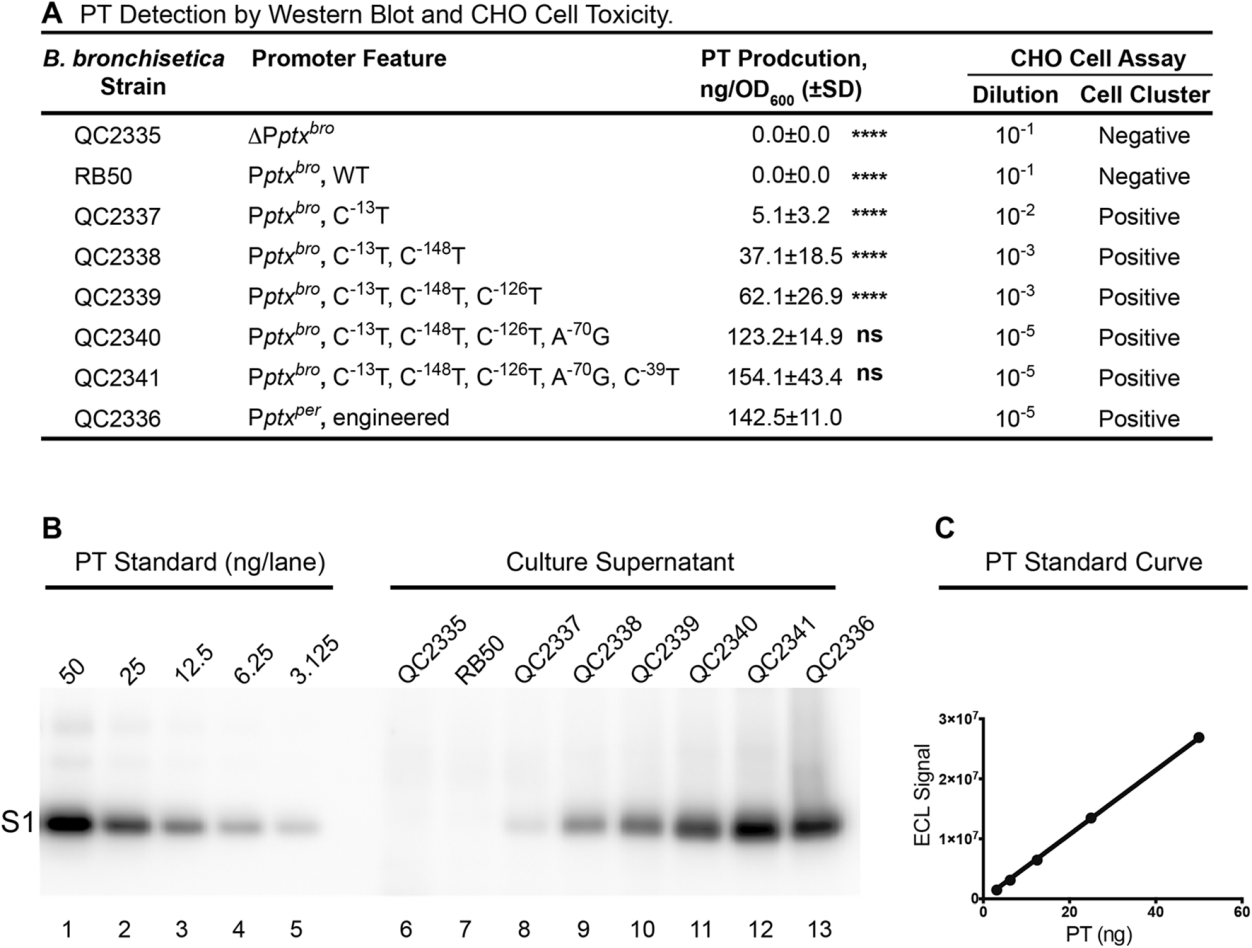
A small number of mutations in *B. bronchiseptica* RB50 P*ptx* can activate expression of functional PT*.* (**A**) Production of PT by wild-type and derivatives of *B. bronchiseptica* RB50 was determined by Western blot as described in (**B**,**C**) and shown in the third column. Values presented represent the mean and range from two independent cultures analyzed in this fashion. Statistical analysis of the results, using the values from QC2336 as a control group, is as in Fig. 1. Assessment of biological activity was performed by the CHO cell clustering assay as described in “Materials and methods” section and is presented in columns four and five. Supernatants analyzed by the CHO cell assay were the same ones assessed on the Western blot shown in (**B**) lanes 6–13. (**B**) Western blot probed with a monoclonal antibody against the pertussis toxin S1 subunit was performed following SDS PAGE analysis of known amounts of purified pertussis toxin containing all five subunits (lanes 1–5) or samples derived from culture supernatants (lanes 6–13). Culture supernatants and TCA-precipitated protein samples derived from them were obtained following growth of wild type *B. bronchiseptica* RB50, and derivatives containing mutations in the *ptx* promoter region, in LB plus streptomycin as described in “Materials and methods” section. (**C**) Densitometric analysis of lanes 1–5 provided data to produce a PT standard curve, shown here, which was then used to determine the mass per culture density (OD) of PT produced by different strains.

To more definitively demonstrate activation of pertussis toxin expression in *B. bronchiseptica* by the type of mutational pathway determined here, we used allelic exchange techniques to alter the nucleotide sequence of the *ptx* promoter of *B. bronchiseptica* RB50 in its native context. Production of PT was assessed by Western Blot, using a monoclonal antibody that recognizes the pertussis toxin S1 subunit. As shown in Fig. 6A, incorporation of the single basepair C^−13^T mutation into RB50 in strain QC2337 (P*ptx*^*bro*^, C^−13^T) resulted in production of detectable amounts of PT secreted into the culture supernatant, as compared to none in the wild type *B. bronchiseptica* RB50 and the *ptx* promoter deletion strain QC2335 (ΔP*ptx*^*bro*^). The amount of PT production so detected by the Western blot (Fig. 6B, lanes 6–13) was quantitated densitometrically and converted to mass units using a standard curve (Fig, 6C) plotted using data from Fig. 6B, lanes 1–5 in which known quantities of pertussis toxin were analyzed. More pertinently, biological PT activity was also assessed by the standard CHO cell assay. As shown in Fig. 6A, a 100-fold dilution of culture supernatant from strain QC2337 (P*ptx*^*bro*^, C^−13^T) contained detectable PT activity, while none was detected using a tenfold dilution of culture supernatant of the wild type *B. bronchiseptica* RB50 or the *ptx* promoter deletion strain QC2335 (ΔP*ptx*^*bro*^). Further incorporation of upstream mutations in strains QC2338 (P*ptx*^*bro*^, C^−13^T, C^−148^T), QC2339 (P*ptx*^*bro*^, C^−13^T, C^−148^T, C^−126^T), QC2340 (P*ptx*^*bro*^, C^−13^T, C^−148^T, C^−126^T, A^−70^G) and QC2341 (P*ptx*^*bro*^, C^−13^T, C^−148^T, C^−126^T, A^−70^G, C^−39^T), increased the level of PT expression to a level comparable to that resulting from introduction of the wild type *B. pertussis ptx* promoter in strain QC2336 (P*ptx*^*per*^, engineered), as determined by both Western blot and the CHO cell assay (Fig. 6A,B). Together, these results demonstrate that a small number of mutational changes are sufficient to render a non-toxigenic *B. bronchiseptica* toxigenic to a level comparable to *B. pertussis*.

### Activation of P*ptx* during Bordetella evolution

**Figure 7:**
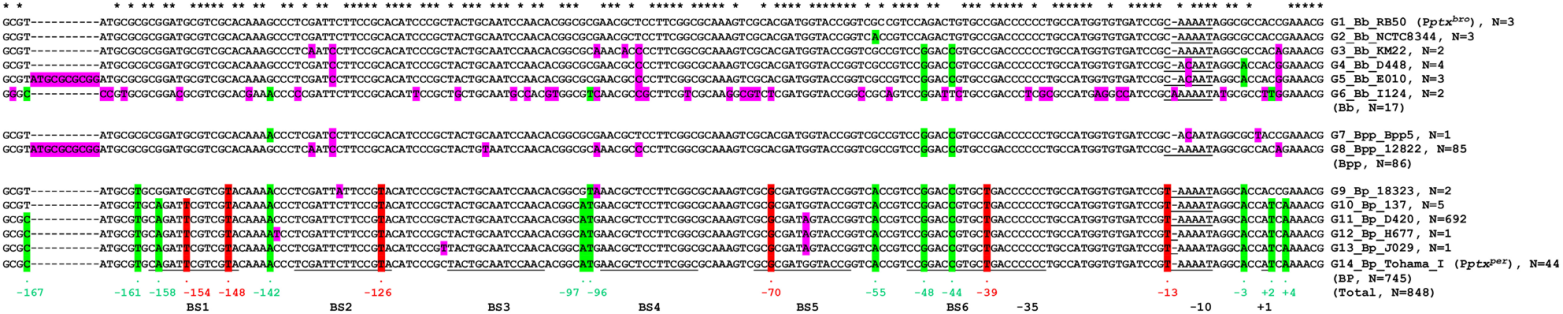
*Bordetella* P*ptx* sequence comparison. Different *ptx* promoter sequences, equivalent to − 170 bp to + 9 relative to the P*ptx* + 1 of *B. bronchiseptica* RB50 (genome sequence ID NC_002927.3, nucleotides from 5,198,768 to 5,198,946), were obtained from 848 complete genomes of *Bordetella,* aligned and grouped based on their sequence as described in “Materials and methods” section. More detailed strain information is available in Table S1. BvgA-binding sites BS1–BS6, and promoter elements − 35, − 10 and + 1 are underlined. Positions in the sequence at which no SNPs were found are marked with an asterisk above the RB50 sequence. SNPs, relative to RB50, shown in this work to contribute to P*ptx* activation in Tohama I (G14), are highlighted in red and those not shown to so contribute are highlighted in green. Remaining SNPs, or insertions, relative to RB50, present in *B. bronchiseptica* (Bb), *B. parapertussis* (Bpp) and *B. pertussis* (Bp) strains, are highlighted in pink.

The functional analysis presented here has focused on the *ptx* promoters of only one strain each of *B. bronchiseptica* and *B. pertussis*. To place these results in a larger context, we analyzed the presence and sequence of P*ptx* in additional strains and species. Because the *ptx/ptl* genes are absent in some species of *Bordetella* and some *B. bronchiseptica* strains^7,29^, we first queried the 957 complete *Bordetella* genomes in the BLAST database of NCBI (National Center for Biotechnology information) for the presence of sequences similar to a 1076 bp segment from *B. bronchiseptica* RB50 that comprises *ptxA* [the first gene in the *ptx* operon (810 bp)] and its upstream promoter sequences (266 bp). This was done to ensure that promoter sequences undergoing further analysis were actually found in the appropriate genetic context. Among 12 different *Bordetella* species represented in this set of 957 genomic sequences, 848 genome hits were obtained in only three *Bordetella* species*,* namely *B. bronchiseptica* (N = 17/23), *B. parapertussis* (N = 86/86), and *B. pertussis* (N = 745/748), contained sequences similar to the 1076 bp query. No hits were obtained for any of the other, “non-classical”, *Bordetella* species including *B. holmesii* (N = 0/68), *B. hinzii* (N = 0/13), *B. petrii* (N = 0/1), *B. avium* (N = 0/6), *B. trematum* (N = 0/5), *B. pseudohinzii* (N = 0/2), *B. flabilis* (N = 0/1), *B. bronchialis* (N = 0/2), and *B. ansorpii* (N = 0/2). Further sequence analysis of the *ptx* promoter region, from − 170 bp to + 9 relative to the P*ptx* + 1, revealed 14 different promoter sequences among the 848 collected promoter sequences and the strains containing them were termed G1–G14. These sequences, together with representative strains and the number of strains in each group are shown in the comparison presented in Fig. 7 and the identity of all of the strains sampled is found in Table S1. P*ptx*^*pro*^ and P*ptx*^*per*^ are represented by G1_Bb_RB50 and G14_Bp_Tohama_I, respectively. The 18 SNPs present in G14, relative to G1, are highlighted in either red or green in that sequence. Those highlighted in red, specifically those at positions − 154, − 148, − 126, − 70, − 39, and − 13, are the ones shown in this work to contribute to promoter strength, as shown in Fig. 5. The remaining twelve, highlighted in green, were not demonstrated to so contribute. If present in any of the other 12 P*ptx* sequences, they are similarly highlighted in those sequences. Highlighted in pink are all SNPs in all sequences, relative to G1. When presented in this way two major patterns are readily apparent. One is that eight of the SNPs shown in green, specifically those at positions − 167, − 142, − 96, − 55, − 48, − 44, − 3, and + 2, are found in at least one of the *B. bronchiseptica* or *B. parapertussis* sequence groups. This indicates a strong likelihood that at least some of these SNPs were present in the latest common ancestor of these modern classical *Bordetellae* species, in keeping with our current understanding of their evolutionary relationships. However, perhaps more striking is the observation that the SNPs shown in red in Fig. 7, and shown in this work to increase promoter activity, are found only in the *B. pertussis* sequence groups. Additionally, they are all found in all of those sequences with one exception. The SNP at position − 154 is not found in G9, represented by two strains, but is present in 743 of the remaining 745 *B. pertussis* sequences analyzed. Interestingly, G9 is represented by the strain 18323 which, although the ATCC type strain for *B. pertussis*, has been shown to be atypical and to group apart from the majority of *B. pertussis* strains in phylogenic analyses^6^. Although SNPs in the − 10 region were present in some *B. bronchiseptica* and one *B. parapertussis* strains, these differences do not represent a better match to the consensus σ^70^ − 10 element (CAAAAT to CACAAT and CAAAAT to CAAAAA) and are not predicted to increase transcription.

## Discussion

Our knowledge of how human bacterial pathogens evolve has expanded tremendously in recent decades due to the advent of molecular biology, and in more recent years due to the development of high-throughput DNA sequencing technologies and the resulting explosion in available genomic DNA sequence data. These approaches have solidified our understanding of the evolution of bacteria, with human pathogens being of special interest, as a process driven significantly by horizontal transfer of genetic information. Such transfer can allow the acquisition of new traits in a single step. This can result from the genomic integration of one or a few genes, as for those encoding a particular toxin, such as diphtheria toxin or Shiga Toxin^30^ carried on temperate bacteriophages. It can also result from the incorporation of much larger DNA segments, such as the pathogenicity islands of *Salmonella* species, that encode a larger number of genes, whose products interact in highly sophisticated ways to form supramolecular machines such as type III secretion apparatus^31^. The acquisition of virulence plasmids is also commonly seen in pathogenic bacteria, as exemplified by *Shigella* and pathogenic *E. coli*^32,33^. Much less common are clear examples of “old-school” evolution, i.e. the acquisition of new traits through the accumulation of single base-pair changes that alter function. However, it stands to reason that, as humans have evolved from our simian relatives through the accumulation of a relatively small number of regulatory mutations, bacterial pathogens may evolve through the accumulation of mutations that affect the regulation of virulence genes. Such mutations might determine whether or not a trait is expressed, or might alter temporal, spatial, or environmental patterns of expression.

The expression of PT by the human pathogen *Bordetella pertussis* may represent such an example. PT is encoded by 5 genes, *ptxABDEC*. In addition, 9 more genes, *ptlABCDIEFGH*, make up the *ptl* operon, whose encoded proteins mediate the extracellular secretion of functional PT^34^. All genes are transcribed by P*ptx*^*per*^, the *B. pertussis ptx* promoter. Other pathogenic *Bordetella* species, such as *B. bronchiseptica*, and *B. parapertussis*, contain this approximately 12 kb *ptx–ptl* operon, but they do not synthesize PT, at least under typical laboratory growth conditions or in mammalian infection models. This appears to be due to differences in their *ptx* promoter regions. For example, between the sequenced strains *B. bronchiseptica* RB50 and *B. pertussis* Tohama I, there are 18 SNPs, shown in Fig. 1A. Although there are also a large number of SNPs in the coding regions of the *ptx–ptl* operon, none of these are predicted to disrupt open reading frames, and when an active promoter was supplied upstream of a *B. bronchiseptica ptx–ptl* operon, active PT was synthesized and secreted^26^. While these SNPs in the promoter regions of the non-toxigenic *Bordetella* species were originally thought to represent silencing mutations, more recent phylogenetic analyses support a model in which the latest common ancestor of *B. pertussis, B. parapertussis* and *B. bronchiseptica* was more akin to *B. bronchiseptica*^6,7,29^. In this scenario SNPs in the *ptx* promoter region of *B. pertussis* would instead represent mutations that activate this promoter and bring it under the control of the *bvgAS* global virulence regulatory locus, with the result that it is expressed during infection of mammalian hosts, most notably humans.

Such a scenario would be difficult to envision if all 18 SNPs were required for activation of the ancestral *ptx* promoter in the evolution of *B. pertussis*. Even the requirement for as few as three mutations would make the probability of such a triple variant arising spontaneously prohibitively low. Even though the combined selective advantage of such multiple changes might have been very high, it is unlikely that such a mutant could have arisen in a single step such that natural selection could then act upon it. To investigate this question, and to determine if an evolutionary path from P*ptx*^*bro*^ to a more active variant by single steps was feasible, we sought first to determine which of the SNPs in this region contributed to the greater promoter activity of P*ptx*^*per*^ relative to P*ptx*^*bro*^. Our results showed that a single mutation in the core promoter region of P*ptx*^*bro*^, C^−13^T, that improved the − 10 region, resulted in an increase in transcriptional activity of at least 100-fold, to a level approximately 10% that of P*ptx*^*per*^. Because this level of expression results in the secretion of detectable amounts of active pertussis toxin, it could be expected to provide a selective advantage in certain new environments, such as the human host, that a hypothetical evolutionary intermediate was in the process of adapting to. We can also reasonably infer that, for a hypothetical latest common ancestor, access to the human respiratory tract environment would be frequent, given the close association of our human ancestors and their domesticated livestock. Even today persons living and working in close contact with animals have been shown to be transiently colonized by *B. bronchiseptica.*

Once the initial C^−13^T change had occurred, the accumulation of additional mutations in the BvgA-binding region of the promoter could lead to further increases, and therefore could provide further, incremental, selective advantages. Our results here demonstrate that the effects of beneficial mutations in the upstream region are essentially undetectable in the absence of the C^−13^T mutation in the − 10 region. However, in the context of the C^−13^T mutation, a single additional mutation increased promoter activity to ~ 30% of P*ptx*^*per*^ (PB62 in Fig. 5B) and as few as three additional upstream mutations increased the transcriptional activity of P*ptx*^*bro*^ to levels near that of P*ptx*^*per*^ (PB73 in Fig. 5B). We have also demonstrated that mutations that increased transcription of P*ptx*^*bro*^ also resulted in the synthesis of detectable active PT, further demonstrating that transcriptional activity is the limiting step in PT synthesis and secretion in *B. bronchiseptica*.

It should be noted that of the 14 SNPs in the upstream BvgA-binding region, most resulted in no significant change in P*ptx*^*bro*^ activity when examined in isolation in the context of the C^−13^T mutation (Fig. 5A). Of the four which did significantly affect activity three resulted in an increase and one resulted in a decrease. It is not unexpected that mutations occured that had no significant effect. This observation is consistent with the phenomenon of genetic drift, i.e. the fixation in a population, by random processes, of mutations that do not confer a meaningful phenotypic change. However, by examining the binding region mutations individually we were able to identify those that did confer a meaningful phenotype. It is also not surprising that one such mutation had a significant negative effect (G^−142^A). Such a mutation could have occurred, not in isolation, but in the context of other mutations that had a positive effect, so that its negative effect was offset. Such offsetting mutations could have predated the appearance of this negative mutation or could have arisen after and then become fixed due to selective pressure to maintain P*ptx* activity. The three isolated mutations that significantly increased activity in isolation (A^−70^G, C^−126^T, and C^−148^T), when combined, had additive effects that were sufficient, in the context of C^−13^T, to raise promoter activity to nearly that of P*ptx*^*per*^ (PB73 in Fig. 5B). The addition of two additional mutations (C^−39^T, and C^−154^T) which, in the analysis shown in 5A, had conferred a smaller increase that did not achieve statistical significance, in this context incrementally increased promoter activity to a level greater than that of P*ptx*^*per*^ (PB75 in Fig. 5B). With PB75, we have effectively created a derivative of P*ptx*^*per*^ in which all SNPs conferring negative, neutral, or only weakly positive effects have been removed. Our ability to show such a dramatic effect by sequentially combining the effects of only beneficial mutations should not lead to the interpretation that this was the actual sequence of events. However, these results do demonstrate the feasibility of a mutational path in which a series of single basepair changes could have accumulated stepwise, because each could have conferred a selective advantage, leading eventually to high levels of regulated pertussis toxin expression.

A recent study has provided a more precise picture of how BvgA ~ P interacts with the *ptx* promoter^22^, presented in Fig. 1A in terms of the positions of BvgA-binding and their relative predicted affinity. In that study^22^, the positions of binding were revealed through the use of the affinity cleavage reagent FeBABE-BvgA, and the predicted affinity was assessed through the application of an algorithm based on a comprehensive mutational analysis of the high affinity primary binding site for BvgA in the *fha* promoter^35^. Of note, the five mutations analyzed as shown in Fig. 5B and shown to incrementally increase promoter activity, are all within BvgA binding sites. Three of those, at positions − 126, − 148 and − 154, are also predicted to increase binding affinity according to the algorithm previously described^35^. Of the three binding site mutations shown, in isolation, to significantly increase promoter activity, two, A^−70^G, and C^−126^T, are in weaker binding sites BS5 and BS2, respectively. We had previously found that these weaker binding sites could be removed by deletion from P*ptx*^*per*^ without reducing promoter strength^22^. Therefore they would not appear to actively contribute to the overall strength of BvgA binding required for activation. However, when present, these two binding sites must be of a sufficient affinity to permit cooperative interactions of BvgA ~ P bound to them with BvgA ~ P bound to neighboring sites of higher affinity, essentially to bridge between those higher affinity sites. From this perspective it is not surprising that both A^−70^G and C^−126^T are also the sites of mutations beneficial to overall promoter activity. Interpretation of the beneficial effect of the C^−148^T mutation in BS1 is much more straightforward. This binding site apparently serves as a nucleation point for cooperative binding and oligomerization of BvgA ~ P dimers downstream. Thus, an increase in binding affinity of BS1 by the C^−148^T mutation, as predicted, would be expected to result in greater binding to BS1and thus greater occupation of downstream binding sites, including BS6, ultimately leading to increased activation of transcription.

A detailed genetic and functional analysis is by its nature typically confined to a small number of examples of a species or other biological grouping. In our case we limited our investigation specifically to understanding functional differences between the promoters of RB50, representing *B. bronchiseptica*, and Tohama I, representing *B. pertussis*. To expand our view of P*ptx* evolution we examined its presence and sequence in 957 complete Bordetella genomes in the GenBank database. This database encompasses 23 *B. bronchiseptica*, 86 *B. parapertussis*, and 748 *B. pertussis* genomes, as well as those of the more recently described, non-classical, *Bordetellae*, including *B. holmesii* (N = 68), *B. hinzii* (N = 13), *B. petrii* (N = 1), *B. avium* (N = 6), *B. trematum* (N = 5), *B. pseudohinzii* (N = 2), *B. flabilis* (N = 1), *B. bronchialis* (N = 2), *B. ansorpii* (N = 2). The *ptx* promoter, in the context of the first gene of the pertussis toxin operon, was not found in any of the non-classical *Bordetella* genomes but was found in 17 *B. bronchiseptica*, all 86 *B. parapertussis*, and 745 *B. pertussis* genomes. Of the 18 SNPs documented in Tohama I P*ptx*^*per*^, relative to RB50, eight occur in at least one of the sequence groups from *B. bronchiseptica* and *B. parapertussis* (G1–G8 in Fig. 7) and in some of these groups several are present. This observation is in keeping with previous evolutionary analyses indicating divergence, from a common, ancestral, *B. bronchiseptica*-like lineage, of the lineages leading to the toxigenic species *B. pertussis*, and to the closely related non-toxigenic species *B. bronchiseptica* and *B. parapertussis*. Some or all of these eight SNPs may therefore have predated this split. However, the most notable finding is that all six of the SNPs shown in the present study to result in increased pertussis toxin expression were found only in strains of *B. pertussis*. Additionally, they were found in nearly all of the *B. pertussis* genomes examined. One was not present in two of the 745 strains in Fig. 7. Thus, these SNPs are specific to the *B. pertussis* lineage and reflect the presumed selective pressure that the nascent *B. pertussis* species was experiencing as it was adapting to a new niche, the human host, a selective pressure not experienced by members of the lineages of the non-toxigenic classical strains.

While this study illuminates a general set of feasible mutational paths to activation of a “silent” set of genes, ultimately resulting in PT expression in the human host, they remind us of a more puzzling question. The *ptx–ptl* operon is never expressed by *B. bronchiseptica* during colonization of, and growth in, mammalian hosts. Therefore, what selective pressures led originally to its acquisition and then to its conservation during the long periods of time prior to the divergence of the lineage leading to *B. pertussis*? That it is not expressed under such conditions seems to be well established. Despite a great deal of effort, attempts to demonstrate PT expression by *B. bronchiseptica* have been unsuccessful. In vivo efforts have examined serological data in animal models for *B. bronchiseptica* infection. Some tantalizing observations have also come from large scale pertussis vaccine clinical trials, in which it was found that a very few individuals, reportedly infected with *B. bronchiseptica*, had measurable, although low, antibody titers against PT^36^. However, similar findings have not been reported elsewhere, and further investigation of the strains isolated from these patients did not provide evidence of PT expression. We can speculate, based on the ability of *B. bronchiseptica* to survive, and even replicate, in oligotrophic environments that it has a free-living life-style as part of its natural history^37^. Such an environment, for example in a natural watering hole, would provide ready opportunities for inoculation of the upper respiratory tract of the many different animal species using it. This scenario is also consistent with both the wide host-range of *B. bronchiseptica* and the fact that swimming motility is a trait of this species that is expressed in the Bvg^−^ mode induced at typical environmental temperatures^38^. In such environments, pertussis toxin expression could represent a strategy for evading predation by protozoan eukaryotic cells. Following the evolutionary leap to become a strictly human and more acute pathogen, expression of pertussis toxin in this new mammalian environment could have provided a major selective advantage, countering not protozoan predators, but the human immune system in the form of lymphocytes.

The role of pertussis toxin in the natural history of *B. bronchiseptica* currently remains a mystery and hopefully future investigations will shed some light. The current study, however, provides an increased understanding of how its expression could have evolved to enable survival of *B. pertussis* in a new niche, that of the human host.

## Materials and methods

### Bacteria strains and growth media

Bacterial strains and plasmids used in this study are listed in Table 1. *Escherichia coli* strains were grown on Luria–Bertani (LB) agar or in LB broth^39^. *Bordetella* strains were grown on Bordet Gengou (BG) agar containing 15% defibrinated sheep blood and supplemented with 1% proteose peptone (Becton, Dickinson and Company). Unless otherwise indicated, concentrations of antibiotics used in LB agar or broth were: ampicillin, 200 μg/mL; gentamicin sulfate, 5 μg/mL; kanamycin sulfate, 10 μg/mL. Antibiotic concentrations in BG agar were: gentamicin sulfate, 5 μg/mL; streptomycin sulfate, 100 μg/mL; nalidixic acid, 100 μg/mL.

**Table 1:** Bacteria strains and plasmids used in this study.

Strains	Relevant features	Source or reference
***E. coli***		
DH5α	High-efficiency transformation	Bethesda Research Laboratories
SM10	Mobilization proficient, Kan^R^	^46^
S17.1	Mobilization proficient, Str^R^	^46^
***Bordetella***		
Tohama I	*B. pertussis*, patient isolate	^47^
BP536	Tohama I, Str^R^, Nal^R^	^48^
RB50	*B. bronchiseptica*, rabbit isolate, Str^R^	^49^
QC2335	RB50 ΔP*ptx*^*bro*^	This study
QC2331	RB50 ΔP*ptx*^*bro*^::*aacCI*, Gen^R^	This study
QC2336	RB50 P*ptx*^*per*^	This study
QC2337	RB50 P*ptx*^*bro*^, C^−13^T	This study
QC2338	RB50 P*ptx*^*bro*^, C^−13^T, C^−148^T	This study
QC2339	RB50 P*ptx*^*bro*^ C^−13^T, C^−148^T, C^−126^T	This study
QC2340	RB50 P*ptx*^*bro*^, C^−13^T, C^−148^T, C^−126^T, A^−70^G	This study
QC2341	RB50 P*ptx*^*bro*^, C^−13^T, C^−148^T, C^−126^T, A^−70^G, C^−39^T	This study
**Plasmids**		
pSS1827	Helper plasmid with *tra* operon for triparental mating, ColE1 *oriV*, RK2 *oriT*, *bla*	^50^
pSS3967	*luxCDABE* promoter assay vector for ectopic chromosomal integration, ColE1 *oriV*, RK2 *oriT*, *bla*, *aacCI*	^24,28^
pQC1114	pSS3967::P*ptx*^*per*^ (− 290 to + 28)	^22^
pQC1284	pSS3967::P*ptx*^*bro*^ (− 290 to + 28)	This study
PB plasmids	pSS3967::PB1–PB75 (− 290 to + 28)	This study
	PB plasmids are described in Supplementary Table S2	
pSS4162	*luxCDABE* promoter assay vector for in situ chromosomal integration, ColE1 *oriV*, RK2 *oriT*, *bla*, *aacCI*	^28^
pQC1526	pSS4162::P*ptx*^*bro*^ (− 1170 to + 28)	This study
pQC1597	pSS4162::ΔP*ptx*^*bro*^ (− 1170 to + 28)	This study
pQC1598	pSS4162::P*cya*^*bro*^ (− 930 to + 37)	This study
pSS4661	Allelic exchange vector, ColE1 *oriV*, RK2 *oriT*, P*ptx*^*per*^*-*I-*Sce*I gene*,* I-*Sce*I site, *aphA*	This study
pQC1540	pSS4661::P*ptx*^*bro*^ flanking regions (− 753 to + 586) with − 167 to + 4 replaced by 5′-gagacccccgggggtctc-3′ (*Bsa*I-*Sma*I-*Bsa*I)	This study
pQC1541	pSS4661::P*ptx*^*bro*^ (− 753 to + 586) with P*ptx*^*per*^ replacing − 167 to + 4	This study
pQC1542	pSS4661::P*ptx*^*bro*^ (− 753 to + 586), C^−13^T	This study
pQC1543	pSS4661::P*ptx*^*bro*^ (− 753 to + 586), C^−13^T, C^−148^T	This study
pQC1544	pSS4661::P*ptx*^*bro*^ (− 753 to + 586), C^−13^T, C^−148^T, C^−126^T	This study
pQC1545	pSS4661::P*ptx*^*bro*^ (− 753 to + 586), C^−13^T, C^−148^T, C^−126^T, A^−70^G	This study
pQC1546	pSS4661::P*ptx*^*bro*^ (− 753 to + 586), C^−13^T, C^−148^T, C^−126^T, A^−70^G, C^−39^T	This study
pQC1561	pSS4661::P*ptx*^*bro*^ (− 753 ~ + 586) *aacCI* replacing − 167 to + 4	This study

### Plasmid construction, mutagenesis and sequencing

Primers used in this study are provided in Supplemental Table S3. To determine promoter activity in vivo we used plasmid vector pSS3967 (Amp^R^, Gent^R^)^24,28^ which contains the *luxCDABE* operon of *Photorhabdus luminescens*, *oriT* from RK2, and a 1.8 kb chromosomal fragment conserved between *B. pertussis* and *B. bronchiseptica*. Short fragments containing promoters to be assayed were cloned between the *EcoR*I and *Sal*I restriction sites upstream of the promoter-less *lux* genes and downstream of strong transcriptional terminators. Through homologous recombination, these promoter-l*ux* fusion plasmids can be integrated into an ectopic location on the chromosome of either *B. pertussis* BP536 or *B. bronchiseptica* RB50. Both strains are streptomycin-resistant, allowing for selection of exconjugants with streptomycin and gentamicin. PCR fragments flanked by upstream *EcoR*I site and downstream *Sal*I sites, were generated to include *ptx* promoters (− 290 to + 28 relative to the transcription start) from *B. pertussis* Tohama I genome sequence (NC_002929.2) 3,987,943 to 3,987,260, or from *B. bronchiseptica* RB50 genome sequence (NC_002927.3) 5,198,696 to 5,198,965, and were cloned into pSS3967 to create plasmids pQC1114 (P*ptx*^*per*^)^22^ and pQC1284 (P*ptx*^*bro*^), respectively. Derivatives of these promoters and plasmids were created using a site-directed mutagenesis scheme that takes advantage of the type IIS restriction enzyme *Bsa*I, which cleaves asymmetrically to one side of its recognition sequence^40^ with modifications according to Chen et al.^24^. To measure the activity of P*ptx* at its native location in *B. bronchiseptica*, we used the promoter assay vector pSS4162^28^. This plasmid is a derivative of pSS3967 from which the 1.8 kb chromosomal fragment has been deleted. An *EcoR*I-*Sal*I PCR fragment, containing a longer upstream *ptx* promoter region, − 1170 to + 28 relative to the transcription start, encompassing nucleotides 5,197,773 to 5,198,965 from *B. bronchiseptica* RB50 genome sequence, was cloned into pSS4162 to create plasmid pQC1526 (P*ptx*^*bro*^). We cloned a similar fragment containing the *cyaA* promoter, from − 930 to + 37 relative to the transcriptional start, encompassing nucleotides 775,177 to 776,146 of the RB50 genome, between the *EcoR*I and *Sal*I sites in pSS4162 to generate plasmid pQC1598 (P*cya*^*bro*^) to be used as an active promoter control. Using the same procedures as for pSS3967-based plasmids, these two plasmids were integrated into the RB50 chromosome in order to assess *ptx* and *cya* promoter activity in situ.

To create an RB50 strain in which the P*ptx* promoter was that of *B. pertussis* we first constructed an allelic exchange plasmid containing P*ptx*^*bro*^ flanking sequences, with P*ptx*^*bro*^ deleted, in the vector pSS4661. Upstream and downstream flanking sequences encompassed nucleotides − 753 to − 167 and + 5 to + 586, respectively, relative to the P*ptx* start of transcription and were cloned as PCR fragments created using primers with *Bam*HI, *Bsa*I-*Sma*I, *Sma*I-*Bsa*I, and *Not*I sites at the upstream boundary, deletion points (*Sma*I site), and downstream boundary, respectively, between the *Not*I and *Bam*HI sites of pSS4661. The template for these PCR reactions was chromosomal DNA from RB50 and the primers used were Q1452 and Q1445 for the upstream flanking segment, and Q1453 and Q1446 for the downstream flanking segment. This resulted in the creation of pQC1540, which contained RB50 sequences from − 753 to + 586, relative to the P*ptx* transcriptional start site, with P*ptx*^*bro*^, from − 167 to + 4, replaced by the sequence 5′-gagacccccgggggtctc-3′ (*Bsa*I-*Sma*I-*Bsa*I). Digestion with *Bsa*I results in cleavage within the flanking RB50 sequences, thereby creating unique cohesive ends. A P*ptx*^*per*^ promoter fragment, was created by PCR with primers QC1454 and QC1455 which also contained *Bsa*I sites situated such that their cleavage with *Bsa*I would create cohesive ends that were complementary to those created by *Bsa*I cleavage of pQC1540. Ligation resulted in addition of the P*ptx* promoter, with no scar at the ligation points, to create pQC1541. This plasmid was then used to perform allelic exchange with RB50 as a recipient to create the strain QC2336 in which in which P*ptx*^*bro*^ was replaced by P*ptx*^*per*^. In a similar way pQC1540 itself was used in allelic exchange to create a strain in which P*ptx*^*bro*^ was deleted.

To create RB50 strains in which specific SNPs were introduced into P*ptx*^*bro*^, we first created an allelic exchange plasmid containing RB50 sequences from − 753 to + 586, relative to the start of transcription, in which *Bsa*I-based mutagenesis had been used to introduce the C^−13^T SNP in the − 10 region. The resulting plasmid pQC1542 (P*ptx*^*bro*^, C^−13^T) was then used as the basis for mutagenesis to introduce additional point mutations sequentially, thus creating the plasmids pQC1543 (P*ptx*^*bro*^, C^−13^T, C^−148^T), pQC1544 (P*ptx*^*bro*^, C^−13^T, C^−148^T, C^−126^T), pQC1545 (P*ptx*^*bro*^, C^−13^T, C^−148^T, C^−126^T, A^−70^G), and pQC1546 (P*ptx*^*bro*^, C^−13^T, C^−148^T, C^−126^T, A^−70^G, C^−39^T). To facilitate allelic exchange, we introduced the *aacCI* gene as a marker in the *Sma*I site of pQC1540 to generate pQC1561.

pSS4661 was created as follows. The previously reported allelic exchange plasmid pSS4245 comprises 8286 bp and contains a vegetative origin of replication (*oriV*) and ampicillin resistance gene (*bla*) from pBR322, an origin of transfer (*oriT*) from RK2, a segment of Tn5 containing *kan*, *ble*, and *str* genes encoding antibiotic resistance to kanamycin, bleomycin, and streptomycin, respectively, a module containing a gene encoding the intron-encoded homing endonuclease I-*Sce*I, driven by P*ptx* of *B. pertussis*, multiple transcriptional terminators upstream of P*ptx*, an I-*Sce*I cleavage site, and a multiple cloning site^41^. To create pSS4661 multiple deletions were introduced into pSS4245 to reduce the size of the segments containing *oriT* and *oriV* and to remove *bla* and *ble*. The resulting plasmid pSS4661 is 4663 bp in size but performs similarly to pSS4245.

DNA cloning procedures used *E. coli* DH5a as a transformation host. Plasmid constructs were verified by DNA sequencing (Macrogen, Rockville, MD).

### Conjugation and allelic exchange

To permit conjugation into *Bordetella*, pSS3967 and pSS4162 derivatives were transformed into the *E. coli* donor strain SM10 with selection on LB agar plus ampicillin and gentamicin. Recipient *Bordetella* strains *B. pertussis* BP536 and *B. bronchiseptica* RB50 were grown on BG agar supplemented with streptomycin. Conjugation was performed by swabbing the *E. coli* donor strain together with the *Bordetella* recipient strain on BG agar. After incubation at 37 °C for 3 h, bacteria were recovered by swabbing and re-swabbed onto BG agar plus gentamicin and streptomycin. Exconjugants in which the plasmid had integrated by homologous recombination arose after incubation at 37 °C for 2 days in the case of RB50 and 3 days for BP536.

To perform allelic exchange, all pSS4661-based plasmids were transformed into the *E. coli* donor strain S17.1 with selection for kanamycin resistance. Gentamicin was also added in the case of plasmid pQC1561. To alleviate P*ptx*^*per*^-driven I-*Sce*I expression from pSS4661 following transfer during the first conjugation step, BvgA phosphorylation by BvgS was abolished, and the Bvg^−^ mode was induced, by pre-growth of the recipient *B. bronchiseptica* RB50 or its derivatives on BG agar containing 50 mM MgSO_4_.

The first step in allelic exchange was initiated by performing conjugative transfer of the pSS4661-based allelic exchange plasmid derivative to the Bvg^−^ mode *B. bronchiseptica* recipient. Selection on BG agar containing kanamycin and streptomycin and 50 mM MgSO_4_ (gentamicin was also added in the case of pQC1561) led to isolation of exconjugants in which the plasmid had been integrated via a single crossover in the sequences flanking the mutation being introduced. Restreaking exconjugants on media lacking MgSO_4_ (including gentamicin in the case of pQC1561) caused the *Bordetella* recipient strain to grow in the Bvg^+^ mode in which phosphorylated BvgA activates expression of the homing endonuclease I-*Sce*I encoded on the pSS4661 vector thus causing cleavage of the I-*Sce*I restriction site. Repair of the resulting double stranded break occurs by recombination between the flanking cloned regions of homology such that either the wild-type sequence is reconstituted or the alteration of interest is incorporated. In the case of pQC1561, because P*ptx*^*bro*^ had been replaced with the selectable gentamicin resistance gene *aacCI,* maintenance of selection for gentamicin resistance throughout the procedure ensured that only the desired change was obtained in this last step. This led to the creation of strain QC2331 in which *aacCI* was inserted in place of P*ptx*^*bro*^. This strain was used as the recipient for allelic exchange with the plasmids pQC1542–pQC1546. In these cases, incorporation of the desired SNP-containing P*ptx*^*bro*^ alleles could be screened for in the final step by screening for loss of gentamicin resistance. In this way the following strains were created, QC2335 (RB50::ΔP*ptx*^*bro*^), QC2336 (RB50::P*ptx*^*per*^), QC2337 (RB50::P*ptx*^*bro*^, C^−13^T), QC2338 (RB50::P*ptx*^*bro*^, C^−13^T, C^−148^T), QC2339 (RB50::P*ptx*^*bro*^, C^−13^T, C^−148^T, C^−126^T), QC2340 (RB50::P*ptx*^*bro*^, C^−13^T, C^−148^T, C^−126^T, A^−70^G), and QC 2341 (RB50::P*ptx*^*bro*^, C^−13^T, C^−148^T, C^−126^T, A^−70^G, C^−39^T ).

### Luciferase activity assays

Imaging of luciferase activity of *Bordetella* strains was performed on an IVIS-50 instrument (Caliper Life Science) as has been described^24^. To accommodate the faster growth rate for *B. bronchiseptica*, RB50-derived strains were incubated for 24 h at 37 °C while BP536-derived strains were incubated for 48 h at 37 °C. Quantitative determination of luciferase activity was performed as has been described^24^. The average of at least four independent assays for each strain was reported.

### PT production in *B. bronchiseptica* RB50 strains

*B. bronchiseptica* RB50 strains carrying P*ptx* variants directing the expression of PT were grown on BG agar plus streptomycin (50 μg/mL) at 37 °C for 24 h. To allow PT production and secretion into the culture supernatant, two plates of cells for each strain was collected by swabbing and resuspended in 50 mL LB plus streptomycin (50 μg/mL), in a 250 mL flask, to a starting OD_600_ of approximately 0.5. After 24 h of incubation at 37 °C, with continuous shaking at 200 rpm, the cultures reached an OD_600_ of between 3 and 4, and were further adjusted to an OD_600_/mL of 3 by the addition of phosphate-buffered saline (PBS). Following centrifugation at 2600 × *g* for 30 min at 4 °C culture supernatants were collected and assessed for PT production by Western blot and PT CHO cell cytotoxicity assay, as described below.

### Western blot

To detect PT production by Western blot, to a 1.0 mL aliquot of culture supernatant, ¼ volume of 100% TCA was added, mixed, and the sample was held on ice for 1 h. Samples were centrifuged at 16,000 × g for 30 min at 4 °C and the supernatant was removed. The TCA-precipitated protein pellet was resuspended in 120 µL 1 × NuPAGE SDS Sample Buffer (Invitrogen) in the presence of 1 × NuPAGE Reducing Agent (Invitrogen) and denatured at 90 °C for 10 min. A 10 µL sample (equal to 0.25 OD_600_ units of cell culture supernatant) was fractionated on NuPAGE 4 ~ 12% Bis–Tris Gel (Invitrogen) in NuPAGE MES SDS Running Buffer (Invitrogen), alongside SeeBlue Plus2 Pre-Stained Standard (Invitrogen) and purified PT containing subunits S1–S5 (List Biological Laboratories, Inc.), loaded in the amounts indicated in Fig. 6. Separated proteins were transferred to PVDF filter paper (Invitrogen) according to the manufacturer’s instructions. Blocking of nonspecific antibody binding was performed in 5% non-fat milk for 1 h at room temperature. PT was detected by incubation of blots with monoclonal anti-PT subunit A (S1) antibody (3CX4-S1)^42^ at a dilution of 1:2,000 in 1% non-fat milk/PBS for 1 h, followed by washing three times for 10 min each with PBS plus 0.05% Tween-20, incubation with goat anti-mouse HRP-conjugated IgG (Santa Cruz Biotechnology) at 1:10,000 in 1% non-fat milk/PBS for 1 h, and washing three times for 10 min each with PBS plus 0.05% Tween-20. Antibody binding was detected by chemiluminescence using the ECL Plus Western Blotting Detection System (GE Healthcare), visualized using a G:Box Chemi-XT4 (SynGene). The magnitude of raw signals from specific bands were determined using GeneTools software (SynGene). For each sample on the Western blot, the amount of PT production (ng) was calculated based on the standard curve of PT established for each blot using a linear regression program in Prism 6 software, and converted to the amount of PT production (ng/OD_600_). PT production (ng/OD_600_) presented in Fig. 6A represent the average of duplicate samples independently prepared analyzed on a separate SDS-PAGE/Western blot. A representative Western blot was presented in Fig. 6B.

### PT CHO cell cytotoxicity assay

The PT CHO cell toxicity assay, based on the clustering effect induced by PT in cultured Chinese hamster ovary (CHO) cells^43^, was performed according to a standard protocol^44^. CHO cells were plated in a 96-well tissue culture plates in F-12 media, supplied with 1% FBS, 100 units of penicillin and 100 μg/mL streptomycin, and allowed to attach and grow overnight at 37 °C in the presence of 5% CO_2_. One of the two sets of culture supernatants assessed by Western blot were serially diluted (tenfold) and added to CHO cells at final dilutions of 10^−1^ to 10^−6^, in duplicate. After incubation for 24 h at 37 °C in the presence of 5% CO_2_, growth media were removed, and the cells were fixed with methanol and treated with giemsa stain to visualize cell morphology. The assay was scored as follows: 2 + , CHO cells tightly clumped; 1 + , CHO cells loosely clumped, some not clumped at all; 0, no apparent clumping. When the sum of two duplicates is 3 + or greater, the dilution is considered positive; if the sum of the duplicates is 2 + or less, the dilution is considered negative. Control purified PT (List Biological Laboratories, Inc.) at a starting concentration of 100 μg/mL was positive at a 10^−6^ dilution. Stock LB used as growth media for PT production was negative at a 10^−1^ dilution and was used as a negative control. The CHO cell assay on the same set of samples was repeated, and the scores obtained are presented in Fig. 6A.

### Sequence analysis of *Bordetella* P*ptx*

To collect sequences of P*ptx* from the different *Bordetella* species, a 1076 bp region spanning the *ptxA* coding region (810 bp) and its upstream sequence (266 bp) from the ancestor *B. bronchiseptica* lineage representative, *B. bronchiseptica* RB50 (genome sequence ID NC_002927.3, nucleotides 5,198,697 to 5,199,772), was used as a query to BLAST against 957 complete genomes of different *Bordetella* species in the BLAST database of NCBI (National Center for Biotechnology Information). By the MegaBLAST search, out of 957 complete genomes, a total of 848 hits (N = 848/957) containing sequences highly similar to the 1076 bp query were obtained, i.e., *B. bronchiseptica* (N = 17/23), *B. pertussis* (N = 745/748), *B. parapertussis* (N = 86/86), *B. holmesii* (N = 0/68), *B. hinzii* (N = 0/13), *B. petrii* (N = 0/1), *B. avium* (N = 0/6), *B. trematum* (N = 0/5), *B. pseudohinzii* (N = 0/2), *B. flabilis* (N = 0/1), *B. bronchialis* (N = 0/2), *B. ansorpii* (N = 0/2). The sequence fragments (~ 1 kb) obtained were further trimmed down to the *ptx* promoter region (~ 200 bp), equivalent to − 170 bp to + 9 relative to the P*ptx* + 1 of *B. bronchiseptica* RB50 (genome sequence ID NC_002927.3, nucleotides from 5,198,768 to 5,198,946). The collection (N = 848) of resulting regions for P*ptx* was organized into 14 groups (G1–G14), each containing a unique sequence of P*ptx* based on multiple sequence alignment using MUSCLE^45^. Identifying information for each of the 848 strains is provided in Table S1.

### Statistical analysis

At least four assays were used in the calculation of means, standard deviations, and statistical analyses of ordinary one-way ANOVA Dunnett’s multiple comparison tests carried out using Prism 6 software. Outcomes of the statistical analyses are presented using the symbols: ns, *P* > 0.05; **P ≤ 0.01; ****P ≤ 0.0001.

## Supplementary Information

Supplementary Information.
