# Supplementary material for: Four single-basepair mutations in the ptx promoter of Bordetella bronchiseptica are sufficient to activate the expression of pertussis toxin
Source: Sci Rep. 2021 Apr 30;11:9373. doi: 10.1038/s41598-021-88852-x (PMC8087692; doi:10.1038/s41598-021-88852-x)
Supplement: Supplementary file 1 — Supplementary Information. [file 41598_2021_88852_MOESM1_ESM.pdf]

Four single-basepair mutations in the *ptx* promoter of *Bordetella bronchiseptica* are sufficient to activate the expression of pertussis toxin

Qing Chen[1]*, Mary C. Gray[2], Erik Hewlett[2] and Scott Stibitz[1]

Fig. S1

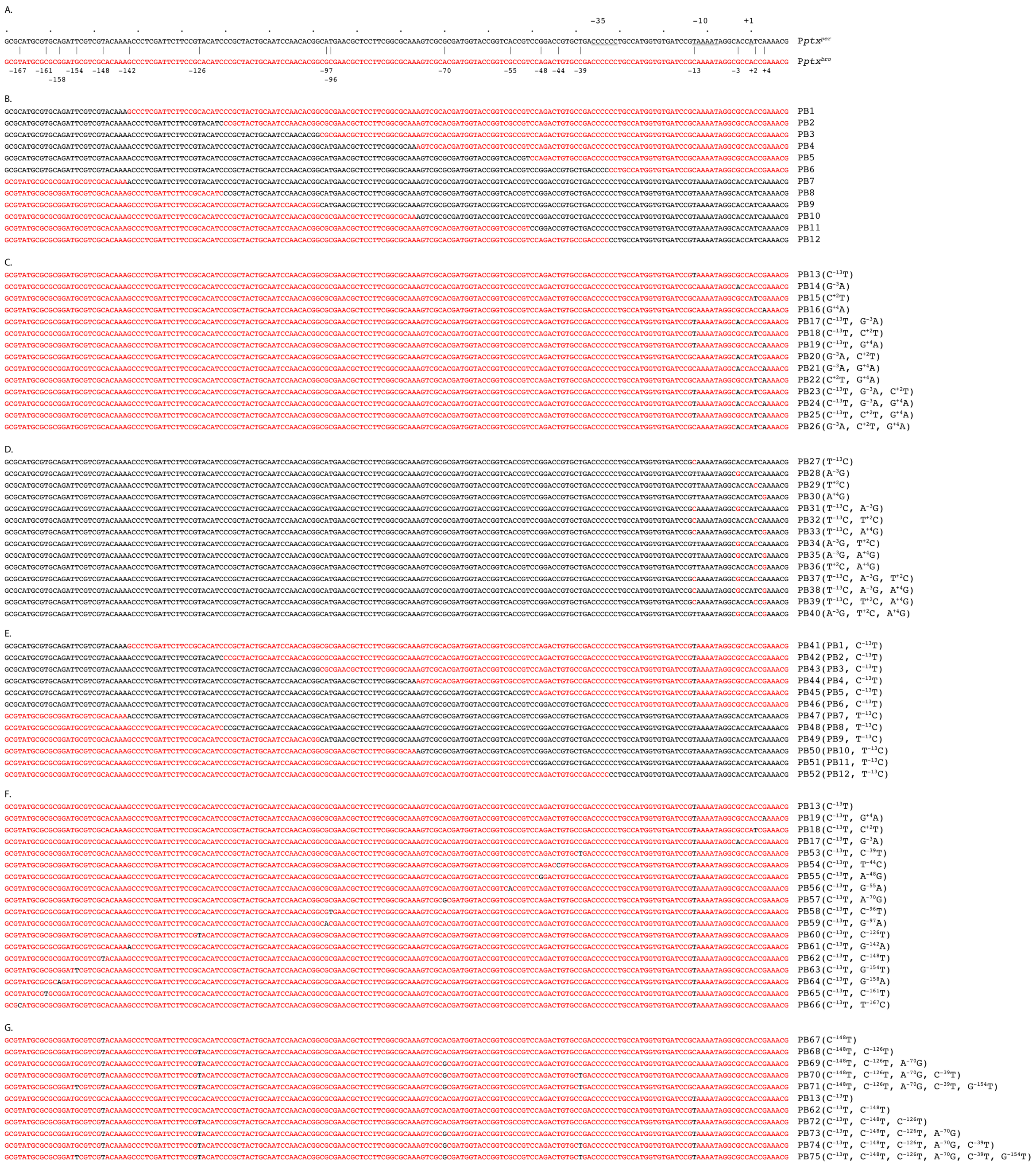

Supplementary Fig. S1. Sequences of hybrid ptx promoters. (A) The sequence of P*ptx*^{per} is shown above in black, and that of P*ptx*^{bro} below in red. The SNPs between two promoters are indicated by the vertical dashes with their positions numbered relative to the +1 transcriptional start. (B) - (G). The sequences of hybrid promoters, PB1 ~ PB75 are provided with sequences of P*ptx*^{per} origin in black and those of P*ptx*^{bro} origin in red. Specific SNPs present in PB13-PB75 are also provided in parentheses.

Supplementry Table S1. Bordetella genomes used for P*ptx* sequence analysis

Geonome group ID, P*ptx* sequence and strain	Sequence ID	Genome length (bp)
G1_Bb_RB50		
GCGTATGCGCGCGGATGCGTCGCACAAAGCCCTCGATTCTTCCGCACATCCCGC<br>TACTGCAATCCAACACGGCGCGAACGCTCCTTCGGCGCAAAGTCGCACGATGGT<br>ACCGGTCGCCGTCCAGACTGTGCCGACCCCCCTGCCATGGTGTGATCCGCAAAA<br>TAGGCGCCACCGAAACG		
Bordetella bronchiseptica strain RB50	NC_002927.3	5339179
Bordetella bronchiseptica strain KVNON-570	NZ_CP049918.1	5211648
Bordetella bronchiseptica strain FDAARGOS_176	NZ_CP014013.1	5339230
G2_Bb_NCTC8344		
GCGTATGCGCGCGGATGCGTCGCACAAAGCCCTCGATTCTTCCGCACATCCCGC<br>TACTGCAATCCAACACGGCGCGAACGCTCCTTCGGCGCAAAGTCGCACGATGGT<br>ACCGGTCACCGTCCAGACTGTGCCGACCCCCCTGCCATGGTGTGATCCGCAAAA<br>TAGGCGCCACCGAAACG		
Bordetella bronchiseptica strain NCTC8344	NZ_LR134480.1	5207445
Bordetella bronchiseptica strain E001	NZ_CP020650.1	5209087
Bordetella bronchiseptica strain NCTC10543	NZ_LR134326.1	5199761
G3_Bb_KM22		
GCGTATGCGCGCGGATGCGTCGCACAAAGCCCTCAATCCTTCCGCACATCCCGC<br>TACTGCAATCCAACACGGCGCAAACACCCCTTCGGCGCAAAGTCGCACGATGGT<br>ACCGGTCGCCGTCCGGACCGTGCCGACCCCCCTGCCATGGTGTGATCCGCAAAA<br>TAGGCGCCACAGAAACG		
Bordetella bronchiseptica strain KM22	NZ_CP022962.2	5205646
Bordetella bronchiseptica strain S798	NZ_AP014582.1	5191712
G4_Bb_D448		
GCGTATGCGCGCGGATGCGTCGCACAAAGCCCTCGATCCTTCCGCACATCCCGC<br>TACTGCAATCCAACACGGCGCGAACGCCCCTTCGGCGCAAAGTCGCACGATGGT<br>ACCGGTCGCCGTCCGGACCGTGCCGACCCCCCTGCCATGGTGTGATCCGCACAA<br>TAGGCACCACGGAAACG		
Bordetella bronchiseptica strain D448	NZ_CP024171.1	5206932
Bordetella bronchiseptica strain E016	NZ_CP019934.1	5167199
Bordetella bronchiseptica strain D755	NZ_CP020651.1	5200001
Bordetella bronchiseptica strain D973	NZ_CP020819.1	5200288
G5_Bb_E010		
GCGTATGCGCGCGGATGCGCGCGGATGCGTCGCACAAAGCCCTCGATCCTTCCG<br>CACATCCCGCTACTGCAATCCAACACGGCGCGAACGCCCCTTCGGCGCAAAGTC<br>GCACGATGGTACCGGTCGCCGTCCGGACCGTGCCGACCCCCCTGCCATGGTGTG<br>ATCCGC-ACAATAGGCACCACGGAAACG		
Bordetella bronchiseptica strain E010	NZ_CP020649.1	5204290
Bordetella bronchiseptica strain D987	NZ_CP024173.1	5193581
Bordetella bronchiseptica strain 253	NC_019382.1	5264383
G6_Bb_I124		
GGGCCCGTGCGCGGACGCGTCGCACGAAACCCCCGATTCTTCCGCACATTCCGC<br>TGCTGCAATGCCACGTGGCGTCAACGCCGCTTCGTCGCAAGGCGTCTCGATGGT<br>ACCGGCCGCAGTCCGGATTCTGCCGACCCTCGCGCCATGAGGCCATCCGCAAAA<br>ATATGCGCCTTGGAAACG		
Bordetella bronchiseptica strain I124	NZ_CP025069.1	5081368
Bordetella bronchiseptica strain I328	NZ_CP016431.1	5077489
G7_Bpp_Bpp5		
GCGTATGCGCGCGGATGCGTCGCACAAAACCCTCGATCCTTCCGCACATCCCGC<br>TACTGCAATCCAACACGGCGCGAACGCTCCTTCGGCGCAAAGTCGCACGATGGT<br>ACCGGTCGCCGTCCGGACCGTGCCGACCCCCCTGCCATGGTGTGATCCGCACAA<br>TAGGCGCTACCGAAACG		
Bordetella parapertussis strain Bpp5	NC_018828.1	4887379
G8_Bpp_12822		
GCGTATGCGCGCGGATGCGCGCGGATGCGTCGCACAAAGCCCTCAATCCTTCCG<br>CACATCCCGCTACTGTAATCCAACACGGCGCAAACGCCCCTTCGGCGCAAAGTC<br>GCACGATGGTACCGGTCGCCGTCCGGACCGTGCCGACCCCCCTGCCATGGTGTG<br>ATCCGC-AAAATAGGCGCCACAGAAACG		
Bordetella parapertussis strain 12822	NC_002928.3	4773551
Bordetella parapertussis strain B149	NZ_CP025071.1	4773820
Bordetella parapertussis strain B271	NZ_CP025067.1	4773897
Bordetella parapertussis strain A005	NZ_CP025070.1	4773835
Bordetella parapertussis strain F585	NZ_CP024174.1	4773857
Bordetella parapertussis strain B144	NZ_CP025068.1	4773899
Bordetella parapertussis strain B160	NZ_CP025072.1	4775493
Bordetella parapertussis strain H555	NZ_CP043144.1	4773919
Bordetella parapertussis strain H581	NZ_CP043139.1	4773334
Bordetella parapertussis strain I267	NZ_CP043117.1	4773892
Bordetella parapertussis strain I301	NZ_CP043115.1	4773985
Bordetella parapertussis strain I458	NZ_CP043110.1	4773807
Bordetella parapertussis strain J677	NZ_CP052851.1	4773922
Bordetella parapertussis strain J778	NZ_CP052850.1	4771868
Bordetella parapertussis strain J069	NZ_CP043107.1	4773916
Bordetella parapertussis strain J156	NZ_CP043104.1	4773906
Bordetella parapertussis strain J217	NZ_CP043099.1	4773518
Bordetella parapertussis strain J219	NZ_CP043097.1	4773925
Bordetella parapertussis strain H101	NZ_CP043164.1	4773865
Bordetella parapertussis strain J279	NZ_CP043093.1	4773927
Bordetella parapertussis strain J280	NZ_CP043092.1	4773920
Bordetella parapertussis strain J289	NZ_CP043087.1	4773902
Bordetella parapertussis strain J297	NZ_CP043082.1	4773920
Bordetella parapertussis strain J328	NZ_CP043076.1	4773924
Bordetella parapertussis strain J599	NZ_CP043072.1	4773877
Bordetella parapertussis strain J711	NZ_CP043067.1	4773907
Bordetella parapertussis strain H100	NZ_CP043165.1	4773923
Bordetella parapertussis strain J859	NZ_CP043061.1	4773921
Bordetella parapertussis strain H102	NZ_CP043163.1	4773913
Bordetella parapertussis strain I143	NZ_CP043119.1	4773867
Bordetella parapertussis strain H242	NZ_CP043159.1	4773905
Bordetella parapertussis strain I457	NZ_CP043111.1	4773886
Bordetella parapertussis strain I768	NZ_CP043109.1	4773834

Bordetella parapertussis strain J278	NZ_CP043094.1	4773927
Bordetella parapertussis strain J284	NZ_CP043089.1	4773857
Bordetella parapertussis strain J287	NZ_CP043088.1	4773909
Bordetella parapertussis strain J292	NZ_CP043084.1	4773876
Bordetella parapertussis strain J293	NZ_CP043083.1	4773805
Bordetella parapertussis strain J324	NZ_CP043079.1	4773886
Bordetella parapertussis strain J326	NZ_CP043078.1	4773926
Bordetella parapertussis strain J593	NZ_CP043073.1	4773920
Bordetella parapertussis strain H104	NZ_CP043162.1	4773906
Bordetella parapertussis strain J835	NZ_CP043062.1	4773244
Bordetella parapertussis strain H105	NZ_CP043161.1	4773835
Bordetella parapertussis strain I325	NZ_CP043113.1	4773929
Bordetella parapertussis strain J281	NZ_CP043091.1	4773951
Bordetella parapertussis strain J290	NZ_CP043086.1	4773922
Bordetella parapertussis strain H554	NZ_CP043145.1	4773847
Bordetella parapertussis strain J307	NZ_CP043081.1	4773851
Bordetella parapertussis strain J327	NZ_CP043077.1	4773927
Bordetella parapertussis strain I288	NZ_CP043116.1	4773916
Bordetella parapertussis strain I308	NZ_CP043114.1	4773982
Bordetella parapertussis strain I326	NZ_CP043112.1	4773923
Bordetella parapertussis strain I139	NZ_CP043120.1	4773878
Bordetella parapertussis strain I962	NZ_CP043108.1	4773906
Bordetella parapertussis strain J116	NZ_CP043105.1	4773885
Bordetella parapertussis strain J602	NZ_CP043071.1	4773917
Bordetella parapertussis strain J158	NZ_CP043103.1	4773912
Bordetella parapertussis strain J166	NZ_CP043102.1	4773877
Bordetella parapertussis strain J221	NZ_CP043095.1	4773903
Bordetella parapertussis strain J282	NZ_CP043090.1	4773913
Bordetella parapertussis strain J291	NZ_CP043085.1	4773925
Bordetella parapertussis strain H314	NZ_CP043155.1	4773862
Bordetella parapertussis strain J396	NZ_CP043075.1	4773877
Bordetella parapertussis strain J456	NZ_CP043074.1	4773909
Bordetella parapertussis strain H580	NZ_CP043140.1	4773924
Bordetella parapertussis strain J611	NZ_CP043070.1	4773915
Bordetella parapertussis strain J676	NZ_CP043069.1	4773916
Bordetella parapertussis strain J786	NZ_CP043064.1	4773962
Bordetella parapertussis strain I133	NZ_CP043121.1	4773929
Bordetella parapertussis strain J792	NZ_CP043063.1	4773959
Bordetella parapertussis strain Bpp01	NZ_AP019378.1	4787082
Bordetella parapertussis strain E843	NZ_CP019931.1	4773898
Bordetella parapertussis strain E762	NZ_CP019932.1	4773891
Bordetella parapertussis strain H889	NZ_CP018897.1	4773902
Bordetella parapertussis strain D577	NZ_CP020652.1	4773898
Bordetella parapertussis strain E738	NZ_CP020648.1	4773977
Bordetella parapertussis strain I440	NZ_CP020646.1	4773906
Bordetella parapertussis strain B337	NZ_CP020654.1	4773876
Bordetella parapertussis strain A747	NZ_CP020655.1	4773897
Bordetella parapertussis strain H904	NZ_CP016342.1	4773708
Bordetella parapertussis strain J220	CP043096.1	4773756
Bordetella parapertussis strain NCTC10520	NZ_LS483424.1	4775387
Bordetella parapertussis strain NCTC10522	NZ_LR590480.1	4775440
Bordetella parapertussis strain NCTC10524	NZ_LR134329.1	4774438
G9_Bp_18323		
GCGTATGCGTGCGGATGCGTCGTACAAAACCCTCGATTATTCCGTACATCCCGC<br>TACTGCAATCCAACACGGCGTAAACGCTCCTTCGGCGCAAAGTCGCGCGATGGT<br>ACCGGTCACCGTCCGGACCGTGCTGACCCCCCTGCCATGGTGTGATCCGTAAAA<br>TAGGCACCACCGAAACG		
Bordetella pertussis strain 18323	NC_018518.1	4043846
Bordetella pertussis strain C927	NZ_CP016339.1	4095467
G10_Bp_137		
GCGTATGCGTGCAGATTCGTCGTACAAAACCCTCGATTCTTCCGTACATCCCGC<br>TACTGCAATCCAACACGGCATGAACGCTCCTTCGGCGCAAAGTCGCGCGATGGT<br>ACCGGTCACCGTCCGGACCGTGCTGACCCCCCTGCCATGGTGTGATCCGTAAAA<br>TAGGCACCATCAAAACG		
Bordetella pertussis strain 137	NZ_CP010323.1	4134593
Bordetella pertussis strain B199	NZ_CP022361.1	4314502
Bordetella pertussis strain 509	NZ_CP017403.1	4140370
Bordetella pertussis strain B203	NZ_CP012128.1	4134643
Bordetella pertussis strain ATCC	CP046981.1	4269194
G11_Bp_D420		
GCGCATGCGTGCAGATTCGTCGTACAAAACCCTCGATTCTTCCGTACATCCCGC<br>TACTGCAATCCAACACGGCATGAACGCTCCTTCGGCGCAAAGTCGCGCGATAGT<br>ACCGGTCACCGTCCGGACCGTGCTGACCCCCCTGCCATGGTGTGATCCGTAAAA<br>TAGGCACCATCAAAACG		
Bordetella pertussis strain D420	NZ_LN849008.1	4106389
Bordetella pertussis strain FDAARGOS_1367	NZ_CP070069.1	4105604
Bordetella pertussis strain BP6242	NZ_LS483259.1	4105356
Bordetella pertussis strain BP82	NZ_LS483252.1	4104282
Bordetella pertussis strain BP6260	NZ_LS483256.1	4110570
Bordetella pertussis strain BP46	NZ_LS483251.1	4104308
Bordetella pertussis strain BP155	NZ_LS483253.1	4109117
Bordetella pertussis strain BP6384	NZ_LS483257.1	4105215
Bordetella pertussis strain BPK10	NZ_LS483255.1	4104707
Bordetella pertussis strain BP318	NZ_LS483260.1	4113685
Bordetella pertussis strain BP312	NZ_LS483258.1	4105753
Bordetella pertussis strainB1917	NZ_CP009751.1	4102186
Bordetella pertussis strain E153	NZ_CP025359.1	4108487
Bordetella pertussis strain H811	NZ_CP025361.1	4110592
Bordetella pertussis strain H877	NZ_CP025382.1	4105345
Bordetella pertussis strain E198	NZ_CP025385.1	4101116
Bordetella pertussis strain I896	NZ_CP025381.1	4101872
Bordetella pertussis strain E365	NZ_CP025387.1	4103253
Bordetella pertussis strain I598	NZ_CP025380.1	4107721
Bordetella pertussis strain H813	NZ_CP025351.1	4106497

Bordetella pertussis strain D236	NZ_CP025530.1	4285328
Bordetella pertussis strain I705	NZ_CP025524.1	4105347
Bordetella pertussis strain I420	NZ_CP025525.1	4111618
Bordetella pertussis strain J139	NZ_CP025527.1	4247121
Bordetella pertussis strain I955	NZ_CP025531.1	4107611
Bordetella pertussis strain I462	NZ_CP025376.1	4107545
Bordetella pertussis strain D665	NZ_CP025526.1	4145812
Bordetella pertussis strain F569	NZ_CP025523.1	4103262
Bordetella pertussis strain I945	NZ_CP025384.1	4107408
Bordetella pertussis strain H624	NZ_CP025529.1	4121896
Bordetella pertussis strain D869	NZ_CP025528.1	4104044
Bordetella pertussis strain E191	NZ_CP025478.1	4104745
Bordetella pertussis strain J184	NZ_CP025383.1	4104293
Bordetella pertussis strain G102	NZ_CP025388.1	4110608
Bordetella pertussis strain C756	NZ_CP025368.1	4104277
Bordetella pertussis strain I859	NZ_CP025477.1	4107433
Bordetella pertussis strain I892	NZ_CP025479.1	4101882
Bordetella pertussis strain E087	NZ_CP025480.1	4101146
Bordetella pertussis strain E140	NZ_CP025354.1	4103234
Bordetella pertussis strain I692	NZ_CP025378.1	4105345
Bordetella pertussis strain I372	NZ_CP025372.1	4106744
Bordetella pertussis strain I120	NZ_CP025370.1	4102199
Bordetella pertussis strain I623	NZ_CP025386.1	4105302
Bordetella pertussis strain I439	NZ_CP025375.1	4106374
Bordetella pertussis strain H814	NZ_CP025374.1	4105351
Bordetella pertussis strain I223	NZ_CP025369.1	4102613
Bordetella pertussis strain H709	NZ_CP025364.1	4103241
Bordetella pertussis strain I188	NZ_CP025379.1	4100921
Bordetella pertussis strain H697	NZ_CP025365.1	4104135
Bordetella pertussis strain H902	NZ_CP025363.1	4105338
Bordetella pertussis strain H642	NZ_CP025360.1	4085512
Bordetella pertussis strain I323	NZ_CP025377.1	4106368
Bordetella pertussis strain H541	NZ_CP025373.1	4104306
Bordetella pertussis strain H640	NZ_CP025371.1	4088701
Bordetella pertussis strain H778	NZ_CP025362.1	4106328
Bordetella pertussis strain H920	NZ_CP025352.1	4106670
Bordetella pertussis strain H034	NZ_CP025356.1	4105484
Bordetella pertussis strain D322	NZ_CP025358.1	4104278
Bordetella pertussis strain J024	NZ_CP025353.1	4106548
Bordetella pertussis strain F578	NZ_CP025357.1	4097016
Bordetella pertussis strain D919	NZ_CP025355.1	4106395
Bordetella pertussis strain H782	NZ_CP018035.1	4108455
Bordetella pertussis strain J384	NZ_CP025073.1	4110543
Bordetella pertussis strain J077	NZ_CP025344.1	4108444
Bordetella pertussis strain J234	NZ_CP025348.1	4107445
Bordetella pertussis strain C569	NZ_CP025347.1	4105353
Bordetella pertussis strain F580	NZ_CP025342.1	4104318
Bordetella pertussis strain H742	NZ_CP025346.1	4104296
Bordetella pertussis strain J185	NZ_CP025343.1	4106374
Bordetella pertussis strain I958	NZ_CP025350.1	4108479
Bordetella pertussis strain H672	NZ_CP025349.1	4107426
Bordetella pertussis strain C871	NZ_CP025345.1	4100074
Bordetella pertussis strain J377	NZ_CP014211.1	4106512
Bordetella pertussis strain J358	NZ_CP014212.1	4110595
Bordetella pertussis strain J262	NZ_CP026452.1	4110580
Bordetella pertussis strain J369	NZ_CP026669.1	4103232
Bordetella pertussis strain J390	NZ_CP026664.1	4106379
Bordetella pertussis strain J525	NZ_CP026624.1	4107260
Bordetella pertussis strain J522	NZ_CP026627.1	4106373
Bordetella pertussis strain J529	NZ_CP026621.1	4105348
Bordetella pertussis strain J656	NZ_CP026900.1	4108772
Bordetella pertussis strain J614	NZ_CP026904.1	4104254
Bordetella pertussis strain J693	NZ_CP026889.1	4105318
Bordetella pertussis strain J700	NZ_CP026884.1	4107386
Bordetella pertussis strain J391	NZ_CP026469.1	4107431
Bordetella pertussis strain J392	NZ_CP026468.1	4107431
Bordetella pertussis strain J255	NZ_CP026458.1	4110576
Bordetella pertussis strain J268	NZ_CP026447.1	4108457
Bordetella pertussis strain J252	NZ_CP026461.1	4110577
Bordetella pertussis strain J253	NZ_CP026460.1	4108481
Bordetella pertussis strain J259	NZ_CP026454.1	4110580
Bordetella pertussis strain J267	NZ_CP026448.1	4110579
Bordetella pertussis strain J323	NZ_CP026440.1	4106376
Bordetella pertussis strain J272	NZ_CP026444.1	4110578
Bordetella pertussis strain J336	NZ_CP026436.1	4107434
Bordetella pertussis strain J364	NZ_CP026430.1	4095487
Bordetella pertussis strain J375	NZ_CP026666.1	4106495
Bordetella pertussis strain J379	NZ_CP026665.1	4106636
Bordetella pertussis strain J386	NZ_CP026629.1	4106400
Bordetella pertussis strain J424	NZ_CP026628.1	4109501
Bordetella pertussis strain J374	NZ_CP026631.1	4111643
Bordetella pertussis strain J378	NZ_CP026630.1	4106635

Bordetella pertussis strain J528	NZ_CP026622.1	4104297
Bordetella pertussis strain J616	NZ_CP026903.1	4104267
Bordetella pertussis strain J606	NZ_CP026905.1	4112660
Bordetella pertussis strain J692	NZ_CP026890.1	4105332
Bordetella pertussis strain J699	NZ_CP026885.1	4107132
Bordetella pertussis strain J376	NZ_CP026470.1	4107552
Bordetella pertussis strain J250	NZ_CP026463.1	4110579
Bordetella pertussis strain J251	NZ_CP026462.1	4110582
Bordetella pertussis strain J257	NZ_CP026456.1	4110581
Bordetella pertussis strain J258	NZ_CP026455.1	4110584
Bordetella pertussis strain J264	NZ_CP026450.1	4110583
Bordetella pertussis strain J266	NZ_CP026449.1	4110579
Bordetella pertussis strain J274	NZ_CP026443.1	4110548
Bordetella pertussis strain J270	NZ_CP026445.1	4110582
Bordetella pertussis strain J334	NZ_CP026437.1	4110578
Bordetella pertussis strain I182	NZ_CP026996.1	4112703
Bordetella pertussis strain J247	NZ_CP026988.1	4109425
Bordetella pertussis strain J586	NZ_CP026911.1	4106373
Bordetella pertussis strain J092	NZ_CP026922.1	4113725
Bordetella pertussis strain J549	NZ_CP026915.1	4106572
Bordetella pertussis strain J567	NZ_CP026914.1	4106621
Bordetella pertussis strain J589	NZ_CP026909.1	4105313
Bordetella pertussis strain J662	NZ_CP026899.1	4104253
Bordetella pertussis strain J683	NZ_CP026894.1	4110594
Bordetella pertussis strain J372	NZ_CP026668.1	4105473
Bordetella pertussis strain J526	NZ_CP026663.1	4105346
Bordetella pertussis strain J362	NZ_CP026633.1	4099525
Bordetella pertussis strain J523	NZ_CP026626.1	4108503
Bordetella pertussis strain J632	NZ_CP026620.1	4107723
Bordetella pertussis strain J603	NZ_CP026908.1	4112699
Bordetella pertussis strain J617	NZ_CP026902.1	4104279
Bordetella pertussis strain J628	NZ_CP026901.1	4107705
Bordetella pertussis strain J695	NZ_CP026888.1	4105341
Bordetella pertussis strain J703	NZ_CP026882.1	4105128
Bordetella pertussis strain J367	NZ_CP026472.1	4108483
Bordetella pertussis strain J401	NZ_CP026467.1	4113744
Bordetella pertussis strain J254	NZ_CP026459.1	4110577
Bordetella pertussis strain J260	NZ_CP026453.1	4110577
Bordetella pertussis strain J085	NZ_CP026997.1	4296902
Bordetella pertussis strain J246	NZ_CP026989.1	4103095
Bordetella pertussis strain J233	NZ_CP026917.1	4104389
Bordetella pertussis strain J546	NZ_CP026916.1	4106441
Bordetella pertussis strain J588	NZ_CP026910.1	4106460
Bordetella pertussis strain J680	NZ_CP026896.1	4110583
Bordetella pertussis strain J682	NZ_CP026895.1	4110562
Bordetella pertussis strain J066	NZ_CP026998.1	4107429
Bordetella pertussis strain J205	NZ_CP026990.1	4106403
Bordetella pertussis strain J129	NZ_CP026920.1	4107579
Bordetella pertussis strain J132	NZ_CP026918.1	4087073
Bordetella pertussis strain J582	NZ_CP026912.1	4106349
Bordetella pertussis strain J691	NZ_CP026891.1	4105328
Bordetella pertussis strain J702	NZ_CP026883.1	4105107
Bordetella pertussis strain J269	NZ_CP026446.1	4108484
Bordetella pertussis strain J332	NZ_CP026438.1	4109535
Bordetella pertussis strain J275	NZ_CP026442.1	4108430
Bordetella pertussis strain J354	NZ_CP026435.1	4106370
Bordetella pertussis strain J366	NZ_CP026429.1	4110583
Bordetella pertussis strain J171	NZ_CP026992.1	4104386
Bordetella pertussis strain J204	NZ_CP026991.1	4113707
Bordetella pertussis strain J303	NZ_CP026987.1	4108452
Bordetella pertussis strain J118	NZ_CP026921.1	4103077
Bordetella pertussis strain J130	NZ_CP026919.1	4103048
Bordetella pertussis strain J571	NZ_CP026913.1	4106638
Bordetella pertussis strain J667	NZ_CP026898.1	4108749
Bordetella pertussis strain J679	NZ_CP026897.1	4110580
Bordetella pertussis strain J687	NZ_CP026893.1	4106343
Bordetella pertussis strain J373	NZ_CP026667.1	4110597
Bordetella pertussis strain J368	NZ_CP026632.1	4110592
Bordetella pertussis strain J524	NZ_CP026625.1	4107246
Bordetella pertussis strain J527	NZ_CP026623.1	4105308
Bordetella pertussis strain J650	NZ_CP026619.1	4107707
Bordetella pertussis strain J673	NZ_CP026618.1	4107704
Bordetella pertussis strain J604	NZ_CP026907.1	4111650
Bordetella pertussis strain J605	NZ_CP026906.1	4112682
Bordetella pertussis strain J690	NZ_CP026892.1	4105314
Bordetella pertussis strain J698	NZ_CP026886.1	4107367
Bordetella pertussis strain J370	NZ_CP026471.1	4107544
Bordetella pertussis strain J402	NZ_CP026466.1	4107550
Bordetella pertussis strain J249	NZ_CP026464.1	4110576
Bordetella pertussis strain J256	NZ_CP026457.1	4110580
Bordetella pertussis strain J263	NZ_CP026451.1	4110580
Bordetella pertussis strain J357	NZ_CP026434.1	4107421

Bordetella pertussis strain J393	NZ_CP026428.1	4106382
Bordetella pertussis strain J322	NZ_CP026441.1	4106368
Bordetella pertussis strain J330	NZ_CP026439.1	4107415
Bordetella pertussis strain J359	NZ_CP026433.1	4107267
Bordetella pertussis strain J360	NZ_CP026432.1	4110459
Bordetella pertussis strain J209	NZ_CP017120.1	4107500
Bordetella pertussis strain D717	NZ_CP016964.1	4102255
Bordetella pertussis strain D422	NZ_CP016959.1	4107441
Bordetella pertussis strain E537	NZ_CP016958.1	4110590
Bordetella pertussis strain J172	NZ_CP017121.1	4107375
Bordetella pertussis strain E541	NZ_CP016966.1	4105336
Bordetella pertussis strain D735	NZ_CP016960.1	4106298
Bordetella pertussis strain J160	NZ_CP017119.1	4112627
Bordetella pertussis strain D925	NZ_CP016968.1	4106392
Bordetella pertussis strain D799	NZ_CP016963.1	4103196
Bordetella pertussis strain E898	NZ_CP016962.1	4103239
Bordetella pertussis strain J153	NZ_CP017123.1	4106365
Bordetella pertussis strain J154	NZ_CP017122.1	4106367
Bordetella pertussis strain E025	NZ_CP016967.1	4101132
Bordetella pertussis strain C934	NZ_CP016961.1	4104297
Bordetella pertussis strain J199	NZ_CP011245.1	4109686
Bordetella pertussis strain J014	NZ_CP012135.1	4107409
Bordetella pertussis strain I646	NZ_CP010263.1	4105348
Bordetella pertussis strain H564	NZ_CP010249.1	4109554
Bordetella pertussis strain I476	NZ_CP010254.1	4105331
Bordetella pertussis strain I656	NZ_CP010264.1	4101762
Bordetella pertussis strain H622	NZ_CP010847.1	4107431
Bordetella pertussis strain H542	NZ_CP010843.1	4103349
Bordetella pertussis strain I518	NZ_CP010259.1	4105350
Bordetella pertussis strain I521	NZ_CP010260.1	4104288
Bordetella pertussis strain I480	NZ_CP010255.1	4106388
Bordetella pertussis strain H788	NZ_CP010250.1	4106823
Bordetella pertussis strain I472	NZ_CP010253.1	4111652
Bordetella pertussis strain H489	NZ_CP010842.1	4106364
Bordetella pertussis strain H379	NZ_CP010840.1	4105330
Bordetella pertussis strain I496	NZ_CP010257.1	4096713
Bordetella pertussis strain I469	NZ_CP010252.1	4111656
Bordetella pertussis strain I539	NZ_CP010262.1	4106666
Bordetella pertussis strain I538	NZ_CP010261.1	4107716
Bordetella pertussis strain I468	NZ_CP010251.1	4106401
Bordetella pertussis strain H380	NZ_CP010841.1	4104286
Bordetella pertussis strain H561	NZ_CP010845.1	4073006
Bordetella pertussis strain H559	NZ_CP010844.1	4106490
Bordetella pertussis strain I483	NZ_CP010256.1	4107437
Bordetella pertussis strain H563	NZ_CP010846.1	4106394
Bordetella pertussis strain I498	NZ_CP010258.1	4106502
Bordetella pertussis strain I707	NZ_CP010266.1	4107557
Bordetella pertussis strain I669	NZ_CP010265.1	4103417
Bordetella pertussis strain H627	NZ_CP010962.1	4107408
Bordetella pertussis strain H374	NZ_CP010838.1	4107437
Bordetella pertussis strain H378	NZ_CP010839.1	4107534
Bordetella pertussis strain NCTC13667	NZ_LT906484.1	4122636
Bordetella pertussis strain NCTC13666	NZ_LT906471.1	4108502
Bordetella pertussis strain J096	NZ_CP013951.1	4105309
Bordetella pertussis strain J208	NZ_CP013904.1	4108471
Bordetella pertussis strain J161	NZ_CP013898.1	4106377
Bordetella pertussis strain J120	NZ_CP013890.1	4109522
Bordetella pertussis strain J097	NZ_CP013885.1	4107418
Bordetella pertussis strain I093	NZ_CP013883.1	4101532
Bordetella pertussis strain H540	NZ_CP013880.1	4105291
Bordetella pertussis strain H348	NZ_CP013877.1	4106382
Bordetella pertussis strain F778	NZ_CP013872.1	4090785
Bordetella pertussis strain J300	NZ_CP013864.1	4105330
Bordetella pertussis strain H730	NZ_CP013086.1	4102201
Bordetella pertussis strain H698	NZ_CP013084.1	4104135
Bordetella pertussis strain E602	NZ_CP013081.1	4108490
Bordetella pertussis strain C734	NZ_CP013078.1	4102201
Bordetella pertussis strain J100	NZ_CP011766.1	4109710
Bordetella pertussis strain J090	NZ_CP011763.1	4106346
Bordetella pertussis strain J068	NZ_CP011759.1	4107434
Bordetella pertussis strain J027	NZ_CP011756.1	4109543
Bordetella pertussis strain J206	NZ_CP013903.1	4110604
Bordetella pertussis strain J159	NZ_CP013897.1	4107438
Bordetella pertussis strain J149	NZ_CP013894.1	4105638
Bordetella pertussis strain J124	NZ_CP013893.1	4107607
Bordetella pertussis strain J121	NZ_CP013891.1	4103084
Bordetella pertussis strain J104	NZ_CP013888.1	4108480
Bordetella pertussis strain J093	NZ_CP013884.1	4113750
Bordetella pertussis strain H636	NZ_CP013881.1	4105336
Bordetella pertussis strain G965	NZ_CP013876.1	4105340
Bordetella pertussis strain E368	NZ_CP013869.1	4104277
Bordetella pertussis strain J305	NZ_CP013865.1	4111657

Bordetella pertussis strain J296	NZ_CP013863.1	4107428
Bordetella pertussis strain J201	NZ_CP013095.1	4103283
Bordetella pertussis strain H706	NZ_CP013085.1	4105094
Bordetella pertussis strain E194	NZ_CP013080.1	4103209
Bordetella pertussis strain C549	NZ_CP013077.1	4106401
Bordetella pertussis strain J115	NZ_CP011768.1	4106397
Bordetella pertussis strain J098	NZ_CP011765.1	4107474
Bordetella pertussis strain J073	NZ_CP011761.1	4112724
Bordetella pertussis strain J030	NZ_CP011757.1	4105288
Bordetella pertussis strain J021	NZ_CP011754.1	4106673
Bordetella pertussis strain J019	NZ_CP011753.1	4107558
Bordetella pertussis strain I386	NZ_CP011738.1	4104275
Bordetella pertussis strain I350	NZ_CP011733.1	4107435
Bordetella pertussis strain I273	NZ_CP011728.1	4110433
Bordetella pertussis strain I136	NZ_CP011723.1	4107454
Bordetella pertussis strain I110	NZ_CP011718.1	4111608
Bordetella pertussis strain H884	NZ_CP011713.1	4109547
Bordetella pertussis strain H853	NZ_CP011708.1	4109537
Bordetella pertussis strain H779	NZ_CP011703.1	4106294
Bordetella pertussis strain H764	NZ_CP011698.1	4105334
Bordetella pertussis strain E530	NZ_CP011693.1	4106392
Bordetella pertussis strain E024	NZ_CP011692.1	4101131
Bordetella pertussis strain C742	NZ_CP011688.1	4103249
Bordetella pertussis strain I975	NZ_CP011242.1	4106497
Bordetella pertussis strain I112	NZ_CP011241.1	4106385
Bordetella pertussis strain J198	NZ_CP013902.1	4113756
Bordetella pertussis strain H851	NZ_CP011237.1	4106500
Bordetella pertussis strain J162	NZ_CP013899.1	4102183
Bordetella pertussis strain J155	NZ_CP013896.1	4106378
Bordetella pertussis strain H710	NZ_CP011236.1	4105333
Bordetella pertussis strain H639	NZ_CP012130.1	4088698
Bordetella pertussis strain I735	NZ_CP011742.1	4105343
Bordetella pertussis strain J013	NZ_CP012086.1	4105333
Bordetella pertussis strain I382	NZ_CP011737.1	4110505
Bordetella pertussis strain I351	NZ_CP012081.1	4108752
Bordetella pertussis strain I331	NZ_CP011732.1	4104202
Bordetella pertussis strain I944	NZ_CP011207.1	4112738
Bordetella pertussis strain I602	NZ_CP011202.1	4108484
Bordetella pertussis strain I271	NZ_CP011727.1	4109543
Bordetella pertussis strain H812	NZ_CP011197.1	4106501
Bordetella pertussis strain H765	NZ_CP011192.1	4103252
Bordetella pertussis strain I135	NZ_CP011722.1	4109544
Bordetella pertussis strain H703	NZ_CP011187.1	4107439
Bordetella pertussis strain I089	NZ_CP011717.1	4110588
Bordetella pertussis strain H665	NZ_CP011186.1	4109528
Bordetella pertussis strain F948	NZ_CP011182.1	4106406
Bordetella pertussis strain H883	NZ_CP011712.1	4109547
Bordetella pertussis strain F687	NZ_CP011181.1	4108495
Bordetella pertussis strain H849	NZ_CP011707.1	4103204
Bordetella pertussis strain F034	NZ_CP011177.1	4105889
Bordetella pertussis strain F011	NZ_CP011176.1	4102201
Bordetella pertussis strain H847	NZ_CP011706.1	4103252
Bordetella pertussis strain E555	NZ_CP011172.1	4102209
Bordetella pertussis strain E150	NZ_CP011171.1	4104313
Bordetella pertussis strain J174	NZ_CP013900.1	4108659
Bordetella pertussis strain H775	NZ_CP011702.1	4106491
Bordetella pertussis strain I959	NZ_CP011746.1	4104353
Bordetella pertussis strain I728	NZ_CP011741.1	4107398
Bordetella pertussis strain H773	NZ_CP011701.1	4111638
Bordetella pertussis strain I380	NZ_CP011736.1	4091930
Bordetella pertussis strain I318	NZ_CP011731.1	4107427
Bordetella pertussis strain H763	NZ_CP011697.1	4110595
Bordetella pertussis strain I270	NZ_CP011726.1	4103217
Bordetella pertussis strain I127	NZ_CP011721.1	4103220
Bordetella pertussis strain I113	NZ_CP011720.1	4103237
Bordetella pertussis strain H762	NZ_CP011696.1	4105337
Bordetella pertussis strain I088	NZ_CP011716.1	4112692
Bordetella pertussis strain D502	NZ_CP011691.1	4101167
Bordetella pertussis strain I069	NZ_CP011715.1	4109549
Bordetella pertussis strain H878	NZ_CP011711.1	4106327
Bordetella pertussis strain C505	NZ_CP011687.1	4103277
Bordetella pertussis strain H866	NZ_CP011710.1	4111644
Bordetella pertussis strain I075	NZ_CP011240.1	4106355
Bordetella pertussis strain H842	NZ_CP011705.1	4111642
Bordetella pertussis strain H768	NZ_CP011700.1	4106385
Bordetella pertussis strain H834	NZ_CP011235.1	4103704
Bordetella pertussis strain H437	NZ_CP011695.1	4105342
Bordetella pertussis strain D321	NZ_CP011690.1	4104176
Bordetella pertussis strain I464	NZ_CP012134.1	4107433
Bordetella pertussis strain J022	NZ_CP011244.1	4106504
Bordetella pertussis strain H915	NZ_CP011239.1	4104277
Bordetella pertussis strain H320	NZ_CP011234.1	4109545

Bordetella pertussis strain I965	NZ_CP011747.1	4108598
Bordetella pertussis strain G057	NZ_CP012129.1	4108471
Bordetella pertussis strain I762	NZ_CP011745.1	4110592
Bordetella pertussis strain I968	NZ_CP011748.1	4107604
Bordetella pertussis strain J010	NZ_CP012085.1	4107382
Bordetella pertussis strain I106	NZ_CP012080.1	4102205
Bordetella pertussis strain I461	NZ_CP011740.1	4106406
Bordetella pertussis strain I915	NZ_CP011206.1	4106386
Bordetella pertussis strain I379	NZ_CP011735.1	4104294
Bordetella pertussis strain I387	NZ_CP011201.1	4108494
Bordetella pertussis strain I755	NZ_CP011744.1	4111653
Bordetella pertussis strain I373	NZ_CP011200.1	4105954
Bordetella pertussis strain I375	NZ_CP011734.1	4113693
Bordetella pertussis strain H810	NZ_CP011196.1	4106384
Bordetella pertussis strain I754	NZ_CP011743.1	4109174
Bordetella pertussis strain H806	NZ_CP011195.1	4103534
Bordetella pertussis strain I452	NZ_CP011739.1	4109548
Bordetella pertussis strain I315	NZ_CP011730.1	4108498
Bordetella pertussis strain H754	NZ_CP011191.1	4101757
Bordetella pertussis strain I289	NZ_CP011729.1	4106397
Bordetella pertussis strain H637	NZ_CP011185.1	4103696
Bordetella pertussis strain I263	NZ_CP011725.1	4104253
Bordetella pertussis strain I257	NZ_CP011724.1	4104251
Bordetella pertussis strain D879	NZ_CP011170.1	4106400
Bordetella pertussis strain I187	NZ_CP012132.1	4106507
Bordetella pertussis strain I111	NZ_CP011719.1	4106381
Bordetella pertussis strain I150	NZ_CP012131.1	4107430
Bordetella pertussis strain J107	NZ_CP012088.1	4106377
Bordetella pertussis strain H910	NZ_CP011714.1	4110589
Bordetella pertussis strain J038	NZ_CP012087.1	4107443
Bordetella pertussis strain I743	NZ_CP012082.1	4107480
Bordetella pertussis strain H876	NZ_CP013882.1	4105347
Bordetella pertussis strain H864	NZ_CP011709.1	4106500
Bordetella pertussis strain H533	NZ_CP013879.1	4108283
Bordetella pertussis strain G085	NZ_CP013874.1	4106376
Bordetella pertussis strain I730	NZ_CP011203.1	4106407
Bordetella pertussis strain H787	NZ_CP011704.1	4108479
Bordetella pertussis strain J311	NZ_CP013866.1	4103242
Bordetella pertussis strain I228	NZ_CP011198.1	4111652
Bordetella pertussis strain H766	NZ_CP011699.1	4104260
Bordetella pertussis strain J197	NZ_CP013094.1	4107421
Bordetella pertussis strain H784	NZ_CP011193.1	4106396
Bordetella pertussis strain J193	NZ_CP013092.1	4105331
Bordetella pertussis strain H707	NZ_CP011188.1	4107280
Bordetella pertussis strain J192	NZ_CP013091.1	4112694
Bordetella pertussis strain H346	NZ_CP011694.1	4110608
Bordetella pertussis strain H520	NZ_CP011183.1	4102661
Bordetella pertussis strain J179	NZ_CP013089.1	4113730
Bordetella pertussis strain F658	NZ_CP011178.1	4107481
Bordetella pertussis strain D175	NZ_CP011689.1	4108515
Bordetella pertussis strain J178	NZ_CP013088.1	4107422
Bordetella pertussis strain E587	NZ_CP011173.1	4101625
Bordetella pertussis strain H682	NZ_CP013083.1	4104309
Bordetella pertussis strain C958	NZ_CP011168.1	4100254
Bordetella pertussis strain I998	NZ_CP011243.1	4109711
Bordetella pertussis strain C571	NZ_CP011167.1	4105364
Bordetella pertussis strain J039	NZ_CP011758.1	4107448
Bordetella pertussis strain H911	NZ_CP011238.1	4102182
Bordetella pertussis strain J023	NZ_CP011755.1	4106503
Bordetella pertussis strain J103	NZ_CP013887.1	4107419
Bordetella pertussis strain I259	NZ_CP012133.1	4105353
Bordetella pertussis strain J012	NZ_CP011751.1	4110595
Bordetella pertussis strain J099	NZ_CP013886.1	4112678
Bordetella pertussis strain I977	NZ_CP011749.1	4111640
Bordetella pertussis strain H361	NZ_CP013878.1	4107431
Bordetella pertussis strain I976	NZ_CP012084.1	4104251
Bordetella pertussis strain G807	NZ_CP013875.1	4102202
Bordetella pertussis strain F934	NZ_CP013873.1	4105353
Bordetella pertussis strain I751	NZ_CP012083.1	4109556
Bordetella pertussis strain F657	NZ_CP013871.1	4104234
Bordetella pertussis strain F501	NZ_CP013870.1	4101167
Bordetella pertussis strain H852	NZ_CP012079.1	4105340
Bordetella pertussis strain C975	NZ_CP013868.1	4104403
Bordetella pertussis strain J225	NZ_CP013096.1	4106294
Bordetella pertussis strain H681	NZ_CP012078.1	4105309
Bordetella pertussis strain J194	NZ_CP013093.1	4106379
Bordetella pertussis strain J191	NZ_CP013090.1	4111645
Bordetella pertussis strain I763	NZ_CP011205.1	4106397
Bordetella pertussis strain H771	NZ_CP013087.1	4105345
Bordetella pertussis strain H382	NZ_CP013082.1	4106392
Bordetella pertussis strain I752	NZ_CP011204.1	4110589
Bordetella pertussis strain C757	NZ_CP013079.1	4106366

Bordetella pertussis strain I238	NZ_CP011199.1	4104580
Bordetella pertussis strain J110	NZ_CP011767.1	4106384
Bordetella pertussis strain H800	NZ_CP011194.1	4102311
Bordetella pertussis strain J091	NZ_CP011764.1	4106381
Bordetella pertussis strain H729	NZ_CP011189.1	4108484
Bordetella pertussis strain J076	NZ_CP011762.1	4105464
Bordetella pertussis strain H579	NZ_CP011184.1	4109506
Bordetella pertussis strain J072	NZ_CP011760.1	4113747
Bordetella pertussis strain F670	NZ_CP011179.1	4103215
Bordetella pertussis strain I978	NZ_CP011750.1	4110591
Bordetella pertussis strain E809	NZ_CP011174.1	4103251
Bordetella pertussis strain J122	NZ_CP013892.1	4107596
Bordetella pertussis strain D521	NZ_CP011169.1	4101097
Bordetella pertussis strain J224	NZ_CP017164.1	4106189
Bordetella pertussis strain J226	NZ_CP017159.1	4107365
Bordetella pertussis strain J170	NZ_CP017166.1	4105315
Bordetella pertussis strain J151	NZ_CP017161.1	4106441
Bordetella pertussis strain J308	NZ_CP017167.1	4103236
Bordetella pertussis strain J109	NZ_CP017162.1	4106343
Bordetella pertussis strain J310	NZ_CP017168.1	4102178
Bordetella pertussis strain J108	NZ_CP017163.1	4106326
Bordetella pertussis strain J165	NZ_CP017158.1	4111636
Bordetella pertussis strain J276	NZ_CP013906.1	4108478
Bordetella pertussis strain J175	NZ_CP013901.1	4106390
Bordetella pertussis strain J152	NZ_CP013895.1	4110587
Bordetella pertussis strain F013	NZ_CP016965.1	4101245
Bordetella pertussis strain J207	NZ_CP018038.1	4108318
Bordetella pertussis strain J173	NZ_CP017924.1	4106356
Bordetella pertussis strain J313	NZ_CP018042.1	4109385
Bordetella pertussis strain J306	NZ_CP018041.1	4103205
Bordetella pertussis strain J295	NZ_CP017927.1	4105292
Bordetella pertussis strain J230	NZ_CP017926.1	4106384
Bordetella pertussis strain J304	NZ_CP018040.1	4105872
Bordetella pertussis strain J203	NZ_CP017925.1	4104273
Bordetella pertussis strain I384	NZ_CP017882.1	4108491
Bordetella pertussis strain J094	NZ_CP018037.1	4103789
Bordetella pertussis strain J148	NZ_CP017923.1	4102575
Bordetella pertussis strain J081	NZ_CP017885.1	4107548
Bordetella pertussis strain I385	NZ_CP017883.1	4105355
Bordetella pertussis strain J074	NZ_CP017884.1	4105450
Bordetella pertussis strain J294	NZ_CP018039.1	4109951
Bordetella pertussis strain J625	NZ_CP022362.1	4110338
Bordetella pertussis strain J078	NZ_CP021401.1	4100915
Bordetella pertussis strain J473	NZ_CP021403.1	4114583
Bordetella pertussis strain H696	NZ_CP021402.1	4103125
Bordetella pertussis strain J105	NZ_CP013889.1	4106364
Bordetella pertussis strain J277	NZ_CP013907.1	4108658
Bordetella pertussis strain J210	NZ_CP013905.1	4110596
Bordetella pertussis strain VA-175	NZ_CP015760.1	4110002
Bordetella pertussis strain VA-145	NZ_CP015769.1	4116920
Bordetella pertussis strain VA-62	NZ_CP015768.1	4115775
Bordetella pertussis strain VA-190	NZ_CP015761.1	4127222
Bordetella pertussis strain VA-09	NZ_CP015765.1	4113052
Bordetella pertussis strain VA-150	NZ_CP015762.1	4113555
Bordetella pertussis strain VA-194	NZ_CP015759.1	4115166
Bordetella pertussis strain VA-198	NZ_CP015764.1	4128711
Bordetella pertussis strain VA-10	NZ_CP015770.1	4121725
Bordetella pertussis strain VA-15	NZ_CP015766.1	4120993
Bordetella pertussis strain VA-52	NZ_CP015763.1	4123407
Bordetella pertussis strain VA-18	NZ_CP015767.1	4147141
Bordetella pertussis strain B3585	NZ_CP011444.1	4106388
Bordetella pertussis strain B1838	NZ_CP011440.1	4108472
Bordetella pertussis strain B3640	NZ_CP011445.1	4110990
Bordetella pertussis strain B3913	NZ_CP011447.1	4109548
Bordetella pertussis strain B3658	NZ_CP011446.1	4103254
Bordetella pertussis strain B1865	NZ_CP011441.1	4105363
Bordetella pertussis strain B3921	NZ_CP011448.1	4111557
Bordetella pertussis strain B3582	NZ_CP011443.1	4104305
Bordetella pertussis strain B3405	NZ_CP011442.1	4109988
Bordetella pertussis strain B3621	NZ_CP011401.1	4100705
Bordetella pertussis strain B3629	NZ_CP011400.1	4101147
Bordetella pertussis strain FR5810	NZ_LR130529.1	4108173
Bordetella pertussis strain FR5810	NZ_CP031788.1	4108173
Bordetella pertussis strain J697	NZ_CP026887.1	4108309
Bordetella pertussis strain FDAARGOS_179	NZ_CP014153.1	4100859
Bordetella pertussis strain I094	NZ_CP018036.1	4111426
Bordetella pertussis strain J223	NZ_CP017160.1	4107128
Bordetella pertussis strain J222	NZ_CP017165.1	4106009
Bordetella pertussis strain J818	CP043236.1	4104329
Bordetella pertussis strain J820	CP043235.1	4104341
Bordetella pertussis strain J083	CP043237.1	4148690
Bordetella pertussis strain UK76	CP031114.1	4112629

Bordetella pertussis strain UK39	CP031113.1	4108152
Bordetella pertussis strain UK38	CP031112.1	4108026
Bordetella pertussis strain UK36	CP031289.1	4107923
Bordetella pertussis strain I072	CP033419.1	4113684
Bordetella pertussis strain I080	CP033418.1	4111610
Bordetella pertussis strain J331	CP033417.1	4107426
Bordetella pertussis strain J348	CP033416.1	4104398
Bordetella pertussis strain J361	CP033415.1	4110579
Bordetella pertussis strain J420	CP033414.1	4107427
Bordetella pertussis strain J430	CP033413.1	4106650
Bordetella pertussis strain J437	CP033412.1	4107430
Bordetella pertussis strain J450	CP033411.1	4106387
Bordetella pertussis strain J672	CP033410.1	4102159
Bordetella pertussis strain J701	CP033409.1	4107362
Bordetella pertussis strain J865	CP033408.1	4104272
Bordetella pertussis strain K005	CP033407.1	4104259
Bordetella pertussis strain K006	CP033406.1	4104253
Bordetella pertussis strain J763	CP033309.1	4106386
Bordetella pertussis strain J797	CP033308.1	4107394
Bordetella pertussis strain J798	CP033307.1	4107422
Bordetella pertussis strain J799	CP033306.1	4107425
Bordetella pertussis strain J800	CP033305.1	4107418
Bordetella pertussis strain J801	CP033304.1	4106377
Bordetella pertussis strain J802	CP033303.1	4104246
Bordetella pertussis strain J803	CP033302.1	4104248
Bordetella pertussis strain J804	CP033301.1	4104252
Bordetella pertussis strain J805	CP033300.1	4106377
Bordetella pertussis strain J810	CP033299.1	4100123
Bordetella pertussis strain J811	CP033298.1	4100125
Bordetella pertussis strain J812	CP033297.1	4100121
Bordetella pertussis strain J813	CP033296.1	4100121
Bordetella pertussis strain J816	CP033295.1	4104343
Bordetella pertussis strain J819	CP033294.1	4104340
Bordetella pertussis strain J836	CP033293.1	4107383
Bordetella pertussis strain J837	CP033292.1	4106331
Bordetella pertussis strain J844	CP033291.1	4107419
Bordetella pertussis strain J846	CP033290.1	4108417
Bordetella pertussis strain J847	CP033289.1	4108425
Bordetella pertussis strain J848	CP033288.1	4108423
Bordetella pertussis strain J849	CP033287.1	4108415
Bordetella pertussis strain J851	CP033286.1	4108498
Bordetella pertussis strain J852	CP033285.1	4108513
Bordetella pertussis strain J854	CP033284.1	4108501
Bordetella pertussis strain J855	CP033283.1	4111618
Bordetella pertussis strain J856	CP033282.1	4111614
Bordetella pertussis strain J866	CP033281.1	4104067
Bordetella pertussis strain J867	CP033280.1	4103215
Bordetella pertussis strain J876	CP033279.1	4105456
Bordetella pertussis strain J878	CP033278.1	4101160
Bordetella pertussis strain J879	CP033277.1	4101171
Bordetella pertussis strain J880	CP033276.1	4101169
Bordetella pertussis strain J881	CP033275.1	4108586
Bordetella pertussis strain J882	CP033274.1	4108592
Bordetella pertussis strain J883	CP033273.1	4108590
Bordetella pertussis strain J884	CP033272.1	4108587
Bordetella pertussis strain J885	CP033271.1	4105452
Bordetella pertussis strain J886	CP033270.1	4105457
Bordetella pertussis strain J887	CP033269.1	4105455
Bordetella pertussis strain J888	CP033268.1	4105456
Bordetella pertussis strain J889	CP033267.1	4105456
Bordetella pertussis strain J890	CP033266.1	4105455
Bordetella pertussis strain J907	CP033265.1	4104089
Bordetella pertussis strain K003	CP033264.1	4104246
Bordetella pertussis strain K007	CP033263.1	4104244
Bordetella pertussis strain K011	CP033262.1	4108452
Bordetella pertussis strain K012	CP033261.1	4108443
Bordetella pertussis strain K013	CP033260.1	4108429
Bordetella pertussis strain K014	CP033259.1	4108465
Bordetella pertussis strain K015	CP033258.1	4110560
Bordetella pertussis strain J704	CP032787.1	4105112
Bordetella pertussis strain J705	CP032786.1	4105103
Bordetella pertussis strain J706	CP032785.1	4105082
Bordetella pertussis strain J736	CP032784.1	4103246
Bordetella pertussis strain J738	CP032783.1	4107409
Bordetella pertussis strain J743	CP032782.1	4105314
Bordetella pertussis strain J744	CP032781.1	4105289
Bordetella pertussis strain J745	CP032780.1	4105313
Bordetella pertussis strain J746	CP032779.1	4105330
Bordetella pertussis strain J747	CP032778.1	4105303
Bordetella pertussis strain J748	CP032777.1	4105310
Bordetella pertussis strain J751	CP032776.1	4105352
Bordetella pertussis strain J752	CP032775.1	4105270

Bordetella pertussis strain J753	CP032774.1	4104307
Bordetella pertussis strain J755	CP032773.1	4104300
Bordetella pertussis strain J758	CP032772.1	4108475
Bordetella pertussis strain J759	CP032771.1	4108481
Bordetella pertussis strain J760	CP032770.1	4108481
Bordetella pertussis strain J761	CP032769.1	4108469
Bordetella pertussis strain J762	CP032768.1	4108476
Bordetella pertussis strain J764	CP032767.1	4106379
Bordetella pertussis strain J766	CP032766.1	4106389
Bordetella pertussis strain J780	CP032765.1	4108442
Bordetella pertussis strain J781	CP032764.1	4108419
Bordetella pertussis strain J782	CP032763.1	4108394
Bordetella pertussis strain J261	CP032984.1	4109667
Bordetella pertussis strain J271	CP032983.1	4108382
Bordetella pertussis strain J273	CP032982.1	4110530
Bordetella pertussis strain J314	CP032981.1	4102869
Bordetella pertussis strain J315	CP032980.1	4107522
Bordetella pertussis strain J316	CP032979.1	4106594
Bordetella pertussis strain J317	CP032978.1	4104255
Bordetella pertussis strain J319	CP032977.1	4109520
Bordetella pertussis strain J341	CP032976.1	4109524
Bordetella pertussis strain J351	CP032975.1	4108462
Bordetella pertussis strain J352	CP032974.1	4107601
Bordetella pertussis strain J353	CP032973.1	4106353
Bordetella pertussis strain J355	CP032985.1	4109519
Bordetella pertussis strain J356	CP032972.1	4106376
Bordetella pertussis strain J383	CP032971.1	4107424
Bordetella pertussis strain J388	CP032970.1	4106382
Bordetella pertussis strain J399	CP032969.1	4101972
Bordetella pertussis strain J400	CP032968.1	4113747
Bordetella pertussis strain J405	CP032967.1	4107542
Bordetella pertussis strain J406	CP032966.1	4111624
Bordetella pertussis strain J407	CP032965.1	4108480
Bordetella pertussis strain J408	CP032964.1	4112708
Bordetella pertussis strain J409	CP032963.1	4107436
Bordetella pertussis strain J410	CP032962.1	4107376
Bordetella pertussis strain J421	CP032961.1	4108425
Bordetella pertussis strain J422	CP032960.1	4108431
Bordetella pertussis strain J433	CP032959.1	4104279
Bordetella pertussis strain J435	CP032958.1	4107407
Bordetella pertussis strain J436	CP032957.1	4103242
Bordetella pertussis strain J438	CP032956.1	4105192
Bordetella pertussis strain J439	CP032955.1	4108480
Bordetella pertussis strain J451	CP032954.1	4110582
Bordetella pertussis strain J452	CP032953.1	4103510
Bordetella pertussis strain J453	CP032952.1	4107429
Bordetella pertussis strain J454	CP032951.1	4106383
Bordetella pertussis strain J455	CP032950.1	4107731
Bordetella pertussis strain J457	CP032949.1	4106673
Bordetella pertussis strain J458	CP032948.1	4106675
Bordetella pertussis strain J459	CP032947.1	4106674
Bordetella pertussis strain J460	CP032946.1	4107727
Bordetella pertussis strain J461	CP032945.1	4106670
Bordetella pertussis strain J462	CP032944.1	4107724
Bordetella pertussis strain J196	CP032736.1	4283313
Bordetella pertussis strain J318	CP032735.1	4274138
Bordetella pertussis strain J321	CP032734.1	4155468
Bordetella pertussis strain J475	CP032733.1	4114650
Bordetella pertussis strain J476	CP032732.1	4107450
Bordetella pertussis strain J477	CP032731.1	4114616
Bordetella pertussis strain J487	CP032730.1	4114643
Bordetella pertussis strain J493	CP032729.1	4115686
Bordetella pertussis strain J494	CP032728.1	4107493
Bordetella pertussis strain J495	CP032727.1	4103260
Bordetella pertussis strain J496	CP032726.1	4104278
Bordetella pertussis strain J497	CP032725.1	4109705
Bordetella pertussis strain J591	CP032737.1	4105341
Bordetella pertussis strain J592	CP032724.1	4107705
Bordetella pertussis strain J595	CP032723.1	4106387
Bordetella pertussis strain J597	CP032722.1	4109699
Bordetella pertussis strain J598	CP032721.1	4110563
Bordetella pertussis strain J600	CP032720.1	4106376
Bordetella pertussis strain J601	CP032719.1	4109503
Bordetella pertussis strain J608	CP032718.1	4114767
Bordetella pertussis strain J609	CP032717.1	4110569
Bordetella pertussis strain J610	CP032716.1	4113726
Bordetella pertussis strain J612	CP032715.1	4113725
Bordetella pertussis strain J618	CP032714.1	4103204
Bordetella pertussis strain J619	CP032713.1	4108569
Bordetella pertussis strain J620	CP032712.1	4109601
Bordetella pertussis strain J622	CP032711.1	4106490
Bordetella pertussis strain J623	CP032710.1	4107428

Bordetella pertussis strain J631	CP032709.1	4103226
Bordetella pertussis strain J638	CP032708.1	4106671
Bordetella pertussis strain J299	CP046994.1	4291088
Bordetella pertussis strain D800	CP046992.1	4167908
Bordetella pertussis strain J349	CP046991.1	4260789
Bordetella pertussis strain J385	CP046990.1	4151620
Bordetella pertussis strain J412	CP046989.1	4125429
Bordetella pertussis strain J733	CP046988.1	4191912
Bordetella pertussis strain J737	CP046987.1	4285194
Bordetella pertussis strain J739	CP046986.1	4149262
Bordetella pertussis strain J740	CP046985.1	4149269
Bordetella pertussis strain J741	CP046984.1	4149088
Bordetella pertussis strain J742	CP046983.1	4149155
Bordetella pertussis strain J767	CP046982.1	4182922
Bordetella pertussis strain J016	NZ_CP011752.1	4103226
Bordetella pertussis strain J018	NZ_CP011208.1	4103225
G12 Bp H677		
GCGCATGCGTGCAGATTCGTCGTACAAAATCCTCGATTCTTCCGTACATCCCGC TACTGCAATCCAACACGGCATGAACGCTCCTTCGGCGCAAAGTCGCGCGATAGT ACCGGTCACCGTCCGGACCGTGCTGACCCCCCTGCCATGGTGTGATCCGTAAAA TAGGCACCATCAAAACG		
Bordetella pertussis strain H677	NZ_CP025367.1	4106930
G13 Bp J029		
GCGCATGCGTGCAGATTCGTCGTACAAAACCCTCGATTCTTCCGTACATCCCGT TACTGCAATCCAACACGGCATGAACGCTCCTTCGGCGCAAAGTCGCGCGATAGT ACCGGTCACCGTCCGGACCGTGCTGACCCCCCTGCCATGGTGTGATCCGTAAAA TAGGCACCATCAAAACG		
Bordetella pertussis strain J029	CP046995.1	4156535
G14 Bp Tohama I		
GCGCATGCGTGCAGATTCGTCGTACAAAACCCTCGATTCTTCCGTACATCCCGC TACTGCAATCCAACACGGCATGAACGCTCCTTCGGCGCAAAGTCGCGCGATGGT ACCGGTCACCGTCCGGACCGTGCTGACCCCCCTGCCATGGTGTGATCCGTAAAA TAGGCACCATCAAAACG		
Bordetella pertussis Tohama I	NC_002929.2	4086189
Bordetella pertussis strain FDAARGOS_1368	NZ_CP069820.1	4105067
Bordetella pertussis strain FDAARGOS_1366	NZ_CP070102.1	4105436
Bordetella pertussis strain FDAARGOS_1369	NZ_CP069843.1	4104031
Bordetella pertussis strain FDAARGOS_1365	NZ_CP070101.1	4105080
Bordetella pertussis strain F954	NZ_CP025366.1	4112103
Bordetella pertussis strain NCTC13251	NZ_LR590467.1	4102960
Bordetella pertussis strain J363	NZ_CP026431.1	4113166
Bordetella pertussis Tohama I	NZ_CP039022.1	4086189
Bordetella pertussis strain VS401	NZ_LS398605.1	4162738
Bordetella pertussis strain VS377	NZ_LS398604.1	4160618
Bordetella pertussis strain VS393	NZ_LS398590.1	4160081
Bordetella pertussis strain VS366	NZ_LS398589.1	4144451
Bordetella pertussis strain Tohama	NZ_CP031787.1	4102412
Bordetella pertussis strain J042	NZ_CP019869.1	4162596
Bordetella pertussis strain J043	NZ_CP016887.1	4102813
Bordetella pertussis strain E476	NZ_CP010964.1	4102978
Bordetella pertussis strain H375	NZ_CP010961.1	4111050
Bordetella pertussis strain C393	NZ_CP010963.1	4133777
Bordetella pertussis strain CS	NC_017223.1	4124236
Bordetella pertussis strain H740	NZ_CP011190.1	4111081
Bordetella pertussis strain E976	NZ_CP011175.1	4116415
Bordetella pertussis strain B201	NZ_CP013075.1	4122649
Bordetella pertussis strain 134	NZ_CP017402.1	4128984
Bordetella pertussis strain J169	NZ_CP012089.1	4102567
Bordetella pertussis strain I344	NZ_CP011255.1	4111065
Bordetella pertussis strain Pelita III	NZ_CP019957.1	4105522
Bordetella pertussis strain B202	NZ_CP016338.1	4128979
Bordetella pertussis strain UT25Sm1	NZ_CP015771.1	4124767
Bordetella pertussis strain P2013109	NZ_CP038790.1	4126010
Bordetella pertussis strain BPD2	NZ_CP034101.1	4104911
Bordetella pertussis strain B228	NZ_CP026465.1	4133306
Bordetella pertussis strain A339	NZ_CP026634.1	4131102
Bordetella pertussis strain VS67	NZ_LS398588.1	4137378
Bordetella pertussis strain BPD1	NZ_CP034182.1	4126211
Bordetella pertussis strain NCTC10910	NZ_LS483398.1	4133344
Bordetella pertussis strain B1920	NZ_CP009752.1	4114630
Bordetella pertussis strain B226	NZ_CP016957.1	4126037
Bordetella pertussis strain E945	NZ_CP016956.1	4127835
Bordetella pertussis strain B227	NZ_CP013076.1	4134432
Bordetella pertussis strain 25525	NZ_CP017405.1	4386396
Bordetella pertussis strain 6229	NZ_CP017404.1	4257407
Bordetella pertussis strain A340	CP033420.1	4131102
Bordetella pertussis strain A639	CP046993.1	4181656

Supplementary Table S2. PB plasmids constructed for this study.*

**Plasmid**	**PB**	**Description**
pQC1114		P*ptx*^{per}, WT
pQC1284		P*ptx*^{bro}, WT
pQC1285	PB1	See Fig. 1 and supplementary Fig. S1
pQC1286	PB2	See Fig. 1 and supplementary Fig. S1
pQC1287	PB3	See Fig. 1 and supplementary Fig. S1
pQC1288	PB4	See Fig. 1 and supplementary Fig. S1
pQC1289	PB5	See Fig. 1 and supplementary Fig. S1
pQC1290	PB6	See Fig. 1 and supplementary Fig. S1
pQC1291	PB7	See Fig. 1 and supplementary Fig. S1
pQC1292	PB8	See Fig. 1 and supplementary Fig. S1
pQC1293	PB9	See Fig. 1 and supplementary Fig. S1
pQC1294	PB10	See Fig. 1 and supplementary Fig. S1
pQC1295	PB11	See Fig. 1 and supplementary Fig. S1
pQC1296	PB12	See Fig. 1 and supplementary Fig. S1
pQC1355	PB13	P*ptx*^{bro}, C^{-13}T
pQC1356	PB14	P*ptx*^{bro}, G^{-3}A
pQC1357	PB15	P*ptx*^{bro}, C^{+2}T
pQC1358	PB16	P*ptx*^{bro}, G^{+4}A
pQC1359	PB17	P*ptx*^{bro}, C^{-13}T, G^{-3}A
pQC1360	PB18	P*ptx*^{bro}, C^{-13}T, C^{+2}T
pQC1361	PB19	P*ptx*^{bro}, C^{-13}T, G^{+4}A
pQC1362	PB20	P*ptx*^{bro}, G^{-3}A, C^{+2}T
pQC1363	PB21	P*ptx*^{bro}, G^{-3}A, G^{+4}A
pQC1364	PB22	P*ptx*^{bro}, C^{+2}T, G^{+4}A
pQC1365	PB23	P*ptx*^{bro}, C^{-13}T, G^{-3}A, C^{+2}T
pQC1366	PB24	P*ptx*^{bro}, C^{-13}T, G^{-3}A, G^{+4}A
pQC1367	PB25	P*ptx*^{bro}, C^{-13}T, C^{+2}T, G^{+4}A
pQC1368	PB26	P*ptx*^{bro}, G^{-3}A, C^{+2}T, G^{+4}A
pQC1369	PB27	P*ptx*^{per}, T^{-13}C
pQC1370	PB28	P*ptx*^{per}, A^{-3}G
pQC1371	PB29	P*ptx*^{per}, T^{+2}C
pQC1372	PB30	P*ptx*^{per}, A^{+4}G
pQC1373	PB31	P*ptx*^{per}, T^{-13}C, A^{-3}G
pQC1374	PB32	P*ptx*^{per}, T^{-13}C, T^{+2}C
pQC1375	PB33	P*ptx*^{per}, T^{-13}C, A^{+4}G
pQC1376	PB34	P*ptx*^{per}, A^{-3}G, T^{+2}C
pQC1377	PB35	P*ptx*^{per}, A^{-3}G, A^{+4}G
pQC1378	PB36	P*ptx*^{per}, T^{+2}C, A^{+4}G
pQC1379	PB37	P*ptx*^{per}, T^{-13}C, A^{-3}G, T^{+2}C
pQC1380	PB38	P*ptx*^{per}, T^{-13}C, A^{-3}G, A^{+4}G
pQC1381	PB39	P*ptx*^{per}, T^{-13}C, T^{+2}C, A^{+4}G
pQC1382	PB40	P*ptx*^{per}, A^{-3}G, T^{+2}C, A^{+4}G
pQC1514	PB41	PB1, C^{-13}T

pQC1515	PB42	PB2, $C^{-13}T$
pQC1516	PB43	PB3, $C^{-13}T$
pQC1517	PB44	PB4, $C^{-13}T$
pQC1518	PB45	PB5, $C^{-13}T$
pQC1519	PB46	PB6, $C^{-13}T$
pQC1520	PB47	PB7, $T^{-13}C$
pQC1521	PB48	PB8, $T^{-13}C$
pQC1522	PB49	PB9, $T^{-13}C$
pQC1523	PB50	PB10, $T^{-13}C$
pQC1524	PB51	PB11, $T^{-13}C$
pQC1525	PB52	PB12, $T^{-13}C$
pQC1470	PB53	$Pptx^{bro}$, $C^{-13}T$, $C^{-39}T$
pQC1471	PB54	$Pptx^{bro}$, $C^{-13}T$, $T^{-44}C$
pQC1472	PB55	$Pptx^{bro}$, $C^{-13}T$, $A^{-48}G$
pQC1473	PB56	$Pptx^{bro}$, $C^{-13}T$, $G^{-55}A$
pQC1475	PB57	$Pptx^{bro}$, $C^{-13}T$, $A^{-70}G$
pQC1476	PB58	$Pptx^{bro}$, $C^{-13}T$, $C^{-96}T$
pQC1477	PB59	$Pptx^{bro}$, $C^{-13}T$, $G^{-97}A$
pQC1478	PB60	$Pptx^{bro}$, $C^{-13}T$, $C^{-126}T$
pQC1479	PB61	$Pptx^{bro}$, $C^{-13}T$, $G^{-142}A$
pQC1480	PB62	$Pptx^{bro}$, $C^{-13}T$, $C^{-148}T$
pQC1481	PB63	$Pptx^{bro}$, $C^{-13}T$, $G^{-154}T$
pQC1482	PB64	$Pptx^{bro}$, $C^{-13}T$, $G^{-158}A$
pQC1483	PB65	$Pptx^{bro}$, $C^{-13}T$, $C^{-161}T$
pQC1484	PB66	$Pptx^{bro}$, $C^{-13}T$, $T^{-167}C$
pQC1530	PB67	$Pptx^{bro}$, $C^{-148}T$
pQC1531	PB68	$Pptx^{bro}$, $C^{-148}T$, $C^{-126}T$
pQC1532	PB69	$Pptx^{bro}$, $C^{-148}T$, $C^{-126}T$, $A^{-70}G$
pQC1533	PB70	$Pptx^{bro}$, $C^{-148}T$, $C^{-126}T$, $A^{-70}G$, $C^{-39}T$
pQC1534	PB71	$Pptx^{bro}$, $C^{-148}T$, $C^{-126}T$, $A^{-70}G$, $C^{-39}T$, $G^{-154}T$
pQC1536	PB72	$Pptx^{bro}$, $C^{-13}T$, $C^{-148}T$, $C^{-126}T$
pQC1537	PB73	$Pptx^{bro}$, $C^{-13}T$, $C^{-148}T$, $C^{-126}T$, $A^{-70}G$
pQC1538	PB74	$Pptx^{bro}$, $C^{-13}T$, $C^{-148}T$, $C^{-126}T$, $A^{-70}G$, $C^{-39}T$
pQC1539	PB75	$Pptx^{bro}$, $C^{-13}T$, $C^{-148}T$, $C^{-126}T$, $A^{-70}G$, $C^{-39}T$, $G^{-154}T$

*P*ptx* fragments (-290 ~+27) were cloned between the *Eco*RI and *Sal*I sites of pSS3967 to create P*ptx-lux* transcriptional fusions.

Supplementary Table S3. Primers used for cloning						
**Primer**	**SEQUENCE**	**Restriction sites**	**Template**	**Vector**	**Resulting Plasmid**	**Resulting Strain**
**Allelic exchange constructs**						
Q1446	TATGCGGCCGCCGGTGATGCCGTTGTGATAGAC	*Not* I	RB50	pSS4661	pQC1540	QC2335
Q1453	TATCCCGGGGGTCTCAAACGCAGAGGGGAAGACGGGATG	*Sma* I/*Bsa* I				
Q1452	TATCCCGGGGGTCTCTTGGCGGTTGCCAACACGTATG	*Sma* I/*Bsa* I	RB50			
Q1445	TATGGATCCCGTTCGCGCTCAACGCGCTGGGATC	*Bam* HI				
Q1454	TATGGTCTCCGCCAACGCGCATGCGTGCAGATTC	*Bsa* I	BP536	pSS4661	pQC1541	QC2336
Q1455	TATGGTCTCACGTTTTGATGGTGCCTATTTTACGGATCAC	*Bsa* I				
Q1446	TATGCGGCCGCCGGTGATGCCGTTGTGATAGAC	*Not* I	RB50	pSS4661	pQC1542	QC2337
Q1247	CGCGGTCTCGACCCCCCTGCCATGGTGTGATCCG**T**AAAATAGGC**G**CCA**C**C**G**AAACGC	*Bsa* I				
Q1261	CGCGGTCTCGGGGTCGGCACAGTCTGGACGGCGACCGGTA	*Bsa* I	RB50			
Q1445	TATGGATCCCGTTCGCGCTCAACGCGCTGGGATC	*Bam* HI				
Q1446	TATGCGGCCGCCGGTGATGCCGTTGTGATAGAC	*Not* I	pQC1542	pSS4661	pQC1543	QC2338
Q1382	TATGGTCTCCGATTCTTCCGCACATCCCGCTACTG	*Bsa* I				
Q1385	TATGGTCTCGAATCGAGGGCTTTGT**A**CGACGCATC	*Bsa* I	pQC1542			
Q1445	TATGGATCCCGTTCGCGCTCAACGCGCTGGGATC	*Bam* HI				
Q1446	TATGCGGCCGCCGGTGATGCCGTTGTGATAGAC	*Not* I	pQC1543	pSS4661	pQC1544	QC2339
Q1381	TATGGTCTCCGATTCTTCCG**T**ACATCCCGCTACTG	*Bsa* I				
Q1385	TATGGTCTCGAATCGAGGGCTTTGT**A**CGACGCATC	*Bsa* I	pQC1543			
Q1445	TATGGATCCCGTTCGCGCTCAACGCGCTGGGATC	*Bam* HI				
Q1446	TATGCGGCCGCCGGTGATGCCGTTGTGATAGAC	*Not* I	pQC1544	pSS4661	pQC1545	QC2340
Q1374	TATGGTCTCGGTACCGGTCGCCGTCCAGACTGTG	*Bsa* I				
Q1377	TATGGTCTCGGTACCATCG**C**GCGACTTTGCGCCGAAG	*Bsa* I	pQC1544			
Q1445	TATGGATCCCGTTCGCGCTCAACGCGCTGGGATC	*Bam* HI				
Q1446	TATGCGGCCGCCGGTGATGCCGTTGTGATAGAC	*Not* I	pQC1545	pSS4661	pQC1546	QC2341
Q1368	TATGGTCTCTGTGC**T**GACCCCCCTGCCATGGTG	*Bsa* I				
Q1370	TATGGTCTCGGCACAGTCTGGACGGCGACCGGTAC	*Bsa* I	pQC1545			
Q1445	TATGGATCCCGTTCGCGCTCAACGCGCTGGGATC	*Bam* HI				
Q1497	ATA CCCGGGTTCCACCGTGGAAACGGATGAAGG	*Xma* I	pSS4162	pQC1540	pQC1561	QC2331
Q1498	ATA CCCGGGAGCTTTTAGGTGGCGGTACTTGGGTC	*Xma* I				
***In situ lux* fusions**						
1710	CGCGGATCCGGTCTCGAATTCATCGCGGCGCCTGTCTCGGCCGACACC	*EcoR* I	RB50	pSS4162	pQC1526	
479	CGCGTCGACCA CCCGTCTTCCC TCTGCGTTTT	*Sal* I				
1710	CGCGGATCCGGTCTCGAATTCATCGCGGCGCCTGTCTCGGCCGACACC	*EcoR* I	QC2335	pSS4162	pQC1597	
Q1119	CGCGTCGACCATCCCGTCTTCCCCTCTGCGTTT	*Sal* I				
1708	CGCGAATTCGGGCGCGTCGCGCGATCAGGCGG	*EcoR* I	RB50	pSS4162	pQC1598	
Q1531	CGC GTC GAC CAAGAACCAAACATCCAGCACGTCG	*Sal* I				
**Ectopic *lux* fusions**						
Q1048	CGCGAATTCCCAGCGCTGGGCCGGCGTC	*EcoR* I	BP536	pSS3967	pQC1114	
479	CGCGTCGACCATCCCGTCTTCCCCTCTGCGTTTT	*Sal* I				
Q1048	CGCGAATTCCCAGCGCTGGGCCGGCGTC	*EcoR* I	RB50	pSS3967	pQC1284	
Q1119	CGCGTCGACCATCCCGTCTTCCCCTCTGCGTTT	*Sal* I				
Q1048	CGCGAATTCCCAGCGCTGGGCCGGCGTC	*EcoR* I	BP536	pSS3967	pQC1285	
Q1213	CGCGGTCTCTTTTGTACGACGAATCTGCACGCATGCG	*Bsa* I				
Q1200	CGCGGTCTCACAAAGCCCTCGATTCTTCCGCA	*Bsa* I	RB50			
Q1119	CGCGTCGACCATCCCGTCTTCCCCTCTGCGTTT	*Sal* I				
Q1048	CGCGAATTCCCAGCGCTGGGCCGGCGTC	*EcoR* I	BP536	pSS3967	pQC1286	
Q1215	CGCGGTCTCGGATGTACGGAAGAATCGAGGGTTTTGTACG	*Bsa* I				
Q1202	CGCGGTCTCACATCCCGCTACTGCAATCCAACAC	*Bsa* I	RB50			
Q1119	CGCGTCGACCATCCCGTCTTCCCCTCTGCGTTT	*Sal* I				
Q1048	CGCGAATTCCCAGCGCTGGGCCGGCGTC	*EcoR* I	BP536	pSS3967	pQC1287	
Q1217	CGCGGTCTCGCCGTGTTGGATTGCAGTAGCGGGATGTACG	*Bsa* I				
Q1204	CGCGGTCTCCACGGCGCGAACGCTCCTTCGGCGCAA	*Bsa* I	RB50			
Q1119	CGCGTCGACCATCCCGTCTTCCCCTCTGCGTTT	*Sal* I				
Q1048	CGCGAATTCCCAGCGCTGGGCCGGCGTC	*EcoR* I	BP536	pSS3967	pQC1288	
Q1219	CGCGGTCTCTTTGCGCCGAAGGAGCGTTCATGCCG	*Bsa* I				
Q1206	CGCGGTCTCCGCAAAGTCGCACGATGGTACCG	*Bsa* I	RB50			
Q1119	CGCGTCGACCATCCCGTCTTCCCCTCTGCGTTT	*Sal* I				
Q1048	CGCGAATTCCCAGCGCTGGGCCGGCGTC	*EcoR* I	BP536	pSS3967	pQC1289	
Q1221	CGCGGTCTCGACGGTGACCGGTACCATCGCG	*Bsa* I				
Q1208	CGCGGTCTCGCCGTCCAGACTGTGCCGACCC	*Bsa* I	RB50			
Q1119	CGCGTCGACCATCCCGTCTTCCCCTCTGCGTTT	*Sal* I				
Q1048	CGCGAATTCCCAGCGCTGGGCCGGCGTC	*EcoR* I	BP536	pSS3967	pQC1290	
Q1223	CGCGGTCTCGGGGGTCAGCACGGTCCGG	*Bsa* I				
Q1210	CGCGGTCTCACCCCCCTGCCATGGTGTGATCCGCA	*Bsa* I	RB50			
Q1119	CGCGTCGACCATCCCGTCTTCCCCTCTGCGTTT	*Sal* I				
Q1048	CGCGAATTCCCAGCGCTGGGCCGGCGTC	*EcoR* I	RB50	pSS3967	pQC1291	
Q1201	CGCGGTCTCCTTTGTGCGACGCATCCGCGCGC	*Bsa* I				
Q1212	CGCGGTCTCACAAAACCCTCGATTCTTCCGTACATCCCG	*Bsa* I	BP536			
Q1119	CGCGTCGACCATCCCGTCTTCCCCTCTGCGTTT	*Sal* I				
Q1048	CGCGAATTCCCAGCGCTGGGCCGGCGTC	*EcoR* I	RB50	pSS3967	pQC1292	
Q1203	CGCGGTCTCGGATGTGCGGAAGAATCGAGGGC	*Bsa* I				
Q1214	CGCGGTCTCACATCCCGCTACTGCAATCCAACACG	*Bsa* I	BP536			
Q1119	CGCGTCGACCATCCCGTCTTCCCCTCTGCGTTT	*Sal* I				
Q1048	CGCGAATTCCCAGCGCTGGGCCGGCGTC	*EcoR* I	RB50	pSS3967	pQC1293	
Q1205	CGCGGTCTCGCCGTGTTGGATTGCAGTAGCGG	*Bsa* I				
Q1216	CGCGGTCTCCACGGCATGAACGCTCCTTCG	*Bsa* I	BP536			
Q1119	CGCGTCGACCATCCCGTCTTCCCCTCTGCGTTT	*Sal* I				
Q1048	CGCGAATTCCCAGCGCTGGGCCGGCGTC	*EcoR* I	RB50	pSS3967	pQC1294	
Q1207	CGCGGTCTCTTTGCGCCGAAGGAGCGTTCG	*Bsa* I				
Q1218	CGCGGTCTCCGCAAAGTCGCGCGATGGTAC	*Bsa* I	BP536			
Q1119	CGCGTCGACCATCCCGTCTTCCCCTCTGCGTTT	*Sal* I				
Q1048	CGCGAATTCCCAGCGCTGGGCCGGCGTC	*EcoR* I	RB50	pSS3967	pQC1295	
Q1209	CGCGGTCTCGACGGCGACCGGTACCATCGTGCGACT	*Bsa* I				
Q1220	CGCGGTCTCACCGTCCGGACCGTGCTGA	*Bsa* I	BP536			
Q1119	CGCGTCGACCATCCCGTCTTCCCCTCTGCGTTT	*Sal* I				
Q1048	CGCGAATTCCCAGCGCTGGGCCGGCGTC	*EcoR* I	RB50	pSS3967	pQC1296	
Q1211	CGCGGTCTCGGGGGTCGGCACAGTCTGGACGGCG	*Bsa* I				
Q1222	CGCGGTCTCACCCCCCTGCCATGGTGTGATC	*Bsa* I	BP536			
Q1119	CGCGTCGACCATCCCGTCTTCCCCTCTGCGTTT	*Sal* I				
Q1048	CGCGAATTCCCAGCGCTGGGCCGGCGTC	*EcoR* I	RB50	pSS3967	pQC1355	
Q1261	CGCGGTCTCGGGGTCGGCACAGTCTGGACGGCGACCGGTA	*Bsa* I				
Q1247	CGCGGTCTCGACCCCCCTGCCATGGTGTGATCCG**T**AAAATAGGC**G**CCA**C**C**G**AAACGC	*Bsa* I	RB50			
Q1119	CGCGTCGACCATCCCGTCTTCCCCTCTGCGTTT	*Sal* I				
Q1048	CGCGAATTCCCAGCGCTGGGCCGGCGTC	*EcoR* I	RB50	pSS3967	pQC1356	
Q1261	CGCGGTCTCGGGGTCGGCACAGTCTGGACGGCGACCGGTA	*Bsa* I				
Q1248	CGCGGTCTCGACCCCCCTGCCATGGTGTGATCCG**C**AAAATAGGC**A**CCA**C**C**G**AAACGC	*Bsa* I	RB50			
Q1119	CGCGTCGACCATCCCGTCTTCCCCTCTGCGTTT	*Sal* I				
Q1048	CGCGAATTCCCAGCGCTGGGCCGGCGTC	*EcoR* I	RB50	pSS3967	pQC1357	
Q1261	CGCGGTCTCGGGGTCGGCACAGTCTGGACGGCGACCGGTA	*Bsa* I				
Q1249	CGCGGTCTCGACCCCCCTGCCATGGTGTGATCCG**C**AAAATAGGC**G**CCA**T**C**G**AAACGC	*Bsa* I	RB50			
Q1119	CGCGTCGACCATCCCGTCTTCCCCTCTGCGTTT	*Sal* I				
Q1048	CGCGAATTCCCAGCGCTGGGCCGGCGTC	*EcoR* I	RB50	pSS3967	pQC1358	
Q1261	CGCGGTCTCGGGGTCGGCACAGTCTGGACGGCGACCGGTA	*Bsa* I				
Q1250	CGCGGTCTCGACCCCCCTGCCATGGTGTGATCCG**C**AAAATAGGC**G**CCA**C**C**A**AAACGC	*Bsa* I	RB50			
Q1119	CGCGTCGACCATCCCGTCTTCCCCTCTGCGTTT	*Sal* I				

Q1048	CGCGAATTCCCAGCGCTGGGCCGGCGTC	*EcoR* I	RB50	pSS3967	pQC1359
Q1261	CGCGGTCTCGGGGTCGGCACAGTCTGGACGGCGACCGGTA	*Bsa* I			
Q1251	CGCGGTCTCGACCCCCCTGCCATGGTGTGATCCG**T**AAAATAGGC**A**CCA**C**C**G**AAACGC	*Bsa* I	RB50		
Q1119	CGCGTCGACCATCCCGTCTTCCCCTCTGCGTTT	*Sal* I			
Q1048	CGCGAATTCCCAGCGCTGGGCCGGCGTC	*EcoR* I	RB50	pSS3967	pQC1360
Q1261	CGCGGTCTCGGGGTCGGCACAGTCTGGACGGCGACCGGTA	*Bsa* I			
Q1252	CGCGGTCTCGACCCCCCTGCCATGGTGTGATCCG**T**AAAATAGGC**G**CCA**T**C**G**AAACGC	*Bsa* I	RB50		
Q1119	CGCGTCGACCATCCCGTCTTCCCCTCTGCGTTT	*Sal* I			
Q1048	CGCGAATTCCCAGCGCTGGGCCGGCGTC	*EcoR* I	RB50	pSS3967	pQC1361
Q1261	CGCGGTCTCGGGGTCGGCACAGTCTGGACGGCGACCGGTA	*Bsa* I			
Q1253	CGCGGTCTCGACCCCCCTGCCATGGTGTGATCCG**T**AAAATAGGC**G**CCA**C**C**A**AAACGC	*Bsa* I	RB50		
Q1119	CGCGTCGACCATCCCGTCTTCCCCTCTGCGTTT	*Sal* I			
Q1048	CGCGAATTCCCAGCGCTGGGCCGGCGTC	*EcoR* I	RB50	pSS3967	pQC1362
Q1261	CGCGGTCTCGGGGTCGGCACAGTCTGGACGGCGACCGGTA	*Bsa* I			
Q1254	CGCGGTCTCGACCCCCCTGCCATGGTGTGATCCG**C**AAAATAGGC**A**CCA**T**C**G**AAACGC	*Bsa* I	RB50		
Q1119	CGCGTCGACCATCCCGTCTTCCCCTCTGCGTTT	*Sal* I			
Q1048	CGCGAATTCCCAGCGCTGGGCCGGCGTC	*EcoR* I	RB50	pSS3967	pQC1363
Q1261	CGCGGTCTCGGGGTCGGCACAGTCTGGACGGCGACCGGTA	*Bsa* I			
Q1255	CGCGGTCTCGACCCCCCTGCCATGGTGTGATCCG**C**AAAATAGGC**A**CCA**C**C**A**AAACGC	*Bsa* I	RB50		
Q1119	CGCGTCGACCATCCCGTCTTCCCCTCTGCGTTT	*Sal* I			
Q1048	CGCGAATTCCCAGCGCTGGGCCGGCGTC	*EcoR* I	RB50	pSS3967	pQC1364
Q1261	CGCGGTCTCGGGGTCGGCACAGTCTGGACGGCGACCGGTA	*Bsa* I			
Q1256	CGCGGTCTCGACCCCCCTGCCATGGTGTGATCCG**C**AAAATAGGC**G**CCA**T**C**A**AAACGC	*Bsa* I	RB50		
Q1119	CGCGTCGACCATCCCGTCTTCCCCTCTGCGTTT	*Sal* I			
Q1048	CGCGAATTCCCAGCGCTGGGCCGGCGTC	*EcoR* I	RB50	pSS3967	pQC1365
Q1261	CGCGGTCTCGGGGTCGGCACAGTCTGGACGGCGACCGGTA	*Bsa* I			
Q1257	CGCGGTCTCGACCCCCCTGCCATGGTGTGATCCG**T**AAAATAGGC**A**CCA**T**C**G**AAACGC	*Bsa* I	RB50		
Q1119	CGCGTCGACCATCCCGTCTTCCCCTCTGCGTTT	*Sal* I			
Q1048	CGCGAATTCCCAGCGCTGGGCCGGCGTC	*EcoR* I	RB50	pSS3967	pQC1366
Q1261	CGCGGTCTCGGGGTCGGCACAGTCTGGACGGCGACCGGTA	*Bsa* I			
Q1258	CGCGGTCTCGACCCCCCTGCCATGGTGTGATCCG**T**AAAATAGGC**A**CCA**C**C**A**AAACGC	*Bsa* I	RB50		
Q1119	CGCGTCGACCATCCCGTCTTCCCCTCTGCGTTT	*Sal* I			
Q1048	CGCGAATTCCCAGCGCTGGGCCGGCGTC	*EcoR* I	RB50	pSS3967	pQC1367
Q1261	CGCGGTCTCGGGGTCGGCACAGTCTGGACGGCGACCGGTA	*Bsa* I			
Q1259	CGCGGTCTCGACCCCCCTGCCATGGTGTGATCCG**T**AAAATAGGC**G**CCA**T**C**A**AAACGC	*Bsa* I	RB50		
Q1119	CGCGTCGACCATCCCGTCTTCCCCTCTGCGTTT	*Sal* I			
Q1048	CGCGAATTCCCAGCGCTGGGCCGGCGTC	*EcoR* I	RB50	pSS3967	pQC1368
Q1261	CGCGGTCTCGGGGTCGGCACAGTCTGGACGGCGACCGGTA	*Bsa* I			
Q1260	CGCGGTCTCGACCCCCCTGCCATGGTGTGATCCG**C**AAAATAGGC**A**CCA**T**C**A**AAACGC	*Bsa* I	RB50		
Q1119	CGCGTCGACCATCCCGTCTTCCCCTCTGCGTTT	*Sal* I			
Q1048	CGCGAATTCCCAGCGCTGGGCCGGCGTC	*EcoR* I	BP536	pSS3967	pQC1369
Q1276	CGCGGTCTCGGGGTCAGCACGGTCCGGACGGTGACCGGTA	*Bsa* I			
Q1262	CGCGGTCTCGACCCCCCTGCCATGGTGTGATCCG**C**AAAATAGGC**A**CCA**T**C**A**AAACGC	*Bsa* I	BP536		
Q1119	CGCGTCGACCATCCCGTCTTCCCCTCTGCGTTT	*Sal* I			
Q1048	CGCGAATTCCCAGCGCTGGGCCGGCGTC	*EcoR* I	BP536	pSS3967	pQC1370
Q1276	CGCGGTCTCGGGGTCAGCACGGTCCGGACGGTGACCGGTA	*Bsa* I			
Q1263	CGCGGTCTCGACCCCCCTGCCATGGTGTGATCCG**T**AAAATAGGC**G**CCA**T**C**A**AAACGC	*Bsa* I	BP536		
Q1119	CGCGTCGACCATCCCGTCTTCCCCTCTGCGTTT	*Sal* I			
Q1048	CGCGAATTCCCAGCGCTGGGCCGGCGTC	*EcoR* I	BP536	pSS3967	pQC1371
Q1276	CGCGGTCTCGGGGTCAGCACGGTCCGGACGGTGACCGGTA	*Bsa* I			
Q1264	CGCGGTCTCGACCCCCCTGCCATGGTGTGATCCG**T**AAAATAGGC**A**CCA**C**C**A**AAACGC	*Bsa* I	BP536		
Q1119	CGCGTCGACCATCCCGTCTTCCCCTCTGCGTTT	*Sal* I			
Q1048	CGCGAATTCCCAGCGCTGGGCCGGCGTC	*EcoR* I	BP536	pSS3967	pQC1372
Q1276	CGCGGTCTCGGGGTCAGCACGGTCCGGACGGTGACCGGTA	*Bsa* I			
Q1265	CGCGGTCTCGACCCCCCTGCCATGGTGTGATCCG**T**AAAATAGGC**A**CCA**T**C**G**AAACGC	*Bsa* I	BP536		
Q1119	CGCGTCGACCATCCCGTCTTCCCCTCTGCGTTT	*Sal* I			
Q1048	CGCGAATTCCCAGCGCTGGGCCGGCGTC	*EcoR* I	BP536	pSS3967	pQC1373
Q1276	CGCGGTCTCGGGGTCAGCACGGTCCGGACGGTGACCGGTA	*Bsa* I			
Q1266	CGCGGTCTCGACCCCCCTGCCATGGTGTGATCCG**C**AAAATAGGC**G**CCA**T**C**A**AAACGC	*Bsa* I	BP536		
Q1119	CGCGTCGACCATCCCGTCTTCCCCTCTGCGTTT	*Sal* I			
Q1048	CGCGAATTCCCAGCGCTGGGCCGGCGTC	*EcoR* I	BP536	pSS3967	pQC1374
Q1276	CGCGGTCTCGGGGTCAGCACGGTCCGGACGGTGACCGGTA	*Bsa* I			
Q1267	CGCGGTCTCGACCCCCCTGCCATGGTGTGATCCG**C**AAAATAGGC**A**CCA**C**C**A**AAACGC	*Bsa* I	BP536		
Q1119	CGCGTCGACCATCCCGTCTTCCCCTCTGCGTTT	*Sal* I			
Q1048	CGCGAATTCCCAGCGCTGGGCCGGCGTC	*EcoR* I	BP536	pSS3967	pQC1375
Q1276	CGCGGTCTCGGGGTCAGCACGGTCCGGACGGTGACCGGTA	*Bsa* I			
Q1268	CGCGGTCTCGACCCCCCTGCCATGGTGTGATCCG**C**AAAATAGGC**A**CCA**T**C**G**AAACGC	*Bsa* I	BP536		
Q1119	CGCGTCGACCATCCCGTCTTCCCCTCTGCGTTT	*Sal* I			
Q1048	CGCGAATTCCCAGCGCTGGGCCGGCGTC	*EcoR* I	BP536	pSS3967	pQC1376
Q1276	CGCGGTCTCGGGGTCAGCACGGTCCGGACGGTGACCGGTA	*Bsa* I			
Q1269	CGCGGTCTCGACCCCCCTGCCATGGTGTGATCCG**T**AAAATAGGC**G**CCA**C**C**A**AAACGC	*Bsa* I	BP536		
Q1119	CGCGTCGACCATCCCGTCTTCCCCTCTGCGTTT	*Sal* I			
Q1048	CGCGAATTCCCAGCGCTGGGCCGGCGTC	*EcoR* I	BP536	pSS3967	pQC1377
Q1276	CGCGGTCTCGGGGTCAGCACGGTCCGGACGGTGACCGGTA	*Bsa* I			
Q1270	CGCGGTCTCGACCCCCCTGCCATGGTGTGATCCG**T**AAAATAGGC**G**CCA**T**C**G**AAACGC	*Bsa* I	BP536		
Q1119	CGCGTCGACCATCCCGTCTTCCCCTCTGCGTTT	*Sal* I			
Q1048	CGCGAATTCCCAGCGCTGGGCCGGCGTC	*EcoR* I	BP536	pSS3967	pQC1378
Q1276	CGCGGTCTCGGGGTCAGCACGGTCCGGACGGTGACCGGTA	*Bsa* I			
Q1271	CGCGGTCTCGACCCCCCTGCCATGGTGTGATCCG**T**AAAATAGGC**A**CCA**C**C**G**AAACGC	*Bsa* I	BP536		
Q1119	CGCGTCGACCATCCCGTCTTCCCCTCTGCGTTT	*Sal* I			
Q1048	CGCGAATTCCCAGCGCTGGGCCGGCGTC	*EcoR* I	BP536	pSS3967	pQC1379
Q1276	CGCGGTCTCGGGGTCAGCACGGTCCGGACGGTGACCGGTA	*Bsa* I			
Q1272	CGCGGTCTCGACCCCCCTGCCATGGTGTGATCCG**C**AAAATAGGC**G**CCA**C**C**A**AAACGC	*Bsa* I	BP536		
Q1119	CGCGTCGACCATCCCGTCTTCCCCTCTGCGTTT	*Sal* I			
Q1048	CGCGAATTCCCAGCGCTGGGCCGGCGTC	*EcoR* I	BP536	pSS3967	pQC1380
Q1276	CGCGGTCTCGGGGTCAGCACGGTCCGGACGGTGACCGGTA	*Bsa* I			
Q1273	CGCGGTCTCGACCCCCCTGCCATGGTGTGATCCG**C**AAAATAGGC**G**CCA**T**C**G**AAACGC	*Bsa* I	BP536		
Q1119	CGCGTCGACCATCCCGTCTTCCCCTCTGCGTTT	*Sal* I			
Q1048	CGCGAATTCCCAGCGCTGGGCCGGCGTC	*EcoR* I	BP536	pSS3967	pQC1381
Q1276	CGCGGTCTCGGGGTCAGCACGGTCCGGACGGTGACCGGTA	*Bsa* I			
Q1274	CGCGGTCTCGACCCCCCTGCCATGGTGTGATCCG**C**AAAATAGGC**A**CCA**C**C**G**AAACGC	*Bsa* I	BP536		
Q1119	CGCGTCGACCATCCCGTCTTCCCCTCTGCGTTT	*Sal* I			
Q1048	CGCGAATTCCCAGCGCTGGGCCGGCGTC	*EcoR* I	BP536	pSS3967	pQC1382
Q1276	CGCGGTCTCGGGGTCAGCACGGTCCGGACGGTGACCGGTA	*Bsa* I			
Q1275	CGCGGTCTCGACCCCCCTGCCATGGTGTGATCCG**T**AAAATAGGC**G**CCA**C**C**G**AAACGC	*Bsa* I	[illegible]		

Q1119	CGCGTCGACCATCCCGTCTTCCCCTCTGCGTTT	*Sal* I	[illegible]		
Q1048	CGCGAATTCCCAGCGCTGGGCCGGCGTC	*EcoR* I	pQC1285	pSS3967	pQC1514
Q1261	CGCGGTCTCGGGGTCGGCACAGTCTGGACGGCGACCGGTA	*Bsa* I			
Q1247	CGCGGTCTCGACCCCCCTGCCATGGTGTGATCCG**T**AAAATAGGC**G**CCA**C**C**G**AAACGC	*Bsa* I	pQC1285		
Q1119	CGCGTCGACCATCCCGTCTTCCCCTCTGCGTTT	*Sal* I			
Q1048	CGCGAATTCCCAGCGCTGGGCCGGCGTC	*EcoR* I	pQC1286	pSS3967	pQC1515
Q1261	CGCGGTCTCGGGGTCGGCACAGTCTGGACGGCGACCGGTA	*Bsa* I			
Q1247	CGCGGTCTCGACCCCCCTGCCATGGTGTGATCCG**T**AAAATAGGC**G**CCA**C**C**G**AAACGC	*Bsa* I	pQC1286		
Q1119	CGCGTCGACCATCCCGTCTTCCCCTCTGCGTTT	*Sal* I			
Q1048	CGCGAATTCCCAGCGCTGGGCCGGCGTC	*EcoR* I	pQC1287	pSS3967	pQC1516
Q1261	CGCGGTCTCGGGGTCGGCACAGTCTGGACGGCGACCGGTA	*Bsa* I			
Q1247	CGCGGTCTCGACCCCCCTGCCATGGTGTGATCCG**T**AAAATAGGC**G**CCA**C**C**G**AAACGC	*Bsa* I	pQC1287		
Q1119	CGCGTCGACCATCCCGTCTTCCCCTCTGCGTTT	*Sal* I			
Q1048	CGCGAATTCCCAGCGCTGGGCCGGCGTC	*EcoR* I	pQC1288	pSS3967	pQC1517
Q1261	CGCGGTCTCGGGGTCGGCACAGTCTGGACGGCGACCGGTA	*Bsa* I			
Q1247	CGCGGTCTCGACCCCCCTGCCATGGTGTGATCCG**T**AAAATAGGC**G**CCA**C**C**G**AAACGC	*Bsa* I	pQC1288		
Q1119	CGCGTCGACCATCCCGTCTTCCCCTCTGCGTTT	*Sal* I			
Q1048	CGCGAATTCCCAGCGCTGGGCCGGCGTC	*EcoR* I	pQC1289	pSS3967	pQC1518
Q1221	CGCGGTCTCGACGGTGACCGGTACCATCGCG	*Bsa* I			
Q1208	CGCGGTCTCGCCGTCCAGACTGTGCCGACCC	*Bsa* I	pQC1355		
Q1119	CGCGTCGACCATCCCGTCTTCCCCTCTGCGTTT	*Sal* I			
Q1048	CGCGAATTCCCAGCGCTGGGCCGGCGTC	*EcoR* I	pQC1290	pSS3967	pQC1519
Q1276	CGCGGTCTCGGGGTCAGCACGGTCCGGACGGTGACCGGTA	*Bsa* I			
Q1247	CGCGGTCTCGACCCCCCTGCCATGGTGTGATCCG**C**AAAATAGGC**A**CCA**C**C**G**AAACGC	*Bsa* I	pQC1290		
Q1119	CGCGTCGACCATCCCGTCTTCCCCTCTGCGTTT	*Sal* I			
Q1048	CGCGAATTCCCAGCGCTGGGCCGGCGTC	*EcoR* I	pQC1291	pSS3967	pQC1520
Q1276	CGCGGTCTCGGGGTCAGCACGGTCCGGACGGTGACCGGTA	*Bsa* I			
Q1262	CGCGGTCTCGACCCCCCTGCCATGGTGTGATCCG**C**AAAATAGGC**A**CCA**T**C**A**AAACGC	*Bsa* I	pQC1291		
Q1119	CGCGTCGACCATCCCGTCTTCCCCTCTGCGTTT	*Sal* I			
Q1048	CGCGAATTCCCAGCGCTGGGCCGGCGTC	*EcoR* I	pQC1292	pSS3967	pQC1521
Q1276	CGCGGTCTCGGGGTCAGCACGGTCCGGACGGTGACCGGTA	*Bsa* I			
Q1262	CGCGGTCTCGACCCCCCTGCCATGGTGTGATCCG**C**AAAATAGGC**A**CCA**T**C**A**AAACGC	*Bsa* I	pQC1292		
Q1119	CGCGTCGACCATCCCGTCTTCCCCTCTGCGTTT	*Sal* I			
Q1048	CGCGAATTCCCAGCGCTGGGCCGGCGTC	*EcoR* I	pQC1293	pSS3967	pQC1522
Q1276	CGCGGTCTCGGGGTCAGCACGGTCCGGACGGTGACCGGTA	*Bsa* I			
Q1262	CGCGGTCTCGACCCCCCTGCCATGGTGTGATCCG**C**AAAATAGGC**A**CCA**T**C**A**AAACGC	*Bsa* I	pQC1293		
Q1119	CGCGTCGACCATCCCGTCTTCCCCTCTGCGTTT	*Sal* I			
Q1048	CGCGAATTCCCAGCGCTGGGCCGGCGTC	*EcoR* I	pQC1294	pSS3967	pQC1523
Q1276	CGCGGTCTCGGGGTCAGCACGGTCCGGACGGTGACCGGTA	*Bsa* I			
Q1262	CGCGGTCTCGACCCCCCTGCCATGGTGTGATCCG**C**AAAATAGGC**A**CCA**T**C**A**AAACGC	*Bsa* I	pQC1294		
Q1119	CGCGTCGACCATCCCGTCTTCCCCTCTGCGTTT	*Sal* I			
Q1048	CGCGAATTCCCAGCGCTGGGCCGGCGTC	*EcoR* I	pQC1295	pSS3967	pQC1524
Q1209	CGCGGTCTCGACGGCGACCGGTACCATCGTGCGACT	*Bsa* I			
Q1220	CGCGGTCTCACCGTCCGGACCGTGCTGA	*Bsa* I	pQC1369		
Q1119	CGCGTCGACCATCCCGTCTTCCCCTCTGCGTTT	*Sal* I			
Q1048	CGCGAATTCCCAGCGCTGGGCCGGCGTC	*EcoR* I	pQC1296	pSS3967	pQC1525
Q1261	CGCGGTCTCGGGGTCGGCACAGTCTGGACGGCGACCGGTA	*Bsa* I			
Q1262	CGCGGTCTCGACCCCCCTGCCATGGTGTGATCCG**C**AAAATAGGC**A**CCA**T**C**A**AAACGC	*Bsa* I	pQC1366		
Q1119	CGCGTCGACCATCCCGTCTTCCCCTCTGCGTTT	*Sal* I			
Q1048	CGCGAATTCCCAGCGCTGGGCCGGCGTC	*EcoR* I	pQC1355	pSS3967	pQC1470
Q1370	TATGGTCTCGGCACAGTCTGGACGGCGACCGGTAC	*Bsa* I			
Q1368	TATGGTCTCTGTGC**T**GACCCCCCTGCCATGGTG	*Bsa* I	pQC1355		
Q1119	CGCGTCGACCATCCCGTCTTCCCCTCTGCGTTT	*Sal* I			
Q1048	CGCGAATTCCCAGCGCTGGGCCGGCGTC	*EcoR* I	pQC1355	pSS3967	pQC1471
Q1371	TATGGTCTCGGCAC**G**GTCTGGACGGCGACCGGTAC	*Bsa* I			
Q1369	TATGGTCTCTGTGCCGACCCCCCTGCCATGGTG	*Bsa* I	pQC1355		
Q1119	CGCGTCGACCATCCCGTCTTCCCCTCTGCGTTT	*Sal* I			
Q1048	CGCGAATTCCCAGCGCTGGGCCGGCGTC	*EcoR* I	pQC1355	pSS3967	pQC1472
Q1372	TATGGTCTCGGCACAGTC**C**GGACGGCGACCGGTAC	*Bsa* I			
Q1369	TATGGTCTCTGTGCCGACCCCCCTGCCATGGTG	*Bsa* I	pQC1355		
Q1119	CGCGTCGACCATCCCGTCTTCCCCTCTGCGTTT	*Sal* I			
Q1048	CGCGAATTCCCAGCGCTGGGCCGGCGTC	*EcoR* I	pQC1355	pSS3967	pQC1473
Q1375	TATGGTCTCGGTACCATCGTGCGACTTTGCGCCGAAG	*Bsa* I			
Q1373	TATGGTCTCGGTACCGGTC**A**CCGTCCAGACTGTG	*Bsa* I	pQC1355		
Q1119	CGCGTCGACCATCCCGTCTTCCCCTCTGCGTTT	*Sal* I			
Q1048	CGCGAATTCCCAGCGCTGGGCCGGCGTC	*EcoR* I	pQC1355	pSS3967	pQC1475
Q1377	TATGGTCTCGGTACCATCG**C**GCGACTTTGCGCCGAAG	*Bsa* I			
Q1374	TATGGTCTCGGTACCGGTCGCCGTCCAGACTGTG	*Bsa* I	pQC1355		
Q1119	CGCGTCGACCATCCCGTCTTCCCCTCTGCGTTT	*Sal* I			
Q1048	CGCGAATTCCCAGCGCTGGGCCGGCGTC	*EcoR* I	pQC1355	pSS3967	pQC1476
Q1380	TATGGTCTCTGTTGGATTGCAGTAGCGGGATGTG	*Bsa* I			
Q1378	TATGGTCTCCCAACACGGCG**T**GAACGCTCCTTC	*Bsa* I	pQC1355		
Q1119	CGCGTCGACCATCCCGTCTTCCCCTCTGCGTTT	*Sal* I			
Q1048	CGCGAATTCCCAGCGCTGGGCCGGCGTC	*EcoR* I	pQC1355	pSS3967	pQC1477
Q1380	TATGGTCTCTGTTGGATTGCAGTAGCGGGATGTG	*Bsa* I			
Q1379	TATGGTCTCCCAACACGGC**A**CGAACGCTCCTTC	*Bsa* I	pQC1355		
Q1119	CGCGTCGACCATCCCGTCTTCCCCTCTGCGTTT	*Sal* I			
Q1048	CGCGAATTCCCAGCGCTGGGCCGGCGTC	*EcoR* I	pQC1355	pSS3967	pQC1478
Q1383	TATGGTCTCGAATCGAGGGCTTTGTGCGACGCATC	*Bsa* I			
Q1381	TATGGTCTCCGATTCTTCCG**T**ACATCCCGCTACTG	*Bsa* I	pQC1355		
Q1119	CGCGTCGACCATCCCGTCTTCCCCTCTGCGTTT	*Sal* I			
Q1048	CGCGAATTCCCAGCGCTGGGCCGGCGTC	*EcoR* I	pQC1355	pSS3967	pQC1479
Q1384	TATGGTCTCGAATCGAGGG**T**TTTGTGCGACGCATC	*Bsa* I			
Q1382	TATGGTCTCCGATTCTTCCGCACATCCCGCTACTG	*Bsa* I	pQC1355		
Q1119	CGCGTCGACCATCCCGTCTTCCCCTCTGCGTTT	*Sal* I			
Q1048	CGCGAATTCCCAGCGCTGGGCCGGCGTC	*EcoR* I	pQC1355	pSS3967	pQC1480
Q1385	TATGGTCTCGAATCGAGGGCTTTGT**A**CGACGCATC	*Bsa* I			
Q1382	TATGGTCTCCGATTCTTCCGCACATCCCGCTACTG	*Bsa* I	pQC1355		
Q1119	CGCGTCGACCATCCCGTCTTCCCCTCTGCGTTT	*Sal* I			
Q1048	CGCGAATTCCCAGCGCTGGGCCGGCGTC	*EcoR* I	pQC1355	pSS3967	pQC1481
Q1390	TATGGTCTCCCGCATACGCGTTGGCGGTTG	*Bsa* I			
Q1386	TATGGTCTCATGCGCGCGGAT**T**CGTCGCAC	*Bsa* I	pQC1355		
Q1119	CGCGTCGACCATCCCGTCTTCCCCTCTGCGTTT	*Sal* I			
Q1048	CGCGAATTCCCAGCGCTGGGCCGGCGTC	*EcoR* I	pQC1355		
Q1390	TATGGTCTCCCGCATACGCGTTGGCGGTTG	*Bsa* I		pSS3967	pQC1482

Q1387	TATGGTCTCATGCGCGC**A**GATGCGTCGCAC	*Bsa* I		pSS3967	pQC1482
Q1119	CGCGTCGACCATCCCGTCTTCCCCTCTGCGTTT	*Sal* I	pQC1355		
Q1048	CGCGAATTCCCAGCGCTGGGCCGGCGTC	*EcoR* I	pQC1355	pSS3967	pQC1483
Q1390	TATGGTCTCCCGCATACGCGTTGGCGGTTG	*Bsa* I			
Q1388	TATGGTCTCATGCG**T**GCGGATGCGTCGCAC	*Bsa* I	pQC1355		
Q1119	CGCGTCGACCATCCCGTCTTCCCCTCTGCGTTT	*Sal* I			
Q1048	CGCGAATTCCCAGCGCTGGGCCGGCGTC	*EcoR* I	pQC1355	pSS3967	pQC1484
Q1391	TATGGTCTCCCGCAT**G**CGCGTTGGCGGTTG	*Bsa* I			
Q1389	TATGGTCTCATGCGCGCGGATGCGTCGCAC	*Bsa* I	pQC1355		
Q1119	CGCGTCGACCATCCCGTCTTCCCCTCTGCGTTT	*Sal* I			
Q1048	CGCGAATTCCCAGCGCTGGGCCGGCGTC	*EcoR* I	pQC1284	pSS3967	pQC1530
Q1385	TATGGTCTCGAATCGAGGGCTTTGT**A**CGACGCATC	*Bsa* I			
Q1382	TATGGTCTCCGATTCTTCCGCACATCCCGCTACTG	*Bsa* I	pQC1284		
Q1119	CGCGTCGACCATCCCGTCTTCCCCTCTGCGTTT	*Sal* I			
Q1048	CGCGAATTCCCAGCGCTGGGCCGGCGTC	*EcoR* I	pQC1530	pSS3967	pQC1531
Q1385	TATGGTCTCGAATCGAGGGCTTTGT**A**CGACGCATC	*Bsa* I			
Q1381	TATGGTCTCCGATTCTTCCG**T**ACATCCCGCTACTG	*Bsa* I	pQC1530		
Q1119	CGCGTCGACCATCCCGTCTTCCCCTCTGCGTTT	*Sal* I			
Q1048	CGCGAATTCCCAGCGCTGGGCCGGCGTC	*EcoR* I	pQC1531	pSS3967	pQC1532
Q1377	TATGGTCTCGGTACCATCG**C**GCGACTTTGCGCCGAAG	*Bsa* I			
Q1374	TATGGTCTCGGTACCGGTCGCCGTCCAGACTGTG	*Bsa* I	pQC1531		
Q1119	CGCGTCGACCATCCCGTCTTCCCCTCTGCGTTT	*Sal* I			
Q1048	CGCGAATTCCCAGCGCTGGGCCGGCGTC	*EcoR* I	pQC1532	pSS3967	pQC1533
Q1370	TATGGTCTCGGCACAGTCTGGACGGCGACCGGTAC	*Bsa* I			
Q1368	TATGGTCTCTGTGC**T**GACCCCCCTGCCATGGTG	*Bsa* I	pQC1532		
Q1119	CGCGTCGACCATCCCGTCTTCCCCTCTGCGTTT	*Sal* I			
Q1048	CGCGAATTCCCAGCGCTGGGCCGGCGTC	*EcoR* I	pQC1533	pSS3967	pQC1534
Q1390	TATGGTCTCCCGCATACGCGTTGGCGGTTG	*Bsa* I			
Q1386	TATGGTCTCATGCGCGCGGAT**T**CGTCGCAC	*Bsa* I	pQC1533		
Q1119	CGCGTCGACCATCCCGTCTTCCCCTCTGCGTTT	*Sal* I			
Q1048	CGCGAATTCCCAGCGCTGGGCCGGCGTC	*EcoR* I	pQC1480	pSS3967	pQC1536
Q1385	TATGGTCTCGAATCGAGGGCTTTGT**A**CGACGCATC	*Bsa* I			
Q1381	TATGGTCTCCGATTCTTCCG**T**ACATCCCGCTACTG	*Bsa* I	pQC1480		
Q1119	CGCGTCGACCATCCCGTCTTCCCCTCTGCGTTT	*Sal* I			
Q1048	CGCGAATTCCCAGCGCTGGGCCGGCGTC	*EcoR* I	pQC1536	pSS3967	pQC1537
Q1377	TATGGTCTCGGTACCATCG**C**GCGACTTTGCGCCGAAG	*Bsa* I			
Q1374	TATGGTCTCGGTACCGGTCGCCGTCCAGACTGTG	*Bsa* I	pQC1536		
Q1119	CGCGTCGACCATCCCGTCTTCCCCTCTGCGTTT	*Sal* I			
Q1048	CGCGAATTCCCAGCGCTGGGCCGGCGTC	*EcoR* I	pQC1537	pSS3967	pQC1538
Q1370	TATGGTCTCGGCACAGTCTGGACGGCGACCGGTAC	*Bsa* I			
Q1368	TATGGTCTCTGTGC**T**GACCCCCCTGCCATGGTG	*Bsa* I	pQC1537		
Q1119	CGCGTCGACCATCCCGTCTTCCCCTCTGCGTTT	*Sal* I			
Q1048	CGCGAATTCCCAGCGCTGGGCCGGCGTC	*EcoR* I	pQC1538	pSS3967	pQC1539
Q1390	TATGGTCTCCCGCATACGCGTTGGCGGTTG	*Bsa* I			
Q1386	TATGGTCTCATGCGCGCGGAT**T**CGTCGCAC	*Bsa* I	pQC1538		
Q1119	CGCGTCGACCATCCCGTCTTCCCCTCTGCGTTT	*Sal* I			
